# Bayesian multivariate linear mixed-effects models with varied association structures

**DOI:** 10.1177/09622802261455689

**Published:** 2026-06-30

**Authors:** Aglina Lika, Dimitris Rizopoulos, Michelle E Kruijshaar, Ans T van der Ploeg, Eleni-Rosalina Andrinopoulou

**Affiliations:** 1 Department of Biostatistics, 6993 Erasmus University Medical Center, Rotterdam, The Netherlands; 2 Department of Epidemiology, Erasmus University Medical Center, Rotterdam, The Netherlands; 3 Department of Pediatrics, Center for Lysosomal and Metabolic Diseases, Erasmus University Medical Center, Rotterdam, The Netherlands; 4 Statistics and Data Science Innovation Hub, GSK, The Netherlands

**Keywords:** Multivariate linear mixed-effects models, Bayesian analysis, Pompe disease, Hamiltonian Monte Carlo, Longitudinal data, Association structures

## Abstract

In medicine, multiple continuous outcomes are often repeatedly measured on each subject over time to assess disease severity. Usually, it is of interest to investigate the association between those outcomes, which may be measured at different time points, resulting in unbalanced data. The multivariate linear mixed-effects model (MLMM) is a popular framework for this analysis. It considers the unbalanced nature of the data and accounts for the association of the outcomes via the random effects, often assuming a multivariate normal distribution. However, measuring and understanding the degree of connection between longitudinal outcomes remains challenging. We propose to enhance the MLMM by incorporating various interpretable association structures. Specifically, we consider that multiple longitudinal outcomes are related to the primary outcome through their current value, cumulative effect (total or partial), or both. Our research is motivated by Pompe disease, a rare, inheritable, progressive metabolic myopathy. Clinically, it is important to investigate how patient-reported outcome measures (primary outcomes) are associated with physical outcomes to determine whether improvements in physical outcomes are accompanied by improvements in health-related quality of life and other patient experiences. We found a positive association between them. The proposed models are fitted under the Bayesian framework using Hamiltonian Monte Carlo.

## Abbreviations

ERT: enzyme replacement therapy

HMC: Hamiltonian Monte Carlo

## 1. Introduction

In numerous clinical studies, multiple longitudinal outcomes are collected at regular intervals from each subject to monitor the progression of disease severity. Improving decision-making in clinical settings requires a thorough investigation into the potential relationships among different outcomes. For instance, in prospective Pompe disease studies, which is a rare, inheritable, and progressive metabolic myopathy, the interest lies in the association between continuous physical outcomes and continuous patient-reported outcome measures (PROMs). Knowledge of how these outcomes are associated is essential for assessing their reflection in the patient’s health-related quality of life (hrQoL).

**Figure 1.**
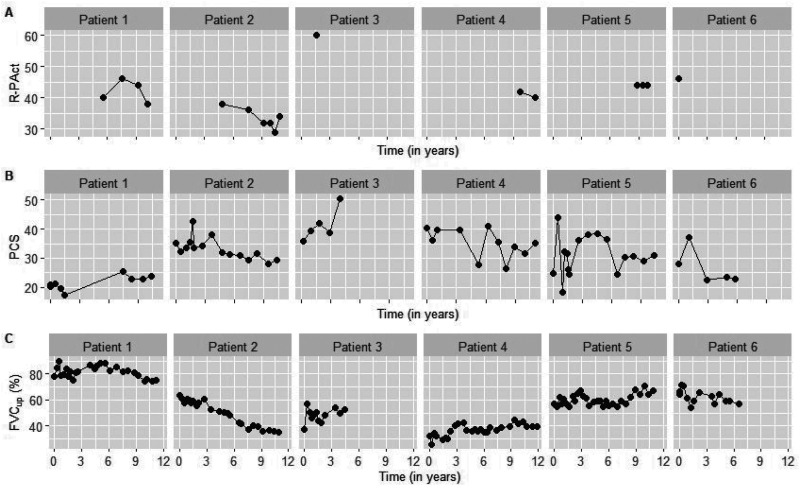
Plot of Forced vital Capacity in the upright seated position expressed as a percentage of its predicted values ( ${\text{FVC}}_{\text{up}}(\%$ )), Rasch-Built Pompe-Specific Activity scale (R-PAct) and physical component summary (PCS) score over time of six Dutch adult Pompe disease patients, respectively.

Our study is motivated by Pompe disease data obtained from two prospective follow-up studies conducted at the Center for Lysosomal and Metabolic Diseases, Erasmus MC University Medical Center, Rotterdam-the national referral center for Pompe disease in the Netherlands.^1,2^ Integrating such data presents several challenges. In Figure 1, we present the evolution of one of the primary clinical outcomes, Forced Vital Capacity in the upright seated position expressed as a percentage of its predicted values ( ${\text{FVC}}_{\text{up}}(\%$ )), and of two PROMs, the Physical Component summary (PCS) score from the Medical Outcome Study 36-item Short-Form Health Survey^3^ [SF − 36] and the Rasch-Built Pompe-Specific Activity (R-PAct) scale, over time for six randomly selected Dutch adult Pompe disease patients. Notably, the measurements of these three continuous outcomes vary in frequency and occur at different time points. In common clinical practice, the PROMs are not always measured as often as the ${\text{FVC}}_{\text{up}}(\%)$ and are measured at different time points, resulting in unbalanced data sets. The rationale for focusing on these outcomes is provided in a previous publication.^4^

The multivariate linear mixed-effects model (MLMM) is a popular framework for jointly analyzing multiple continuous longitudinal outcomes while accounting for the unbalanced nature of the data. This model accounts for the association between the longitudinal outcomes through the random effects, often assuming a multivariate normal distribution.^5–11^ Computational challenges may appear due to the dimension of the variance–covariance matrix of the joint random effects when the number of outcomes and (or) the number of random effects per outcome increases. Fieuws and Verbeke^12^ proposed using the pairwise approach for modeling four or more outcomes where the standard MLMM would become computationally challenging. In particular, they suggested fitting all possible bi-variate models and the average over the duplicate parameter estimates to be calculated. This approach offers greater flexibility than assuming the complete multivariate linear mixed-effects model, potentially resulting in increased variability and some efficiency loss for the shared parameters. Another method to address the association among multiple longitudinal outcomes is to link the error terms rather than the random effects.^5,13–17^ However, this can lead to challenges related to high dimensionality, especially when assuming numerous longitudinal outcomes or complex random effects structures. Moreover, connecting the outcomes via error terms might pose difficulties when measurements are taken at dissimilar time points.

Motivated by questions regarding Pompe disease, our research aims to establish relationships between continuous longitudinal outcomes from different data sources. While the aforementioned methods connect the different outcomes, a direct interpretation of the association parameters is not always feasible. It is crucial for clinicians to quantify the relationship between the outcomes. Moreover, the degree of association of random effects does not necessarily imply a clinical improvement reflective of the patient’s condition, especially in cases where complex structures are assumed. One possible approach to quantifying and interpreting the relationship between outcomes is to include the functional forms of one outcome as endogenous covariates in the model for the primary outcome of interest. This solution offers flexibility by allowing for different specifications of the outcomes that align with their biological interpretation. A time-varying covariate is considered endogenous if its value at time t is not conditionally independent of all preceding outcome measurements, meaning prior outcome values impact it;^18^ otherwise, it is exogenous.^19–21^ When endogenous variables are included in a linear mixed-effects model, the parameters have only conditional interpretation since the expected value of the random effects given the covariates is not zero. When only exogenous variables are included, there is no discrepancy between the marginal and conditional interpretation of the parameters; that is, the marginal mean of the outcome is equal to its conditional mean given the covariates and the random effects.^19^ Erler et al. found that misspecifying endogenous variables as exogenous leads to biased estimates, while the opposite assumption yields unbiased estimates.^22^

It has been previously recognized that different ways exist to associate an outcome with a time-varying covariate, for example, by relating them at the same time point or assuming a lag effect between the covariate and the impact on the outcome.^18^ Different association structures are investigated and extensively discussed in the literature of joint models for longitudinal and survival data.^23,24^ The longitudinal outcomes can be related via the random effects and connected to the hazard, assuming different functional forms. In particular, their current value at a specific time point t , their current slopes of the trajectories at time t , and the total cumulative effect until the time t are commonly included as endogenous covariates. In practice, deciding which functional forms to assume is based on the clinical interest. McCrink et al.^25^ stated that the choice of the association structure should reflect the study focus. Moreover, it is possible to include multiple functional forms if no information is known about the connection of the outcomes.^23^ Little research has been done on the link between the longitudinal outcomes in the multivariate mixed-effects models framework. Van Oudenhoven et al.^26^ suggested a multivariate joint model utilizing prodromal Alzheimer’s disease trial data and incorporating additional connections between the longitudinal outcomes. In particular, the two continuous longitudinal outcomes of interest (hippocampal brain atrophy and memory impairment ratings) were interrelated and linked to two events: time to open-label medication and dropout. Linear mixed-effects models were fitted for each longitudinal outcome to estimate their trajectories. Additionally, in the model for hippocampal brain atrophy, the linear predictor from the memory impairment ratings model was included as an endogenous covariate. Delporte et al. assumed a joint normal-ordinal model and proposed a correlation function to capture the association between an ordinal (level of impairment) and a continuous (functioning score) longitudinal outcome.^27^

To investigate the strength of the association between the continuous longitudinal outcome ${\text{FVC}}_{\text{up}}(\%)$ and PROMs (PCS and R-PAct) in Dutch adult patients with Pompe disease over time, we rely on the multivariate mixed-effects models framework and go beyond the standard formulation of connecting the random effects. In particular, we propose an extension of the multivariate linear mixed-effects model, and we postulate different association structures that reflect the biological assumptions under the Bayesian framework using Hamiltonian Monte Carlo (HMC). In the application, we include functional forms of ${\text{FVC}}_{\text{up}}(\%)$ as endogenous covariates in the models of the PROMs, which are of primary interest and are directly affected by the clinical condition of the patients. The functional forms assumed in the present study are the current value (linear predictor), the total or partial cumulative effect (area under the curve) of the longitudinal predictors, and their combination. This is in line with the clinical belief that the current value of the ${\text{FVC}}_{\text{up}}(\%)$ and its whole or partial history affects the current value of the PROMS. Finally, we conduct a simulation study to validate the proposed models and explore whether any bias is introduced when we misspecify the relationship between the random effects or ignore the functional form between the longitudinal outcomes. Including endogenous variables can result in causal inferential challenges. The existence of an association between two or more longitudinal outcomes does not imply causation. Causal claims cannot be proved from associations alone. Behind every causal conclusion, some causal assumptions must lie that are not testable in observational studies.^28,29^ Therefore, this work primarily focuses on the association between multiple outcomes and not on causal relationships.

This article is constructed as follows: Section 2 introduces the standard multivariate linear mixed-effects model and extensions of it by assuming different association structures between the longitudinal outcomes. Section 3 presents the Bayesian approach, including the likelihood and priors of the parameters. An application of the extended multivariate linear mixed-effects model to the Pompe disease data is given in Section 4. Section 5 presents our simulation study and its results. Section 6 contains a discussion.

## 2. Mixed-effects model

In many studies, multiple repeated measurements are collected from each subject for various types of outcomes, which may be recorded at different time points. Mixed effects models are commonly used to analyze longitudinal data and can be extended to address multiple longitudinal outcomes.

### 2.1. Multivariate linear mixed-effects model

The multivariate mixed-effects model accounts for the association between multiple longitudinal outcomes through the random effects, often by assuming a multivariate normal distribution. Motivated by our application on Pompe disease data, where both the clinical outcome and the patient-reported outcomes are measured on a continuous scale, we focus on the multivariate linear mixed-effects model. Let’s assume that we have K longitudinal outcomes from N subjects to model jointly. We denote as $y_{i,k}={\left(y_{i1,k},\ldots ,y_{in_{i},k}\right)}^{⊤}$ the $n_{i,k}$ -dimensional vector of observations of the k -th outcome of the i -th subject ( k=1,…,K and i=1,…,N ) taken at each time $t_{ij,k}$ ( $j=1,\ldots ,n_{i,k}$ ) and as $y_{i,k}(t)$ the measurement of the k -th outcome of the i -th subject taken at time t . The subject-specific evolution over time of each of the k continuous longitudinal outcomes can be then described as:

$$y_{i,k}(t)=x_{i,k}^{⊤}(t)\beta_{k}+z_{i,k}^{⊤}(t)b_{i,k}+\epsilon_{i,k}(t)=m_{i,k}(t)+\epsilon_{i,k}(t)$$

where $x_{i,k}^{⊤}(t)$ denotes the design vector for the fixed effects regression coefficients $\beta_{k}$ and $z_{i,k}^{⊤}(t)$ denotes the design vector for the random effects $b_{i,k}$ . For the stacked vector of the subject-specific random effects for all K outcomes $b_{i}^{⊤}=(b_{i,1}^{⊤},\ldots ,b_{i,K}^{⊤})$ , it is assumed that it follows a multivariate normal distribution with mean zero and variance–covariance matrix D . The error terms $\epsilon_{i,k}(t)$ are mutually independent, independent of the random effects and normally distributed with mean zero and variance $\sigma_{k}^{2}$ .

Connecting longitudinal outcomes through random effects does not directly measure the strength of the association and lacks clinical relevance, especially when a complex random effects structure is involved. Therefore, in many applications, one of the outcomes is considered the primary outcome of interest, while the other outcomes serve as time-varying predictors. Without loss of generality, we assume the K -th outcome as the primary outcome and establish its connection with the other outcomes by assuming various association structures.

**Figure 2.**
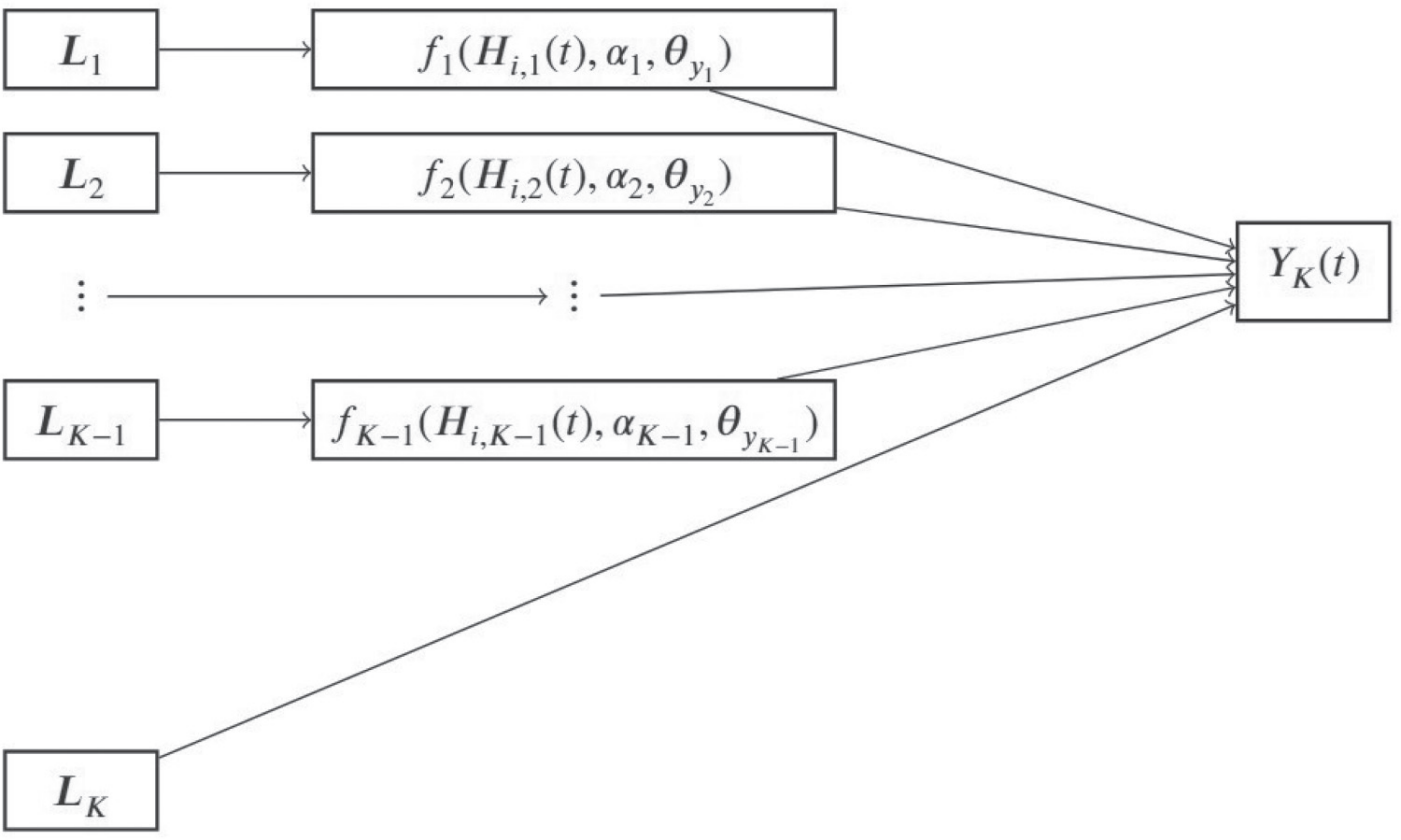
Graph of associations between K longitudinal outcomes and sets of confounders ( $L_{1},\ldots ,L_{K}$ ) of the study population. The $f_{k}(\cdot )$ ( k=1,…,K−1 ) is the functional form of the $Y_{k}$ longitudinal outcome that associates it with the primary outcome $Y_{K}$ .

In particular, we assume different functional forms of the longitudinal outcomes $y_{i,k},\ldots ,y_{i,(K-1)}$ to be included in the model of the longitudinal outcome $y_{i,K}$ as endogenous covariates. Figure 2 illustrates the relationships between the different outcomes of interest. Additional information on the study population can be included as independent variables in each submodel of the longitudinal outcomes, for example, baseline variables such as sex. The nodes represent the functional form of every outcome, denoted as $f_{1}(H_{i,1}(t),\alpha_{1},\theta_{y_{1}}),\ldots ,f_{K-1}(H_{i,K-1}(t),\alpha_{K-1},\theta_{y_{K-1}})$ , connected with the primary outcome ( $Y_{K}$ ) and sets of baseline covariates (denoted as $L_{1},\ldots ,L_{K}$ ) that may be included in each submodel. These covariates may remain consistent or vary across each submodel. The arrows signify relationships between the nodes rather than indicating causal direction.

Given the above, the proposed multivariate linear mixed-effects model of the primary outcome takes the form:

$$y_{i,K}(t)=m_{i,K}(t)+\sum_{q=1}^{K-1}f_{q}(H_{i,q}(t),\alpha_{q},\theta_{y_{q}})+\epsilon_{i,K}(t)$$

The function $f_{q}(H_{i,q}(t),\alpha_{q},\theta_{y_{q}})$ denotes the functional form of the q -th longitudinal outcome that is included in the submodel of the primary outcome ( q=1,…,K−1 ). The $H_{i,q}(t)=\{m_{i,q}(s),0\leq s<t\}$ denotes the history of the q -th true and unobserved longitudinal outcome up to time point t ( q=1,…,K−1 ), $\alpha_{q}$ quantifies the strength of the association between the q -th longitudinal outcome and the primary outcome, and $\theta_{y_{q}}$ are the parameters related to the q -th longitudinal outcome.

Connecting the outcomes through different functional forms and random effects may lead to computational difficulties and make it challenging to interpret their associations, hence assuming independent (unstacked random effects vector) may be preferred and then $b_{i,k}^{⊤}\;∼\text{MVN}(0,D_{k})$ where k=1,…,K .

### 2.2. Association structures

In longitudinal studies with time-varying covariates, several challenges emerge regarding their modeling. From a clinical perspective, accurately characterizing the association is important to reflect biological assumptions, as it can significantly impact the study results. Thus, it is essential to determine which summary measure of the covariate is linked to the outcome of interest. Table 1 summarizes various forms of associations guided by our clinical context. Each of the q -th outcomes may be associated with the primary outcome.

**Table 1.** Functional forms of longitudinal outcome(s) associated with the outcome of main interest in the multivariate linear mixed-effects model.

Parameterization	Latent association
1. Current value (linear predictor)	$f_{q}(H_{i,q}(t),\alpha_{q},\theta_{y_{q}})=\alpha_{q}m_{i,q}(t)$
2. Cumulative effect (area under the trajectory) from ** $t_{0}$ ** to ** t **	$f_{q}(H_{i,q}(t),\alpha_{q},\theta_{y_{q}})=\alpha_{q}\int_{t_{0}}^{t}m_{i,q}(s)ds$
3. Combination of 1 and 2	$f_{q}(H_{i,q}(t),\alpha_{q},\theta_{y_{q}})=\alpha_{1q}m_{i,q}(t)+\alpha_{2q}\int_{t_{0}}^{t}m_{i,q}(s)ds$

A straightforward way to summarize a longitudinal outcome is to assume its underlying value at a specific time point. In this context, the underlying values of the longitudinal outcomes $y_{i,1},\ldots ,y_{i,(K-1)}$ at time t , can be included as linear predictors in the model of the primary outcome.^26^ This approach allows for linking the current value or any past value (lag effect) to the primary outcome. In some cases, a lag effect (i.e., at time $t-t_{1}$ , $t_{1}<t$ ) may be more biologically relevant, especially when there is a time gap between the onset of symptoms and disease diagnosis. The lag effect of interest can be defined with the help of medical professionals. If they are uncertain, then a model that includes multiple potential lag effects can be fitted, and their importance can be assessed by selection methods (e.g., lasso, ridge) and selection criteria.

The present value holds biological significance for our application and can be readily understood by clinicians. This association parameter interprets that a unit increase in the q -th longitudinal outcome at t (or $t-t_{1}$ ) is associated with the value of the primary outcome at time t .

In specific research contexts, relying solely on a single value may not adequately capture the overall outcomes. For patients with Pompe disease, consistently low physical scores over an extended period (such as six months to a year) can hold more clinical significance than isolated instances of low scores. For example, prolonged periods of physical pain or fatigue have a greater impact on patient-reported outcomes compared to brief moments of severe pain. Therefore, incorporating historical data on outcomes provides deeper insights into disease progression and better informs physicians about the effectiveness of interventions. Thus, we can assume that the longitudinal outcomes $y_{i,1},\ldots ,y_{i,(K-1)}$ are associated with the primary outcome via their total cumulative effects (areas under the trajectories) of the longitudinal processes from $t_{0}=0$ to t or the partial areas under the trajectories from $t_{0}=t-t_{1}$ to t ( $t_{1}<t$ ) assuming that all values have the same influence.

In alternative contexts, varying functional forms may be more suitable for modelling the relationship between longitudinal outcomes and the primary outcome. For example, considering the trajectory’s slope (i.e., the rate of change of an outcome relative to another) or weighted cumulative effect (assuming that the more recent values have a greater influence)^30,31^ could be relevant. Additionally, in certain clinical applications, a combination of different functional forms and association parameters might be applicable. The choice of association structure(s) should align with biological assumptions**.**

## 3. Bayesian approach

### 3.1. Estimation and likelihood

Under the Bayesian framework and using the Hamiltonian Monte Carlo (HMC) algorithm,^32–36^ we estimate the parameters of the model and the inference is based on the posterior distribution of these parameters. If $\Theta =(\beta_{k},\alpha_{q},\sigma_{k}^{2},D)$ , the likelihood of the model will be

$$L(\Theta )=\prod_{i=1}^{N}L_{i}(\Theta )$$

where $L_{i}(\Theta )$ is the contribution of the i - th individual in the likelihood of the multivariate mixed-effects model. Further details are provided in the supplemental material Part A.

### 3.2. Priors, posterior and diagnostics

We assume that the parameters $\Theta =(\beta_{k},\alpha_{q},\sigma_{k}^{2},D)$ have the following priors: the coefficients of the fixed effects $\beta_{k}∼N(0,\sigma_{\beta_{k}}^{2}I)$ , the association coefficients $\alpha_{q}∼N(0,\sigma_{\alpha_{q}}^{2})$ and the standard deviation of the error term $\sigma_{k}∼$ half-Cauchy(0,b). For the standard deviations of the varince-covarince matrix D of the random effects, we assume $\sigma_{b_{i}}∼$ half-Cauchy(0,d) and for the correlation matrix of the random effects (let’s denote it R ), we assume R∼ lkj( η ). The Lewandowski–Kurowicka–Joe (lkj) distribution^37^ with scaling parameter η is commonly used for the correlation matrix of the random effects. The posterior distribution takes the form:

$$P(\Theta |y)\propto L(\Theta )P(\Theta )=\prod_{i=1}^{N}\prod_{k=1}^{K}\prod_{q=1}^{K-1}L_{i}(\Theta )f(\beta_{k})f(\alpha_{q})f(\sigma_{k})f(\sigma_{b_{i}})f(L),$$

where f() denotes the prior probability density function. To evaluate the convergence of chains toward the posterior distribution, we employ the $\hat{R}$ statistic and trace plots.^60,61^ In our simulation study, we compute the bias and mean square error (MSE) of the parameters. The MSE represents the expected value of the squared error loss, quantifying the average squared difference between estimated and actual values.^59^ A well-performing model exhibits low bias and MSE values.

## 4. Application

### 4.1. Data

Our motivation stems from two prospective observational cohort studies conducted at the Center for Lysosomal and Metabolic Diseases, Erasmus MC University Medical Center in Rotterdam—the national referral center for Pompe disease in the Netherlands.^1,2^ Pompe disease, a rare, progressive metabolic myopathy, has been the focus of research. Since 2006, Enzyme Replacement Therapy (ERT) with recombinant human alpha-glucosidase (alglucosidase alfa, Myozyme) has been approved as a treatment for Pompe disease. Notably, ERT has demonstrated beneficial effects on various aspects, including physical outcomes (such as motor performance, muscle strength, and pulmonary function), patient-reported outcomes (PROMs), and overall survival.^38–44^

Multiple longitudinal physical and patient-reported outcomes are available from the two prospective observational cohort studies. Data are combined, and only data from Dutch adult patients with Pompe disease included in both studies, treated with ERT and assessed for physical outcomes during ERT (population of study interest) are retained. One of the most important physical outcomes is Forced Vital Capacity in the upright seated position, ${\text{FVC}}_{\text{up}}(\%)$ , which measures the air exhaled from the lungs and is expressed as a percentage of predicted normal values based on the subject’s age, sex, race, and height.^45,46^ The PROMs, which are essential in evaluating the effects of non-life-saving treatments, are collected annually through an ongoing international questionnaire study: the IPA/Erasmus MC Pompe survey,^2^ but also partly alongside the clinical follow-up. Two PROMs are of interest: the physical component summary (PCS) score, which is derived from the Medical Outcome Study 36-item Short-Form Health Survey^3^ (SF − 36, versions 1 and 2) that assesses the quality of life and the Rasch-Built Pompe-Specific Activity (R-PAct) scale,^47^ which is the only Pompe-specific PROM and assesses the patient’s ability to carry out daily life activities. The rationale for focusing on these outcomes is provided in a previous publication.^4^

In previous work, Yuan et al.^48^ found that ${\text{FVC}}_{\text{up}}(\%)$ was related to patient-reported outcome measures (PROMs) at the start of enzyme replacement therapy (ERT) for late-onset Pompe disease. Building on this, our investigation explored whether ${\text{FVC}}_{\text{up}}(\%)$ remains associated with these specific PROMs over time during ERT. We considered the physical domain of quality of life and daily life activities as primary outcomes, aiming to understand how they are affected by ${\text{FVC}}_{\text{up}}(\%)$ . Specifically, we examined the link between PROMs and the current ${\text{FVC}}_{\text{up}}(\%)$ value at a given time, as well as its cumulative effect up to that point. Three cumulative effect scenarios were considered: total cumulative effect, cumulative effect over the last six months and over the last year.

Table 2 presents some of the demographic and clinical characteristics of the Dutch adult patients with Pompe disease eligible for the analyses. For the extended multivariate linear mixed-effects model of the ${\text{FVC}}_{\text{up}}(\%)$ with the PCS and R-PAct, data are available from 100 and 94 patients, respectively. The two subpopulations have different numbers of subjects since the PROMs are not measured as frequently as the ${\text{FVC}}_{\text{up}}(\%)$ and subjects who lacked key information for the models (i.e., missing disease duration, age, sex, and/or date of measurement) and/or did not have PROMs or ${\text{FVC}}_{\text{up}}(\%)$ values at all were excluded from the analyses. However, the two subpopulations have similar characteristics with some minor deviations. In Figure 1, we observe that the patients have non-linear profiles over time for all the longitudinal outcomes; thus, in the linear mixed-effects submodels of the outcomes, we assume natural cubic splines for the effect of time with two degrees of freedom. In the specification of the splines, boundary knots are placed at the start of ERT ( t=0 ) and at 15.3 years (maximum observed time), and the internal knot is placed at the 50% observed follow-up time (at 2.78 for the PCS, 5.0 for the R-PAct and approximately at 5 years for the ${\text{FVC}}_{\text{up}}(\%)$ ). The same structure is also used in the random-effects part of the models to flexibly capture the correlation among the follow-up visits.

**Table 2.** Characteristics of the patients eligible for the multivariate linear mixed-effects model of the Forced vital capacity in the upright position expressed as percentage of its predicted values ( ${\text{FVC}}_{\text{up}}(\%)$ ) with the Physical Component Summary (PCS) score (n = 100) and the Rasch-Built Pompe-Specific Activity R-PAct scale (n = 94), respectively.

Demographic and clinical characteristic	Patients (n = 100)	Patients (n = 94)
Women: number ( % )	55(55%)	53(56.4%)
Age at start of ERT: median (range)	50.5(22−76)	50(19−73)
Age at start of symptoms (in years): median (range)	33.5(2−62)	34(3−62)
Disease duration from the symptom onset (in years): median (range)	14(0.85−50.3)	11.8(0.85−50.3)
Total follow-up time (in years): median (range)	9.4(0.5−15.4)	10(0.5−15.4)
${\text{FVC}}_{\text{up}}(\%)$ : median (range)	67(7−119.2)	68(12−119)
PCS: median (range)	32.2(14.7−56.3)	32.2(15.9−56.3)
R-PAct: median (range)	56(7−100)	56(7−100)

**Figure 3.**
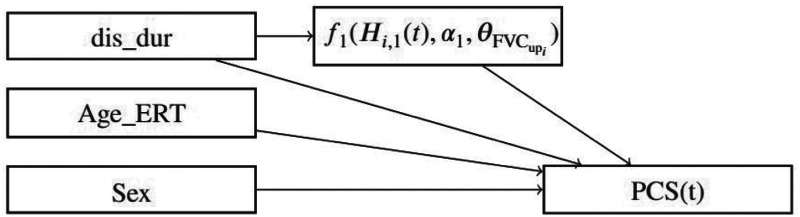
Graph illustrating the relationships between variables and outcomes within the context of Pompe disease. $f_{1}(H_{i,1}t),\alpha_{1},\theta_{{\text{FVC}}_{{\text{up}}_{i}}})$ : association structure of the Forced vital capacity in the upright seated position expressed as percentage of its predicted values ( ${\text{FVC}}_{\text{up}}(\%)$ ) with the physical component summary (PCS) score; Age_ERT : age at the start of Enzyme replacement therapy (ERT) (in years); dis_dur : disease duration at the start of ERT (in years); Sex: female/male.

**Figure 4.**
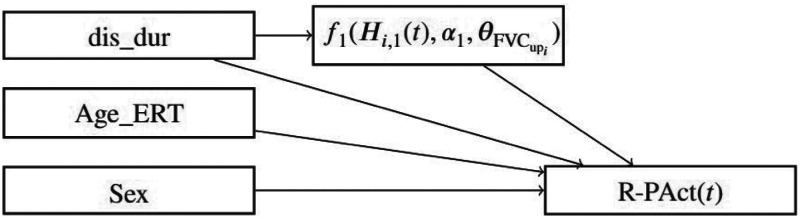
Graph illustrating the relationships between variables and outcomes within the context of Pompe disease. $f_{1}(H_{i,1}(t),\alpha_{1},\theta_{{\text{FVC}}_{{\text{up}}_{i}}})$ : association structure of the Forced vital capacity in the upright seated position expressed as percentage of its predicted values ( ${\text{FVC}}_{\text{up}}(\%)$ ) with the Rasch-Built Pompe-Specific Activity scale (R-PAct); Age_ERT : age at the start of Enzyme replacement therapy (ERT) (in years); dis_dur : disease duration at the start of ERT (in years); Sex: female/male.

In the model of PCS and R-PAct, we include the variables sex, the standardized age at the start of ERT ( Age_ERT ), and the standardized disease duration at the start of ERT ( dis_dur ). In the model of ${\text{FVC}}_{\text{up}}(\%)$ , we include only the standardized disease duration since the age and sex were considered to express the ${\text{FVC}}_{\text{up}}$ as the percentage of its predicted values. Figures 3 and 4 illustrate the associations of the outcomes mentioned above.

The submodel of the clinical outcome ${\text{FVC}}_{\text{up}}$ in the fitted multivariate linear mixed-effects model is

$$\begin{array}{rl} {\text{FVC}}_{{\text{up}}_{i}}(\%)(t) & =(\beta_{0,1}+b_{i0,1})+(\beta_{1,1}+b_{i1,1}){ns(t,2)}_{1}+(\beta_{2,1}+b_{i2,1}){ns(t,2)}_{2}+\beta_{3,1}\text{dis}\_{\text{dur}}_{i}(t)+\epsilon_{i,1}(t)\\ & =m_{i,1}(t)+\epsilon_{i,1}(t) \end{array}$$

and the submodels of the PROMs are

$$\begin{array}{rl} {\text{PCS}}_{i}(t) & =(\beta_{0,2}+b_{i0,2})+(\beta_{1,2}+b_{i1,2}){ns(t,2)}_{1}+(\beta_{2,2}+b_{i2,2}){ns(t,2)}_{2}+\beta_{3,2}\text{dis}\_{\text{dur}}_{i}(t)\\ & \;+\beta_{4,2}{\text{Age}\_\text{ERT}}_{i}(t)+\beta_{5,2}sex_{i}(t)+f_{1}(H_{i,1}(t),\alpha_{1},\theta_{{\text{FVC}}_{{\text{up}}_{i}}})+\epsilon_{i,2}\\ {\text{R-PACt}}_{i}(t) & =(\beta_{0,3}+b_{i0,3})+(\beta_{1,3}+b_{i1,3}){ns(t,2)}_{1}+(\beta_{2,3}+b_{i2,3}){ns(t,2)}_{2}+\beta_{3,3}\text{dis}\_{\text{dur}}_{i}(t)\\ & \;+\beta_{4,3}{\text{Age}\_\text{ERT}}_{i}(t)+\beta_{5,3}sex_{i}(t)+f_{1}(H_{i,1}(t),\alpha_{1},\theta_{{\text{FVC}}_{{\text{up}}_{i}}})+\epsilon_{i,3}(t) \end{array}$$

where $\beta_{0,1}$ **,** $\beta_{0,2}$ and $\beta_{0,3}$ are the intercepts of the submodels. The ${ns(t,2)}_{1}$ and ${ns(t,2)}_{2}$ is the value of the first and second component of the cubic splines of the time (in years) since the start of ERT of subject i with two degrees of freedom at time t , respectively. The $b_{i0,1}$ , $b_{i0,2}$ and $b_{i0,3}$ are the random intercepts of the models. As $\text{dis}\_{\text{dur}}_{i}(t)$ , $sex_{i}(t)$ and ${\text{Age}\_\text{ERT}}_{i}(t)$ , we denote the values of the standardized disease duration, sex, and standardized age at start of ERT of the subject i at time t . The $f_{1}(H_{i,1}(t),\alpha_{1},\theta_{{\text{FVC}}_{{\text{up}}_{i}}})$ is the functional form that describes the association structure between the ${\text{FVC}}_{\text{up}}(\%)$ and the two PROMs longitudinal outcomes and can be

(1) $$\begin{array}{rl} f_{1}(H_{i,1}(t),\alpha_{1},\theta_{{\text{FVC}}_{{\text{up}}_{i}}}) & =\alpha_{1}m_{i,1}(t) \end{array}$$

(2) $$\begin{array}{rl} f_{1}(H_{i,1}(t),\alpha_{1},\theta_{{\text{FVC}}_{{\text{up}}_{i}}}) & =\alpha_{1}\int_{t_{0}}^{t}m_{i,1}(s)ds \end{array}$$

(3) $$\begin{array}{rl} f_{1}(H_{i,1}(t),\alpha_{1},\theta_{{\text{FVC}}_{{\text{up}}_{i}}}) & =\alpha_{11}m_{i,1}(t)+\alpha_{21}\int_{t_{0}}^{t}m_{i,1}(s)ds \end{array}$$

where as $\alpha_{11}$ (or $\alpha_{1}$ ) and $\alpha_{21}$ (or $\alpha_{1}$ ), we define the coefficient of the current value at time t and the coefficient of the total or partial cumulative effect, respectively. The $t_{0}=0$ when we do not have a lag effect and $t_{0}=t-t_{1}$ when we have a lag effect of size $t_{1}$ . For a lag effect of 6 and 12 months, we have $t_{0}=t-0.5$ and $t_{0}=t-1$ , respectively since the time is measured in years.

For the error terms, we set $\epsilon_{i,1}(t)∼\text{N}(0,\sigma_{1}^{2})$ , $\epsilon_{i,2}(t)∼\text{N}(0,\sigma_{2}^{2})$ , $\epsilon_{i,3}(t)∼\text{N}(0,\sigma_{3}^{2})$ and for the random effects, we set $b_{i,1}=(b_{i0,1},b_{i1,1},b_{i2,1}{)}^{⊤}∼\text{MVN}(0,D_{1})$ **,** $b_{i,2}=(b_{i0,2},b_{i1,2},b_{i2,2},{)}^{⊤}∼\text{MVN}(0,D_{2})$ and $b_{i,3}=(b_{i0,3},b_{i1,3},b_{i2,3},{)}^{⊤}∼\text{MVN}(0,D_{3})$ . Hence, we do not assume a stacked random effects vector.

Vague priors are used for all parameters. Specifically, we assume that $\beta_{p_{1},1}∼\text{N}(0,10000)$ where $p_{1}=0,\ldots ,3$ , $\beta_{p_{2},2}∼\text{N}(0,10000)$ , $\beta_{p_{3},3}∼\text{N}(0,10000)$ where $p_{2},p_{3}=0,\ldots 5$ , $\sigma_{p_{4}}∼$ half-Cauchy(0,10) where $p_{4}=1,2,3$ , $\sigma_{b}∼$ half-Cauchy(0,10), $\alpha_{11}∼\text{N}(0,10)$ and $\alpha_{21}∼\text{N}(0,10)$ . For the correlation matrices of the random effects (let’s denote them $R_{1}$ **,** $R_{2}$ and $R_{3}$ ), we assume that both have a lkj distribution with scale parameter η=2 implying that the off-diagonal elements of the correlation matrix are near zero and reflecting the prior belief that there is no correlation between the random intercept and the random slope. We use the Euclidean Hamiltonian Monte Carlo algorithm to generate 2 chains of iterations, each containing 5000 iterations for each parameter from which the first 2500 are due to the warm-up. The target average proposal acceptance probability for adaptation δ is set to 0.99 and the maximum tree depth parameter to 20**^49–51^** (further information in the supplemental material Part B). The analysis is done with the programming language R version 4.2.2**^52^** and STAN version 2.21.0.**^53^**

### 4.2. Results

From the multivariate linear-mixed effects models considering both the current value and (total or partial) cumulative effect of the ${\text{FVC}}_{\text{up}}(\%)$ , we observe that none of the cumulative effect scenarios showed clear importance for PROMs, since the estimated coefficients for the cumulative effect are close to zero and their 95% credible intervals (Cls) include zero. In particular, for the total cumulative effect, the cumulative effect in the last six months and in the last 12 months, the estimated coefficients and their 95% Cls are 0 [0, 0.01], 0 [ − 0.11, 0.11] and − 0.01 [ − 0.23, 0.21], respectively in the model of PCS. In the model of R-PAct, they are 0.01 [0, 0.01], 0.33 [ − 0.03, 0.69] and, 0.17 [ − 0.01, 0.34], respectively. The 95% CI for the coefficient $\alpha_{21}$ includes zero, indicating that there is uncertainty about this effect. The exclusion of the cumulative effect from the models does not change the results, and the clinical interpretation becomes easier. Therefore, this association parameter was omitted from the models.

Based on the multivariate linear-mixed effects models considering only the current value of ${\text{FVC}}_{\text{up}}(\%)$ , we observe that the PCS and R-PAct are positively associated with the current value of ${\text{FVC}}_{\text{up}}(\%)$ at time t . Specifically, for a hypothetical 1% -point increase in ${\text{FVC}}_{\text{up}}(\%)$ , the PCS score is expected to be 0.14 points higher ( 95% Cl: [0.09, 0.19]) and the R-PAct 0.41 points higher [0.33, 0.49], accounting for time (i.e., when the time-point is the same), sex, age and disease duration at the start of ERT. The R-PAct is also, negatively associated with the standardized age at the start of ERT and positively associated with the sex (men having 5.7 ( 95% Cl: [2.03, 9.57]) points higher R-PAct scores than women). The PCS is not associated with the age at the start of ERT and sex (see Supplemental material, Part C, Tables C1 and C2).

**Figure 5.**
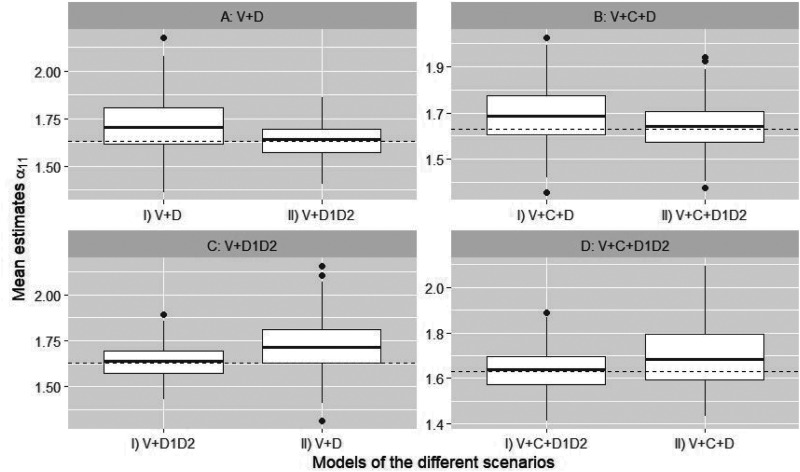
Box plots of the estimated current value of the outcome $Y_{i,1}(t)$ at time t ( $\alpha_{11}$ ) under the different scenarios presented in Table 3. At the top of each box plot the simulation model that created the data is provided. In the *x*-axis, we present the models fitted under each scenario. There is no box plot for the models of Scenario III because they do not include this parameter. Further, explanation regarding the notation used here can be found in section 5, subsection 5.4 and Table 3.

**Figure 6.**
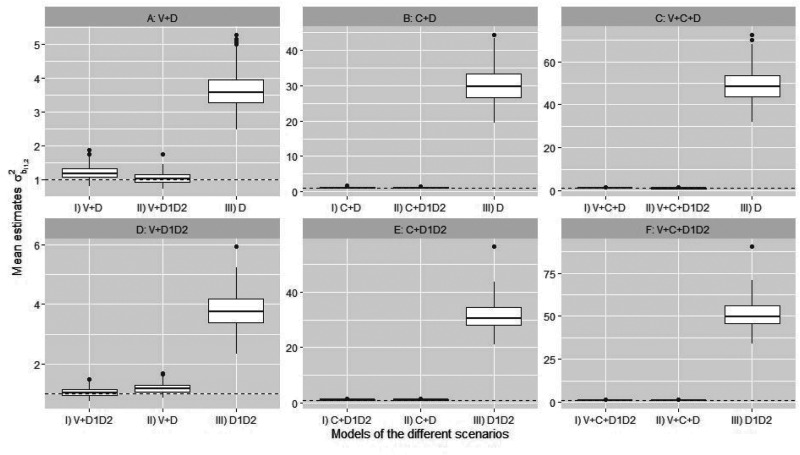
Box plots of the estimated variance of the random slope in the model of $Y_{i,2}(t)$ ( $\sigma_{b_{i12}}^{2}$ ) under the different scenarios presented in Table 3. At the top of each box plot the simulation model that created the data is provided. In the *x*-axis, we present the models fitted under each scenario. Further, explanation regarding the notation used here can be found in section 5, subsection 5.4.

The effect plots of the average patient and the trace plots are presented in the Supplemental material Part C Figures 1-14. As average patient, we define a female patient who has median standardized disease duration at start of ERT, median standardized age at start of ERT and the current value of ${\text{FVC}}_{\text{up}}(\%)$ is equal to its median estimated value. It is observed for the average patient that in the absence of any changes in ${\text{FVC}}_{\text{up}}(\%)$ , the PCS and R-PAct have a non-linear association with time, increasing in the first 5 years after starting ERT, followed by a decrease. The ${\text{FVC}}_{\text{up}}(\%)$ remains approximately stable for the first 5 years of treatment and later on starts decreasing.

## 5. Simulation Study

### 5.1. Objective

In practice, the linkage between outcomes remains unclear, often leading to ignoring their association or misspecifying the relationship of the random effects. With our simulation study, we aim to confirm firstly the unbiasedness of estimates resulting from the proposed extensions of the multivariate linear mixed-effects model, and secondly, to explore potential biases arising from disregarding additional association parameters (current value, total cumulative effect, or both) or misspecifying the association of random effects. Additionally, we aim to examine how other parameters may be affected when the association between outcomes is not appropriately addressed.

### 5.2. Simulated data

To mimic our application, we assume two continuous longitudinal outcomes $Y_{1}$ and $Y_{2}$ , with $Y_{2}$ as the primary outcome. The simulated data sets are created under different scenarios of the association structure between the two longitudinal outcomes. Specifically, we simulate data from a multivariate linear mixed-effects model with a stacked vector of random effects ( $b_{i}^{⊤}=\left(b_{i,1}^{⊤},b_{i,2}^{⊤}\right)∼\text{MVN}(0,D)$ ) and including as time-varying covariates different functional forms of the outcome $Y_{1}$ in the submodel of $Y_{2}$ : current value of $Y_{1}$ at time t (V), total cumulative effect of $Y_{1}$ until time t (C) or both of them **(V+C)**. We denote these models as V+D, C+D and V+C+D, respectively. Also, data from similar models are simulated but now it is assumed that the random effects are independent (unstacked random effects vector) so that we have $b_{i,1}^{⊤}∼\text{MVN}(0,D_{1})$ and $b_{i,2}^{⊤}∼\text{MVN}(0,D_{2})$ . These models are denoted as V+D1D2, C+D1D2, and V+C+D1D2, respectively. For each scenario, we simulate 200 data sets comprising 100 subjects, each subject with five repeated measurements.

### 5.3. Design

The extended multivariate linear mixed-effects model of the simulation study takes the form

$$\begin{array}{rl} y_{i,1}(t) & =(\beta_{0,1}+b_{i0,1})+(\beta_{1,1}+b_{i1,1})t+\beta_{2,1}{\text{sex}}_{i,1}(t)+\epsilon_{i,1}(t)=m_{i,1}(t)+\epsilon_{i,1}(t),\\ y_{i,2}(t) & =(\beta_{0,2}+b_{i0,2})+(\beta_{1,2}+b_{i1,2})t+\beta_{2,2}{\text{sex}}_{i,2}(t)+f_{1}(H_{i,1}(t),\alpha_{1},\theta_{y_{i1}})+\epsilon_{i,2}(t). \end{array}$$

where as $b_{i0,1}$ and $b_{i0,2}$ , we denote the random intercepts of the two linear mixed-effects models. The random slopes of the models are denoted as $b_{i1,1}$ and $b_{i1,2}$ , respectively. The function $f_{1}(H_{i,1}(t),\alpha_{1},\theta_{y_{i1}})$ is defined as in equation (3), (4) and (5) of the application and we set $t_{0}=0$ .

In the model of the variable $Y_{2}$ , we include the current value of $Y_{1}$ , its cumulative effect and (or) both of them. We adopt a linear effect of time for both the fixed and random parts, and we correct for sex. Time is simulated from a uniform distribution between 0 and 15, and sex is a vector that contains 0s and 1s.

The actual values of the coefficients are set to be

$${\tilde{\beta }}^{⊤}=(\beta_{1}^{⊤},\beta_{2}^{⊤})=(\beta_{0,1},\beta_{1,1},\beta_{2,1},\beta_{0,2},\beta_{1,2},\beta_{2,2})=(3.01,0.47,1.5,2.15,0.91,1.3)$$

and the actual value of the coefficient of the current value of $Y_{1}$ at time t and its cumulative effect until the time t is set to 1.63 and 0.9, respectively. For all models, the variance of the error terms and of the random effects are set equal to 1 ( $\sigma_{1}^{2}=\sigma_{2}^{2}=\sigma^{2}=1$ , $\sigma_{b_{i0,1}}^{2}=\sigma_{b_{i1,1}}^{2}=\sigma_{b_{i0,2}}^{2}=\sigma_{b_{i1,2}}^{2}=1$ ). The covariance elements of the variance–covariance matrix D of the stacked random effects vector and of the variance–covariance matrices $D_{1}$ and $D_{2}$ are set to zero. Hence, we assume the variance–covariance matrix D to be a 4×4 identity matrix and the variance–covariance matrices $D_{1}$ and $D_{2}$ to be 2×2 identity matrices. A link to the GitHub repository containing the analysis code is provided in the data availability statement.

### 5.4. Analysis

First, we fit the model using the same specifications as those that generated the simulated data set. Next, we fit models in which either the associations within the random effects are misspecified or the additional association parameters—such as current value, cumulative effect, or both—are omitted. Our primary focus is on evaluating the impact of these misspecifications on the estimations. Table 3 provides an overview of the scenarios explored. The model that generated the simulated data is referred to as the simulation model. In Scenario I, the same model is used for both simulation and fitting. In Scenario II, the association between the random effects of the simulation model is misspecified when fitting the data, and in Scenario III, the additional association parameter of the simulation model is disregarded when fitting the data. We explained the models denoted as V+D, C+D, V+C+D, V+D1D2, C+D1D2 and V+C+D1D2 in the final paragraph of the objective subsection within the simulation study section. The models denoted as D and D1D2 refer to multivariate linear mixed-effects models which do not include functional forms of $Y_{1}$ and have dependent and independent random effects, respectively.

**Table 3.** Different association structures assumed for the simulation model (model that generated the data) and the fitted model. This notation is used in figures 5 and 6.

	Fitted model		
Simulation model	Scenario I	Scenario II	Scenario III
V+D	V+D	V+D1D2	D
V+D1D2	V+D1D2	V+D	D1D2
C+D	C+D	C+D1D2	D
C+D1D2	C+D1D2	C+D	D1D2
V+C+D	V+C+D	V+C+D1D2	D
V+C+D1D2	V+C+D1D2	V+C+D	D1D2

Vague priors are used for all parameters. In particular, we assume that $\beta_{p_{1},1}∼\text{N}(0,10000)$ , $\beta_{p_{2},2}∼\text{N}(0,10000)$ , $p_{1},p_{2}=0,1,2$ , σ∼ half-Cauchy(0,10), $\sigma_{b}∼$ half-Cauchy(0,10), $\alpha_{11}∼\text{N}(0,10)$ , $\alpha_{21}∼\text{N}(0,10)$ and R∼ lkj( η=2 ). The choice of η=2 in the lkj-prior of the correlation matrix of the random effects implies that the off-diagonal elements of the correlation matrix are near zero, reflecting the prior belief that there is no correlation between the random intercept and the random slope. We use the Euclidean Hamiltonian Monte Carlo algorithm to generate 4 chains of iterations, each containing 2000 iterations for each parameter from which the first 1000 are due to the warm-up. The target average proposal acceptance probability for adaptation δ is set to 0.95 and the maximum tree depth parameter to 20. The analysis is done with the programming language R version 4.2.2**^52^** and STAN version 2.21.0.**^53^**

### 5.5. Results

Out of the 3600 generated data sets, 6.8% were excluded due to convergence issues ( $\hat{R}\geq 1.2$ ). Figures 5 and 6 illustrate the results for the association parameter ( $\alpha_{11}$ ) and the random slope variance parameter ( $\sigma_{b_{i1,2}}^{2}$ ) from the fitted models across different scenarios. For the retained data sets, we find that in Scenario I (which validates the proposed extended multivariate linear mixed-effects model), nearly all models yield unbiased estimates for all parameters, regardless of the random effects structure (whether or not a stacked random effects vector is used) or the association parameter (whether it’s based on the current value (Figure 5, panel A), total cumulative effect, or both). However, for the model V+D, there is a slight overestimation observed in the variance of the random slope ( $\sigma_{b_{i1,2}}^{2}$ ) for outcome $Y_{2}$ (Figure 6, panel A).

It is observed that all fitted models in Scenario II (where the relationship between the random effects is misspecified) yield unbiased estimates for all parameters, regardless of the inclusion of the extra association parameter in the model. However, an exception occurs when the simulation model is V+D1D2, but the model V+D is fitted instead. In this case, the variance of the random slope ( $\sigma_{b_{i1,2}}^{2}$ ) included in the model for outcome $Y_{2}$ is overestimated (Figure 6, panel D).

The models in Scenario III produce biased estimates for most parameters. Specifically, all models yield biased estimates for the coefficients and variances of the random effects included in the linear mixed-effects model for outcome $Y_{2}$ , namely the parameters $\beta_{0,2}$ , $\beta_{1,2}$ , $\beta_{2,2}$ , $\sigma_{b_{i0,2}}^{2}$ , and $\sigma_{b_{i1,2}}^{2}$ . Additionally, in Scenario III, when the models are fitted on data generated by C+D1D2 and V+C+D1D2, biased estimates are observed for the parameter $\sigma_{2}^{2}$ and the covariance between the random effects $b_{i0,2}$ and $b_{i1,2}$ . Furthermore, when the Scenario III model is fitted on data generated by V+D, biased estimates arise for the covariance between the random intercepts $b_{i0,1}$ and $b_{i0,2}$ , as well as the covariance between the random slopes $b_{i1,1}$ and $b_{i1,2}$ .

Scenario III models fitted on data generated by C+D and V+C+D yield biased estimates for most parameters. Specifically, the Scenario III model fitted on data generated by C+D results in biased estimates for all parameters except $\beta_{1,1}$ , $\sigma_{1}^{2}$ , the variance of the random intercept $b_{i0,1}$ , the covariance between $b_{i0,1}$ and $b_{i1,1}$ , the covariance between the random intercept $b_{i0,1}$ and both the random intercept $b_{i0,2}$ and the random slope $b_{i1,2}$ , as well as the covariance between the random slope $b_{i1,1}$ and both the random intercept $b_{i0,2}$ and the random slope $b_{i1,2}$ . When the Scenario III model is fitted on data generated by V+C+D, it provides the same biased estimates as the previously mentioned model, with an additional biased estimate for the covariance between the random slope $b_{i1,1}$ and both the random intercept $b_{i0,2}$ and the random slope $b_{i1,2}$ .

Overall, similar results are observed when calculating the bias and MSE values of the parameters. Notably, in the Scenario III models, the variance–covariance matrix parameters exhibit greater bias and higher MSE values compared to the other parameters discussed in this section (Supplemental material Part E, Tables 1 − 24).

### 5.6. Conclusion

In summary, we conclude that when the relationship between the random effects is incorrectly specified, the models generally produce unbiased estimates for most parameters. However, if the additional association parameter (whether current value, cumulative effect, or both) is excluded, biased estimates arise for many parameters. Specifically, the V+C+D model results in biased estimates for nearly all parameters when the additional association parameters are omitted. Therefore, it may be more prudent to consider including multiple association parameters rather than disregarding a relevant one and potentially excluding any clinically irrelevant parameters later on. Finally, incorporating a full variance–covariance matrix D along with additional association parameters may add unnecessary complexity, even when the simulation model exactly matches these specifications. Consequently, fitting the model with independent random effects might be a more practical choice.

## 6. Discussion

This study introduces an extension of the standard multivariate linear mixed-effects model within the Bayesian framework using Hamiltonian Monte Carlo. In the standard model, the random effects account for the interdependence among multiple longitudinal outcomes by assuming a multivariate normal distribution. In the current study, we assume that the primary longitudinal outcome of interest is the K- th outcome, with the other longitudinal outcomes connected to it through their current value at time t , their cumulative effect up to time t , or a combination of both. This approach aims to provide a clear and clinically meaningful interpretation of the underlying interdependence. However, the longitudinal outcomes may have additional association structures with the K -th longitudinal outcome (primary outcome), i.e., some outcomes may not be associated directly with it but through their relation with another longitudinal outcome(s) (Supplemental material Part D, Figure 15, panel A-C) or may not be associated at all, for example, the outcome $Y_{1}$ is not associated with the primary longitudinal outcome $Y_{K}$ (Supplemental material Part D, Figure 15, panel D). Moreover, there may be a bi-directional relationship between the primary outcome and other outcome(s), where both affect each other, but this is beyond the scope of the current manuscript.

Analysis of Pompe disease data using the extended model indicates that PROMs are associated only with the current value of ${\text{FVC}}_{\text{up}}(\%)$ at time t . This conclusion is drawn because the estimated coefficients for the cumulative effect across all scenarios were insignificant (with estimates close to zero and 95% credible intervals (CrIs) that included zero). Additionally, the results for the other model parameters remained largely unchanged after excluding the cumulative effect. Therefore, the inference is based on this simplified model.^4^

Based on our simulation study findings, we recommend fitting the multivariate linear mixed-effects model with various association structures, such as current values and total cumulative effects, linking the longitudinal outcomes, and subsequently eliminating association structures that are deemed less critical. This recommendation aligns with Andrinopoulou et al. (2016)^23^, who advocate for including multiple association structures in the model when uncertainty exists regarding the most appropriate association structure or when no specific structure is of particular interest. Including a full variance–covariance matrix and association parameters can introduce unnecessary complexity when the data-generating simulation model has identical and non-identical specifications (Supplemental material Part F, Tables 25 **−** 36). This observation aligns with well known challenges in fitting complex models. Misspecification, especially when complex dependency structures are assumed, can induce convergence problems or biased parameter estimates.^54^ In addition, specifying a highly complex random effects structure for each outcome further increases the risk of nonconvergence. Hence, opting for a model that assumes independent random effects may offer a more stable andpractical alternative.

The proposed extended model requires substantial computational resources, and their runtime can vary considerably depending on dataset characteristics (e.g., size, degree of imbalance, and measurement frequency), model complexity (e.g., correlation structure among random effects and number of association parameters), hardware specifications, and implementation details such as parallelization strategies. Unbalanced datasets, in particular, add complexity because they require additional steps to predict longitudinal trajectories at non-aligned time points. While these models offer flexibility and richer inference, their computational intensity may limit practical applicability for very large datasets. Future research should focus on improving scalability and efficiency, for example, by exploring alternative estimation engines (e.g., JAGS), leveraging parallel computing, and investigating dimension-reduction. Such developments will be essential to ensure that these models remain feasible for real-world applications involving high-dimensional or unbalanced data.

Also, future research could explore these extensions within the context of generalized mixed-effects models (e.g., logistic models) and Bayesian shrinkage techniques could be employed to identify the most suitable association structure for the interdependence of outcomes.

In this study, the coefficients measuring the strength of the association between longitudinal outcomes are kept constant, but outcomes are time-dependent and their associations may vary over time. For example, in Pompe disease, the clinical outcome ${\text{FVC}}_{\text{up}}(\%)$ might stabilize over time, potentially resulting in a smaller or even negative effect on the PCS/R-PAct compared to the estimated constant association parameter. Future research should therefore focus on time-varying parameters related to the outcomes. Additionally, developing and evaluating prediction models for their effectiveness is an important area for further investigation. Finally, while there is existing literature addressing the consequences of misspecifying the distribution of random effects in univariate linear and generalized linear mixed-effects models,^55–58^ this important aspect remains unexplored in the multivariate context and could be explored in the future.

## Supplemental Material

sj-pdf-1-smm-10.1177_09622802261455689 - Supplemental material for Bayesian multivariate linear mixed-effects models with varied association structures Supplemental material, sj-pdf-1-smm-10.1177_09622802261455689 for Bayesian multivariate linear mixed-effects models with varied association structures by Aglina Lika, Dimitris Rizopoulos, Michelle E Kruijshaar, Ans T van der Ploeg and Eleni-Rosalina Andrinopoulou in Statistical Methods in Medical Research
