# Supplementary material for: Bayesian multivariate linear mixed-effects models with varied association structures
Source: Stat Methods Med Res. 2026 Jun 30;35(8):1569–84. doi: 10.1177/09622802261455689 (PMC13478448; doi:10.1177/09622802261455689)
Supplement: Supplementary file 1 [file sj-pdf-1-smm-10.1177_09622802261455689.pdf]

Supplemental material: Bayesian multivariate linear mixed-effects models with varied association structures

Part A

Likelihood

If $\boldsymbol{\Theta} = (\boldsymbol{\beta}_k, \boldsymbol{\alpha}_q, \sigma_k^2, \boldsymbol{D})$, the likelihood of the model will be

$$\mathcal{L}(\boldsymbol{\Theta}) = \prod_{i=1}^{N} \mathcal{L}_i(\boldsymbol{\Theta})$$

where $\mathcal{L}_i(\boldsymbol{\Theta})$ is the contribution of the i-th individual in the likelihood of the multivariate mixed-effects model.

Let's assume that from all the submodels of the multivariate mixed effects model, we have in total V random effects. Conditioning on the random effects and considering the relationship of the dependent variables described in the main manuscript, the contribution of the i-th individual in the likelihood will be

$$\begin{aligned}
\mathcal{L}_i(\boldsymbol{\Theta}) &= \int_{\mathcal{R}^V} f(\boldsymbol{y}_{i,K} \mid H_{i,1}, \ldots, H_{i,K-1}, \boldsymbol{b_i}, \boldsymbol{\Theta}) \Big(\prod_{q=1}^{K-1} f(\boldsymbol{y}_{i,q} \mid \boldsymbol{b}_i, \boldsymbol{\Theta}) \Big) f(\boldsymbol{b}_i)\, \boldsymbol{db_i} \\
&= \int_{\mathcal{R}^V} \prod_{j=1}^{n_{i,k}} \Big[f(y_{ij,K} \mid H_{i,1}, \ldots, H_{i,K-1}, \boldsymbol{b}_i, \boldsymbol{\Theta}) \prod_{q=1}^{K-1} f(\boldsymbol{y}_{ij,q} \mid \boldsymbol{b}_i, \boldsymbol{\Theta}) f(\boldsymbol{b}_i)\, \boldsymbol{db_i} \Big] \\
&= \int_{\mathcal{R}^V} (2\pi)^{-\frac{\left(V + \sum_{k=1}^{K} n_{i,k}\right)}{2}} |\boldsymbol{D}|^{-1/2} \Big(\prod_{k=1}^{K} \sigma_k^{-1} \Big) \exp\left\{ -\frac{\boldsymbol{b_i^\tau D^{-1} b_i}}{2} \right\} \\
&\quad \times \left(\prod_{q=1}^{K-1} \exp\left\{ -\frac{[\boldsymbol{y_{i,q}} - (\boldsymbol{X_{i,q}\beta_q} + \boldsymbol{Z_{i,q} b_{i,q}})]^\tau [\boldsymbol{y_{i,q}} - (\boldsymbol{X_{i,q}\beta_q} + \boldsymbol{Z_{i,q} b_{i,q}})]}{2\sigma_k^2} \right\} \right)
\end{aligned}$$

$$\times \exp\left\{ -\frac{[\boldsymbol{y_{i,K}} - (\boldsymbol{X_{i,K}\beta_K} + \boldsymbol{Z_{i,K} b_{i,K}} + \sum_{q=1}^{K-1} f_q(H_{i,q}, \alpha_q, \boldsymbol{\theta}_{y_q}) \mathbb{1}_{\boldsymbol{n_{i,K}}})]^\tau [\boldsymbol{y_{i,K}} - (\boldsymbol{X_{i,K}\beta_K} + \boldsymbol{Z_{i,K} b_{i,K}} + \sum_{q=1}^{K-1} f_q(H_{i,q}, \alpha_q, \boldsymbol{\theta}_{y_q}) \mathbb{1}_{n_{i,K}})]}{2\sigma_K^2} \right\} \boldsymbol{db_i}$$

where $\boldsymbol{X_{i,q}}$ and $\boldsymbol{Z_{i,q}}$ are the design matrices of the fixed and random effects of the model of the q-th outcome ($q = 1, \ldots, K-1$), and $\mathbb{1}_{n_{i,K}}$ is a $n_{i,K}$-dimensional vector containing only ones; The integral does not have a closed-form solution, and thus, a numerical method must be employed for its evaluation.

Part B

Maximum tree depth parameter

The maximum tree depth parameter was set to 20 to ensure robust sampling and reliable parameter convergence. In preliminary runs with lower tree depths (e.g., 10 or 15) warnings were observed related to non-convergence, particularly for highly correlated parameters. This is expected in this complex modelling setting, where the two longitudinal outcomes are linked through both associational parameters and correlated random effects. A deeper tree allows the sampler to better explore the posterior space and reduces the risk of transitions exceeding the maximum tree depth after the warm-up phase. No major computational issues were encountered beyond increased runtime, which was considered acceptable given the improved convergence.

Part C

Estimates, effect plots of the average patient and trace plots of the application models

Table C1[1]: Estimated parameters of the multivariate mixed effects model of the PCS score with FVC in the upright position (FVC_{up}).

**Parameter**	**Estimate**	**Standard Deviation**	**2.5**%	**97.5**%	n_eff	**Rhat**
intercept_FVC	64.01	2.14	59.24	67.75	149.94	1.00
ns(t,2)1_FVC	-2.93	2.12	-7.07	1.11	372.87	1.00
ns(t,2)2_FVC	-4.42	2.90	-10.05	1.29	432.00	1.01
DisDuration_ERT	-9.47	1.88	-13.09	-5.76	246.86	1.01
intercept_PCS	26.20	2.35	21.52	30.81	1054.12	1.00
ns(t,2)1	-2.34	1.12	-4.56	-0.12	1779.08	1.00
ns(t,2)2	-7.75	1.64	-11.07	-4.59	2703.60	1.00
DisDuration_ERT_PCS	1.17	0.72	-0.23	2.60	1228.19	1.00
AgeERT	-1.11	0.68	-2.50	0.19	1179.72	1.00
Sex: female	-1.53	1.14	-3.72	0.69	1252.68	1.00
Value FVC	0.14	0.03	0.09	0.19	1325.98	1.00
sigma1	4.05	0.07	3.92	4.19	8362.81	1.00
sigma2	4.14	0.11	3.93	4.36	2079.14	1.00
D_1[1, 1]	379.36	58.10	281.88	510	2079.99	1.00
D_1[1, 2]	-17.74	40.53	-95.76	63.27	3219.16	1.00
D_1[1, 3]	-26.22	57.25	-144.33	87.47	2974.84	1.00
D_1[2, 1]	-17.74	40.53	-95.76	63.27	3219.16	1.00
D_1[2, 2]	354.51	60.73	253.59	462.89	4171.55	1.00
D_1[2, 3]	314.40	68.73	197.80	468.64	3194.39	1.00
D_1[3, 1]	-26.22	57.25	-144.33	87.47	2974.84	1.00
D_1[3, 2]	314.40	68.73	197.80	468.64	3194.39	1.00
D_1[3, 3]	560.73	117.80	365.12	823.67	2729.59	1.00
D_2[1, 1]	49.84	9.03	34.97	70.19	2016.28	1.00
D_2[1, 2]	-37.91	10.50	-61.57	-20.43	1379.78	1.00
D_2[1, 3]	-3.15	10.94	-24.28	19.78	1651.00	1.00
D_2[2, 1]	-37.91	10.50	-61.57	-20.43	1379.78	1.00
D_2[2, 2]	59.26	17.36	30.70	98.74	914.11	1.00
D_2[2, 3]	4.78	13.93	-21.99	34.52	951.09	1.01
D_2[3, 1]	-3.15	10.94	-24.28	19.78	1651.00	1.00
D_2[3, 2]	4.78	13.93	-21.99	34.52	951.00	1.01
D_2[3, 3]	62.60	38.25	8.78	153.77	181.97	1.01

[1] D_1 and D_2 denote the variance-covariance matrices of the random effects; sigma1 and sigma2 denote the variances of the error terms; ns(t,2): natural cubic splines of time with two degrees of freedom; ns(t,2)1 and ns(t,2)2 denote the estimated coefficient of the first and second component of the natural cubic splines of time with two degrees of freedom, respectively in the submodel of PCS; ns(t,2)1_FVC and ns(t,2)2_FVC denote the estimated coefficient of the first and second component of the natural cubic splines of time with two degrees of freedom, respectively in the submodel of the FVC_{up}. Here the reference category of Sex is male; n_eff: effective sample size; **2.5**% and **97.5**% are the lower and upper bound of the 95% Credible Interval, respectively.

Table C2[2]: Estimated parameters of the multivariate mixed effects model of the R-PAct score with the FVC in the upright position (FVC_{up}).

**Parameter**	**Estimate**	**Standard Deviation**	**2.5%**	**97.5%**	n_eff	**Rhat**
intercept_FVC	64.64	2.03	60.61	68.57	146.12	1.01
ns(t,2)1_FVC	-2.88	1.98	-6.80	0.98	444.83	1.00
ns(t,2)2_FVC	-4.70	2.66	-9.73	0.61	478.26	1.01
DisDuration_ERT	-8.19	1.94	-11.94	-4.37	329.69	1.00
intercept_RPACT	23.87	3.31	17.40	30.22	797.25	1.00
ns(t,2)1	-2.84	1.63	-6.08	0.35	1386.51	1.00
ns(t,2)2	-10.07	1.86	-13.76	-6.31	2270.09	1.00
DisDuration_ERT_RPACT	-1.47	1.11	-3.59	0.69	920.54	1.00
AgeERT	-3.01	0.99	-4.91	-1.08	781.61	1.00
Sex: female	5.70	1.93	2.03	9.57	781.45	1.00
Value FVC	0.41	0.04	0.33	0.49	901.96	1.00
sigma1	4.07	0.07	3.94	4.21	7040.36	1.00
sigma2	4.07	0.11	3.86	4.30	5681.50	1.00
D_1[1, 1]	385.88	63.02	282.37	529.80	3527.64	1.00
D_1[1, 2]	-15.42	41.14	-99.80	64.48	3667.70	1.00
D_1[1, 3]	-24.20	57.26	-138.56	88.72	3312.01	1.00
D_1[2, 1]	-15.42	41.14	-99.80	64.48	3667.70	1.00
D_1[2, 2]	350.50	59.96	251.13	487.67	3087.62	1.00
D_1[2, 3]	306.31	69.84	189.39	458.24	2063.51	1.00
D_1[3, 1]	-24.20	57.26	-138.56	88.72	3312.01	1.00
D_1[3, 2]	306.31	69.84	189.39	458.24	2063.51	1.00
D_1[3, 3]	543.79	118.59	351.77	808.07	1971.08	1.00
D_2[1, 1]	86.55	15.09	61.63	120.53	2877.77	1.00
D_2[1, 2]	-34.21	16.97	-70.59	-4.44	1763.79	1.00
D_2[1, 3]	1.20	16.90	-31.14	36.08	1730.05	1.00
D_2[2, 1]	-34.21	16.97	-70.59	-4.44	1763.79	1.00
D_2[2, 2]	144.20	35.14	85.75	223.69	1936.08	1.00
D_2[2, 3]	50.21	25.88	4.88	108.19	1236.19	1.00
D_2[3, 1]	1.20	16.90	-31.14	36.08	1730.05	1.00
D_2[3, 2]	50.21	25.88	4.88	108.19	1236.19	1.00
D_2[3, 3]	91.60	39.81	32.53	186.16	506.65	1.01

[2] D_1 and D_2 denote the variance-covariance matrices of the random effects; sigma1 and sigma2 denote the variances of the error terms; ns(t,2): natural cubic splines of time with two degrees of freedom; ns(t,2)1 and ns(t,2)2 denote the estimated coefficient of the first and second component of the natural cubic splines of time with two degrees of freedom, respectively in the submodel of R-PAct; ns(t,2)1_FVC and ns(t,2)2_FVC denote the estimated coefficient of the first and second component of the natural cubic splines of time with two degrees of freedom, respectively in the submodel of the FVC_{up}. Here the reference category of Sex is female; n_eff: effective sample size; **2.5**% and **97.5**% are the lower and upper bound of the 95% Credible Interval, respectively.

The effect plots of the average patient and the trace plots of the parameters of the multivariate mixed effects model of forced vital capacity in the upright position (FVC_{up}(%)) with the Physical Component summary score (PCS) and the Rasch-Built Pompe-Specific Activity (R-PAct) scale, are presented below.

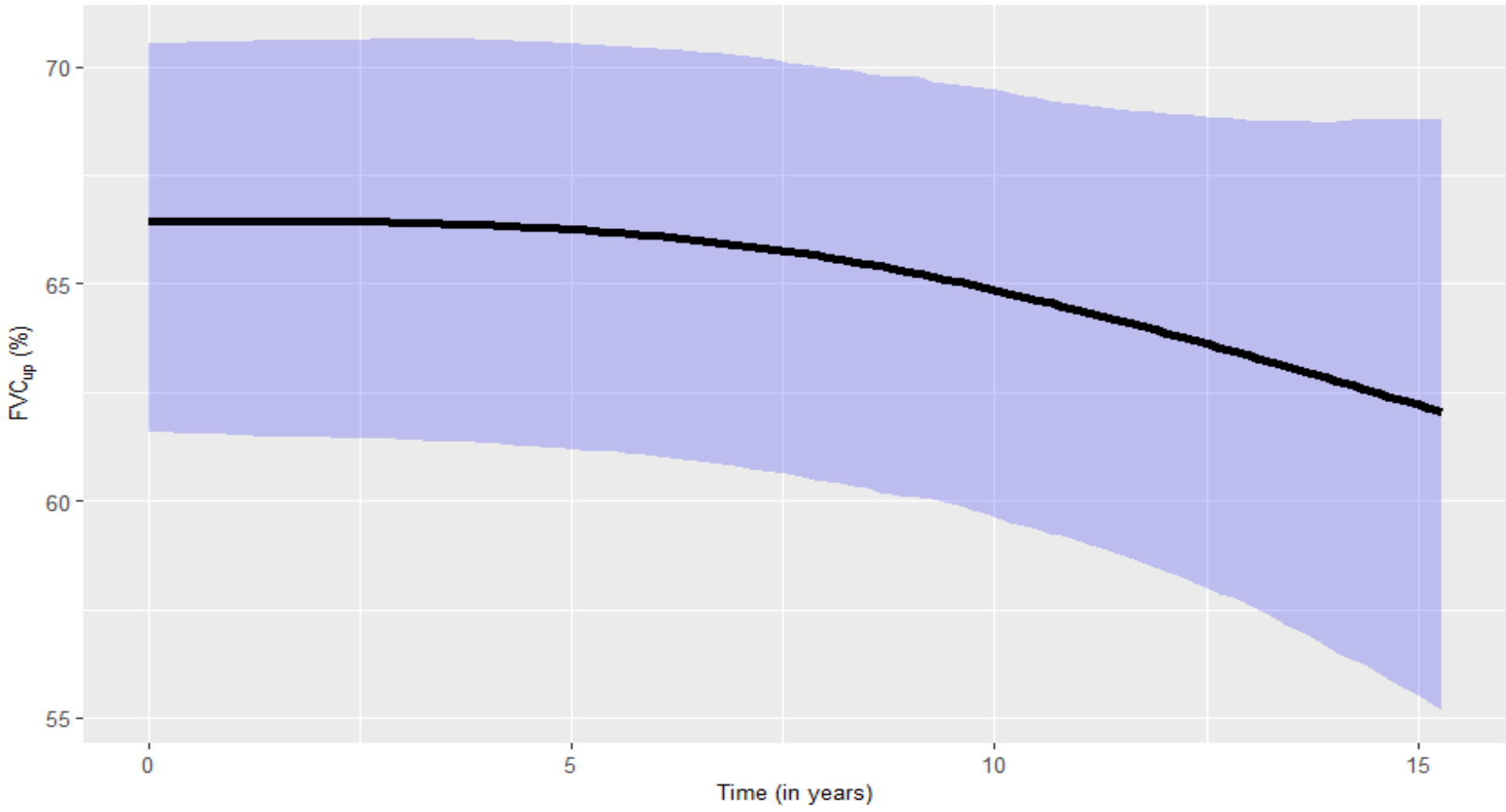

Figure 1: Effect plot of model estimates of forced vital capacity in the upright seated position (FVC_{up}(%)) by time since start of ERT for a fictitious average (i.e. a patient with median standardized disease duration at start of ERT (-0.26) which is equivalent to a disease duration of 51.39 years).

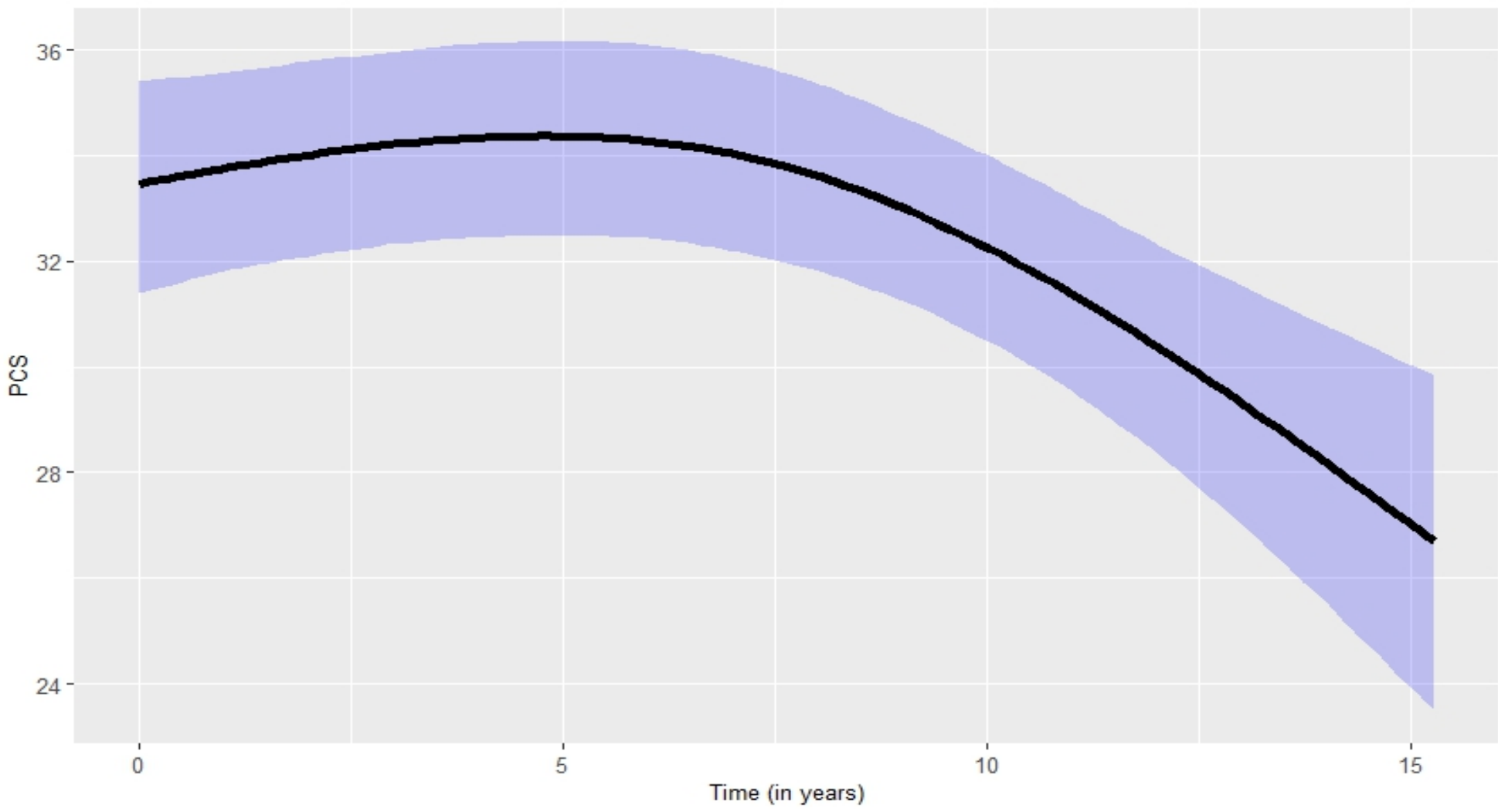

Figure 2: Effect plot of model estimates of physical component summary (PCS) score by time since start of ERT for an average patient with a constant median value of FVC_{up}(%). An average patient is a female who has median standardized disease duration at start of ERT (-0.26; equivalent to unstandardized disease duration of 51.39 years), median standardized age at start of ERT (0.1; equivalent to unstandardized age at start of ERT 13.9 years) and median estimated Forced vital capacity in upright position (FVC_{up}(%); expressed as percentage of the of the predicted normal values) measurement at a certain time-point t (65.73).

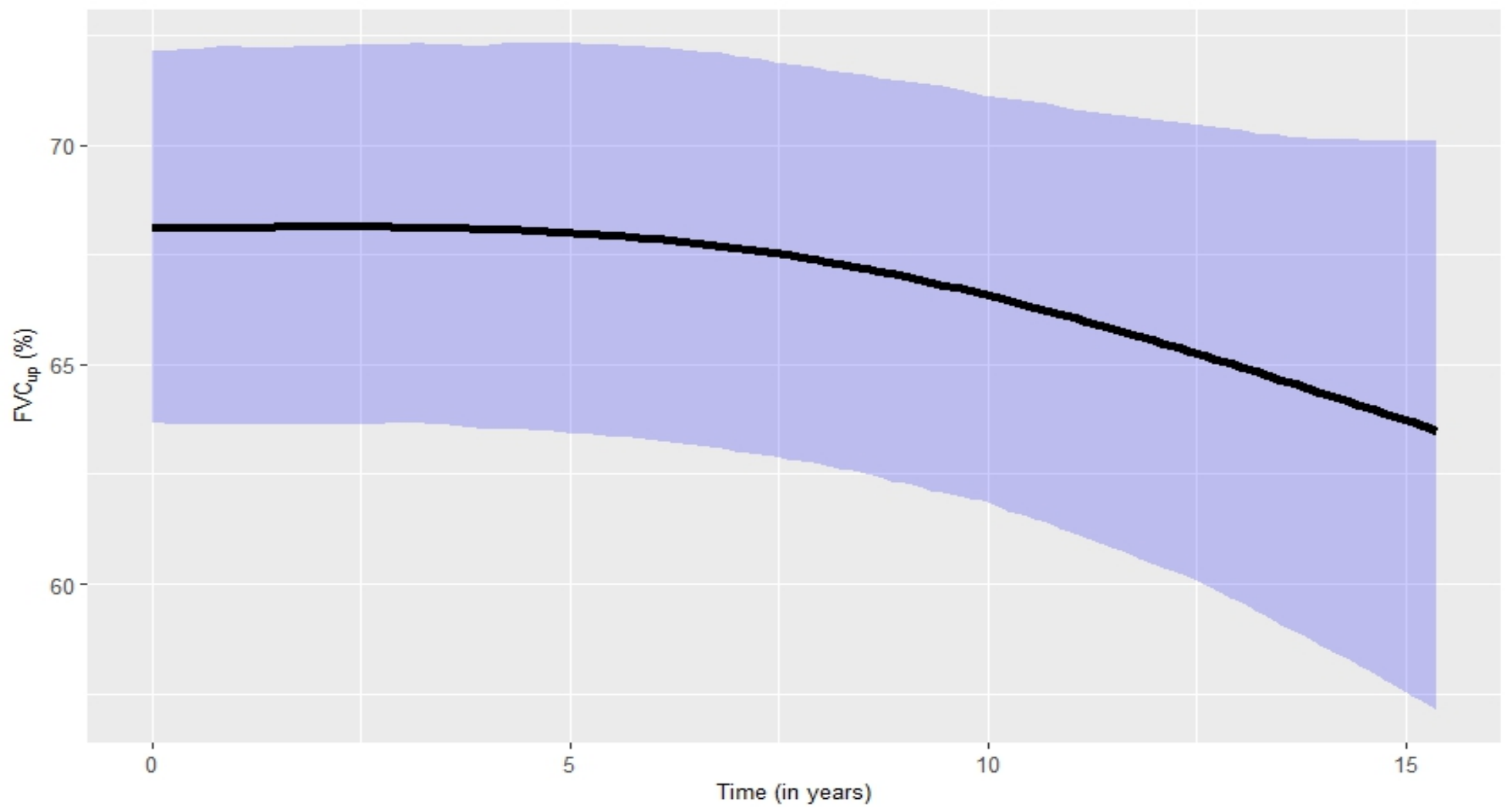

Figure 3: Effect plot of model estimates of forced vital capacity in the upright seated position (FVC_{up}(%)) by time since start of ERT for a fictitious average (i.e. a patient with median standardized disease duration at start of ERT (-0.42) which is equivalent to a disease duration of 50.5 years).

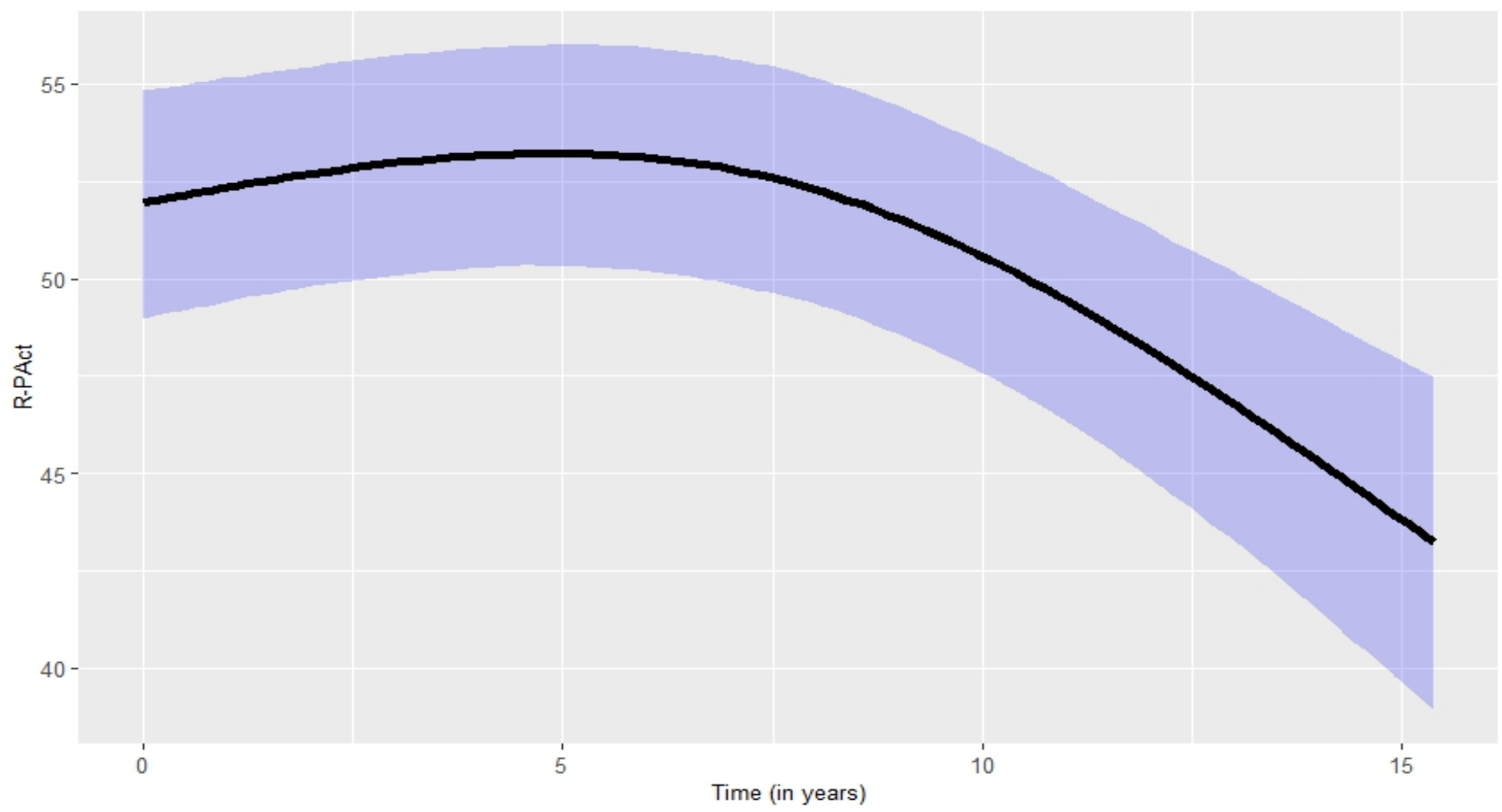

Figure 4: Effect plot of model estimates of Rasch-Built Pompe-Specific Activity (R-PAct) score by time since start of ERT for an average patient with a constant median value of FVC_{up}(%). An average patient is a female who has median standardized disease duration at start of ERT (-0.42; equivalent to unstandardized disease duration of 50.5 years), median standardized age at start of ERT (0.1; equivalent to unstandardized age at start of ERT 11.2 years) and median estimated Forced vital capacity in upright position (FVC_{up}(%); expressed as percentage of the of the predicted normal values) measurement at a certain time-point t (67.47).

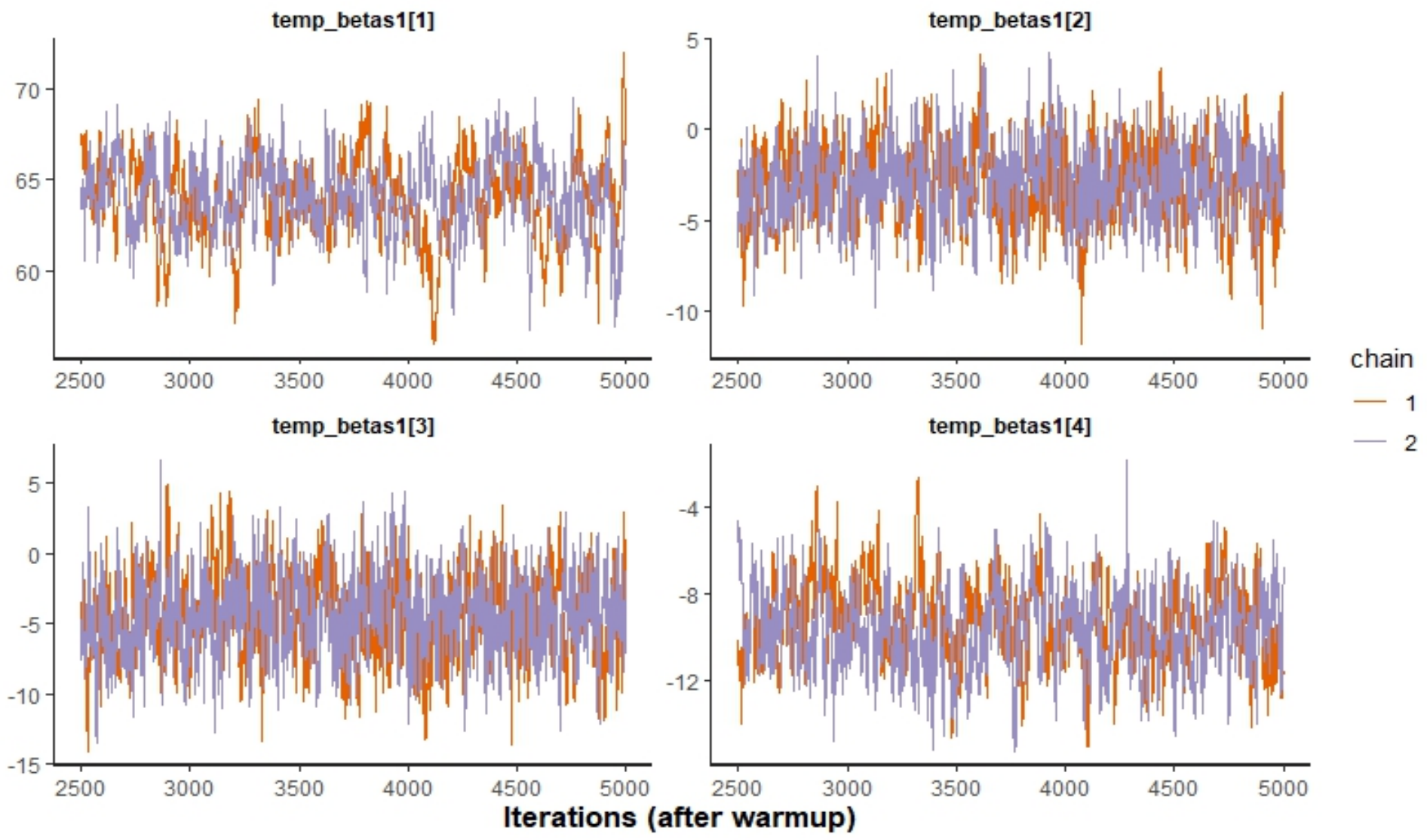

Figure 5: Trace plot of the parameters $\beta_{0,1}$, $\beta_{1,1}$, $\beta_{2,1}$ and $\beta_{3,1}$ from the sub-model for forced vital capacity in the upright position (FVC_{up}(%)) within the multivariate mixed effects model of FVC_{up}(%) with the Physical Component summary score (PCS). These parameters correspond to temp_betas1[1], temp_betas1[2], temp_betas1[3] and temp_betas1[4], respectively.

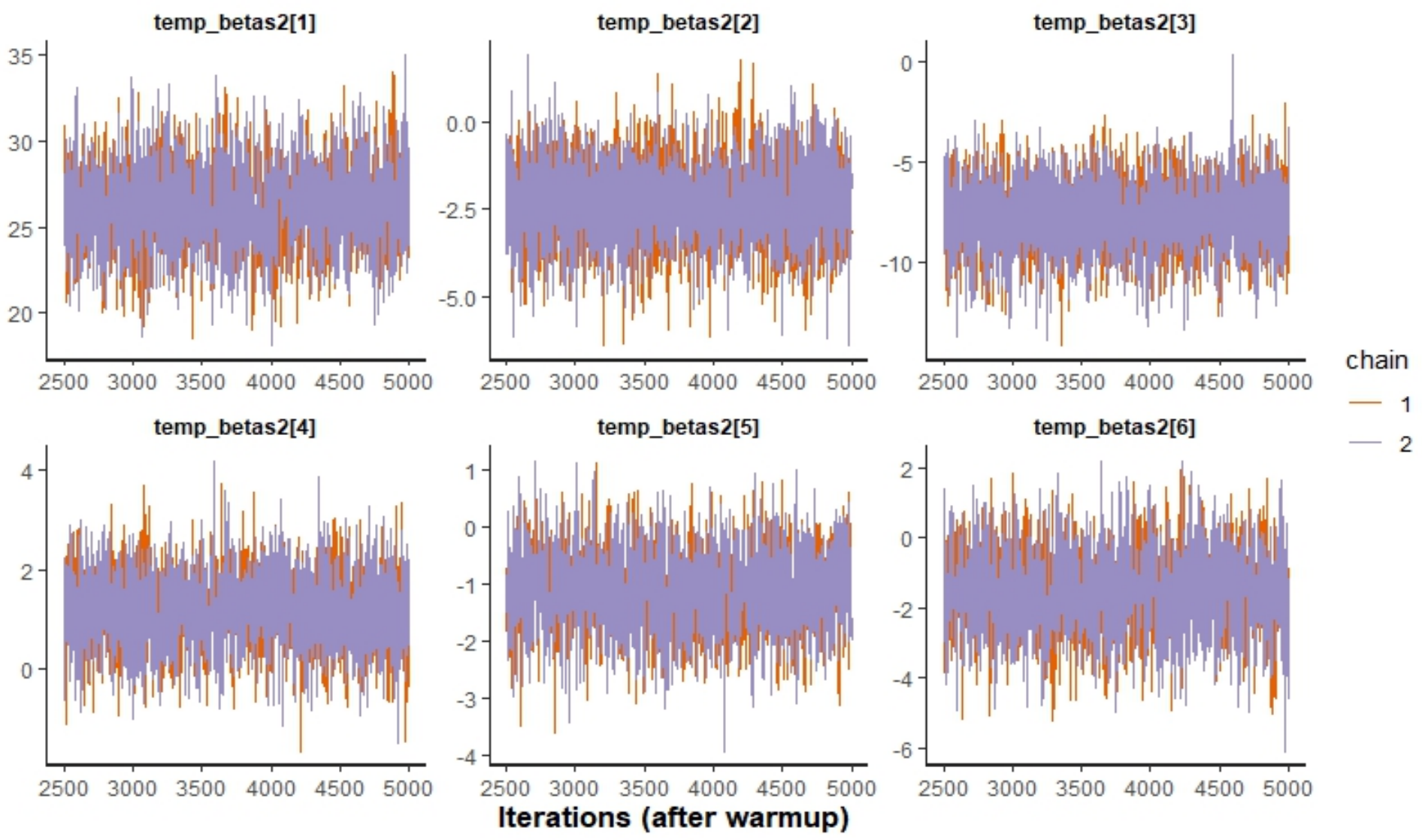

Figure 6: Trace plot of the parameters $\beta_{0,2}$, $\beta_{1,2}$, $\beta_{2,2}$, $\beta_{3,2}$, $\beta_{4,2}$ and $\beta_{5,2}$ from the sub-model of the Physical Component summary score (PCS) within the multivariate mixed effects model of FVC_{up}(%) with the PCS. These parameters are denoted as temp_betas2[1], temp_betas2[2], temp_betas2[3], temp_betas2[4], temp_betas2[5] and temp_betas2[6], respectively.

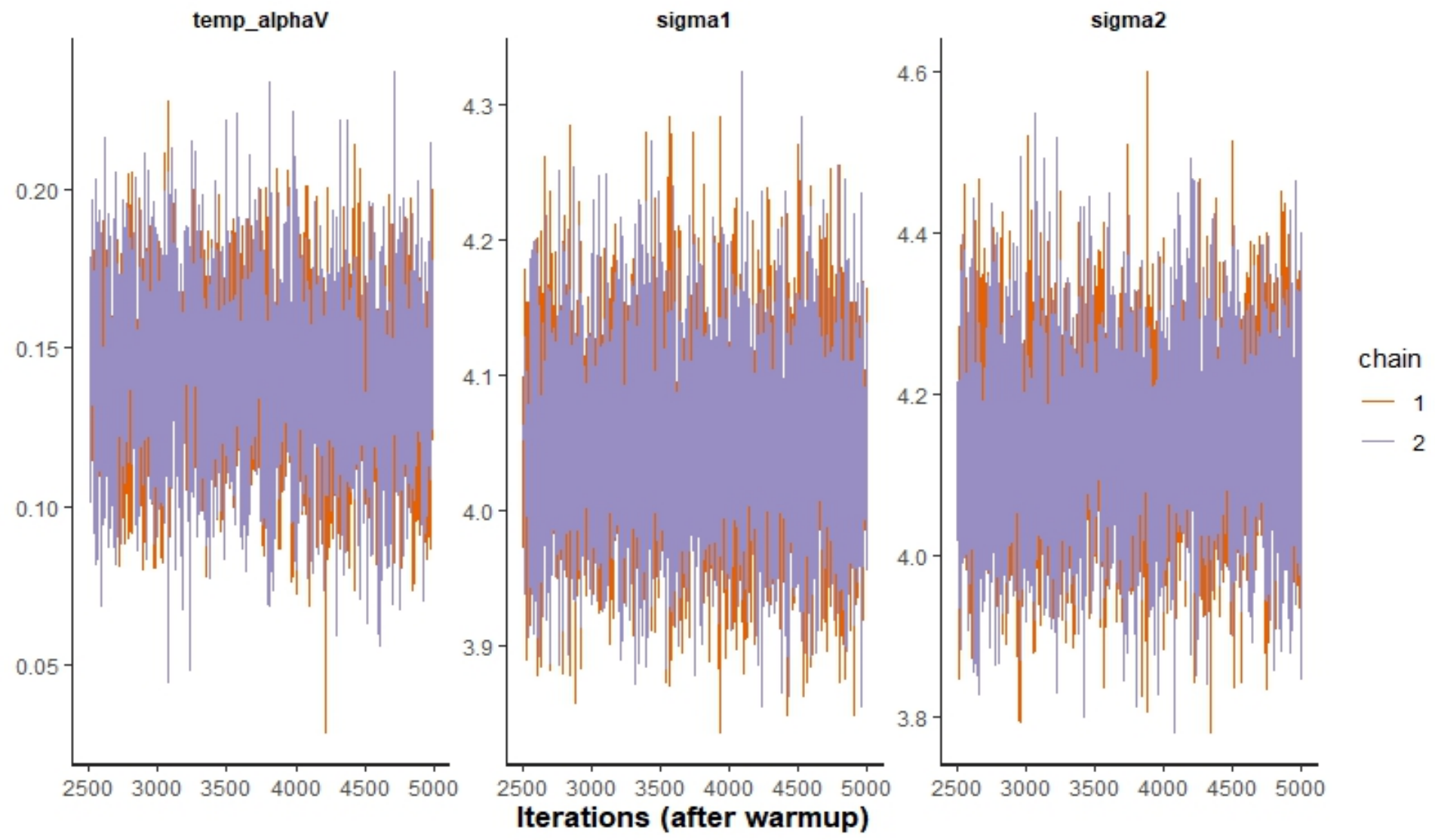

Figure 7: Trace plot of the parameters α_{11}, σ_1^2 and σ_2^2 from the multivariate mixed effects model of $\mathrm{FVC}_{\mathrm{up}}(\%)$ with the Physical Component summary score (PCS). These correspond to temp_alphaV, sigma1 and sigma2, respectively.

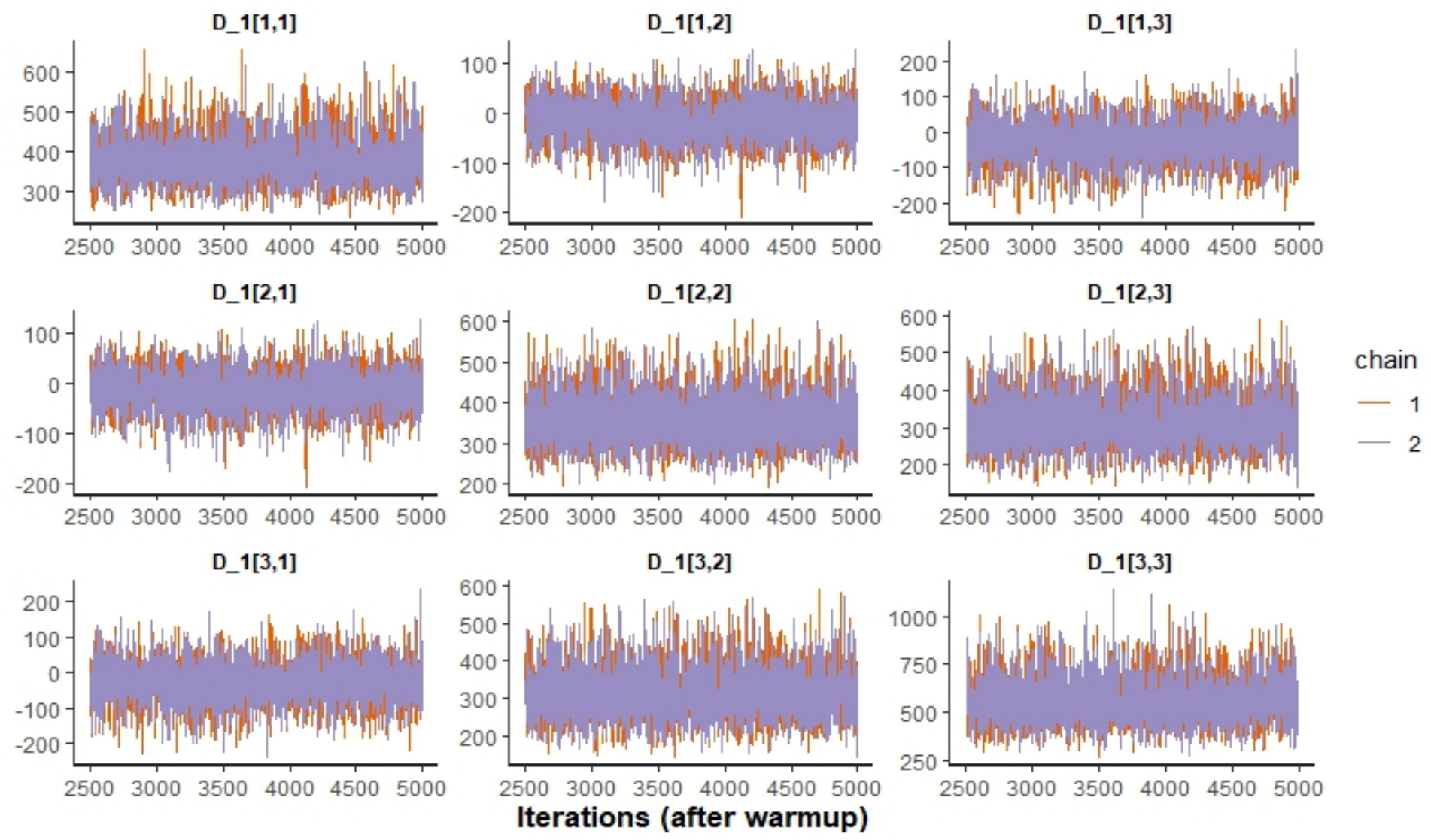

Figure 8: Trace plot of the variance-covariance parameters of the matrix D_1 of the random effects $\boldsymbol{b}_{i,1} = (b_{i0,1}, b_{i1,1}, b_{i2,1})$ from the multivariate mixed effects model of $\mathrm{FVC}_{\mathrm{up}}(\%)$ with the Physical Component summary score (PCS). The variances $\sigma^2_{b_{i0,1}}$, $\sigma^2_{b_{i1,1}}$ and $\sigma^2_{b_{i2,1}}$ correspond to D_1[1,1], D_1[2,2] and D_1[3,3], respectively. The remaining elements represent the covariances

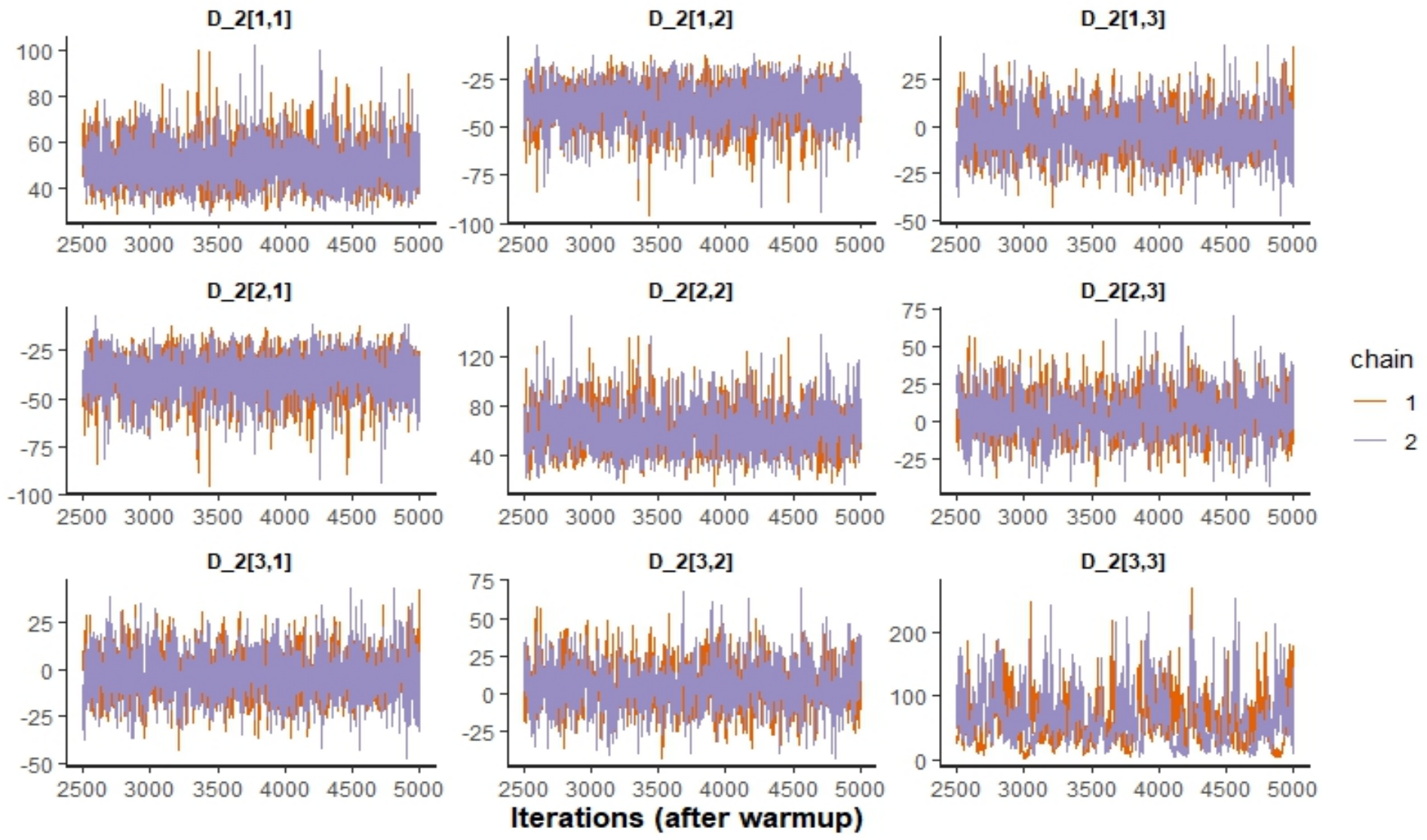

Figure 9: Trace plot of the variance-covariance parameters of the matrix D_2 of the random effects $\boldsymbol{b}_{i,2} = (b_{i0,2}, b_{i1,2}, b_{i2,2})$ from the multivariate mixed effects model of $\text{FVC}_{\text{up}}(\%)$ with the Physical Component summary score (PCS). The variances $\sigma^2_{b_{i0,2}}$, $\sigma^2_{b_{i1,2}}$ and $\sigma^2_{b_{i2,2}}$ correspond to D_2[1,1], D_2[2,2] and D_2[3,3], respectively. The remaining elements represent the covariances

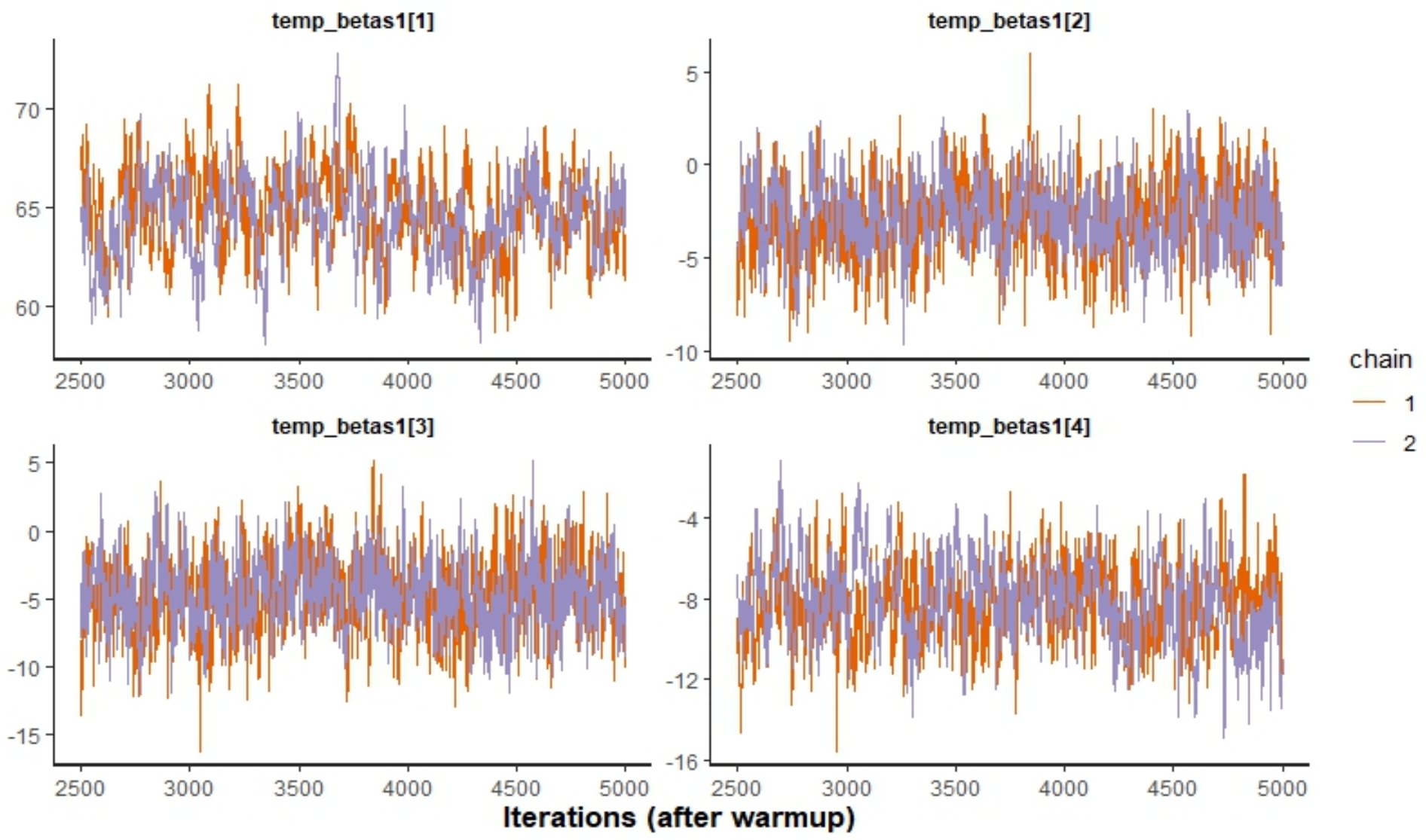

Figure 10: Trace plot of the parameters $\beta_{0,1}$, $\beta_{1,1}$, $\beta_{2,1}$ and $\beta_{3,1}$ from the sub-model of forced vital capacity in the upright position ($\text{FVC}_{\text{up}}(\%)$) within the multivariate mixed effects model of $\text{FVC}_{\text{up}}(\%)$ with the Rasch-Built Pompe-Specific Activity (R-PAct) scale. These parameters are denoted as temp_betas1[1], temp_betas1[2], temp_betas1[3] and temp_betas1[4], respectively.

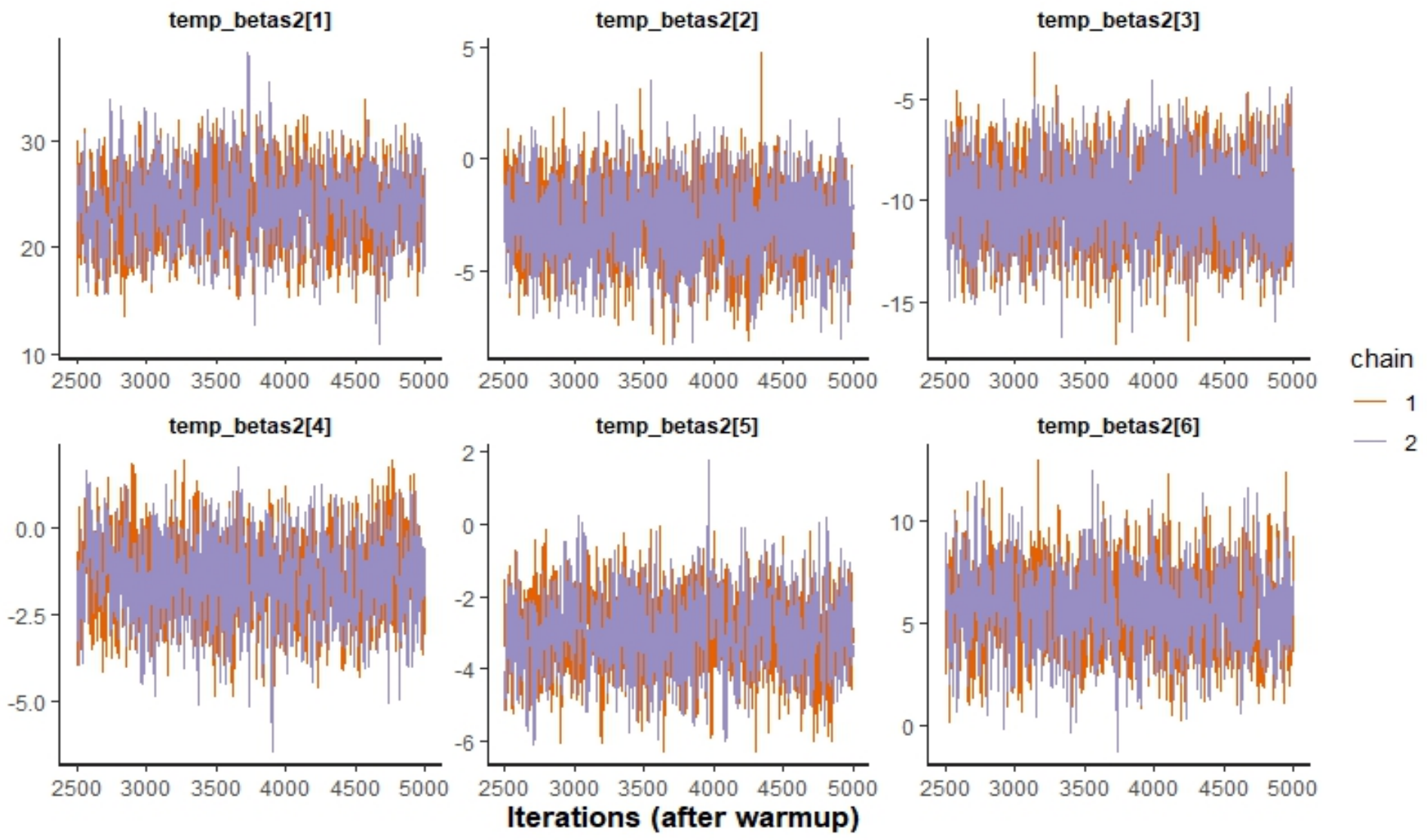

Figure 11: Trace plot of the parameters $\beta_{0,3}$, $\beta_{1,3}$, $\beta_{2,3}$, $\beta_{3,3}$, $\beta_{4,3}$ and $\beta_{5,3}$ from the sub-model of Rasch-Built Pompe-Specific Activity (R-PAct) scale within the multivariate mixed effects model of $\text{FVC}_{\text{up}}(\%)$ with the R-PAct scale. These parameters are denoted as temp_betas2[1], temp_betas2[2], temp_betas2[3], temp_betas2[4], temp_betas2[5] and temp_betas2[6], respectively.

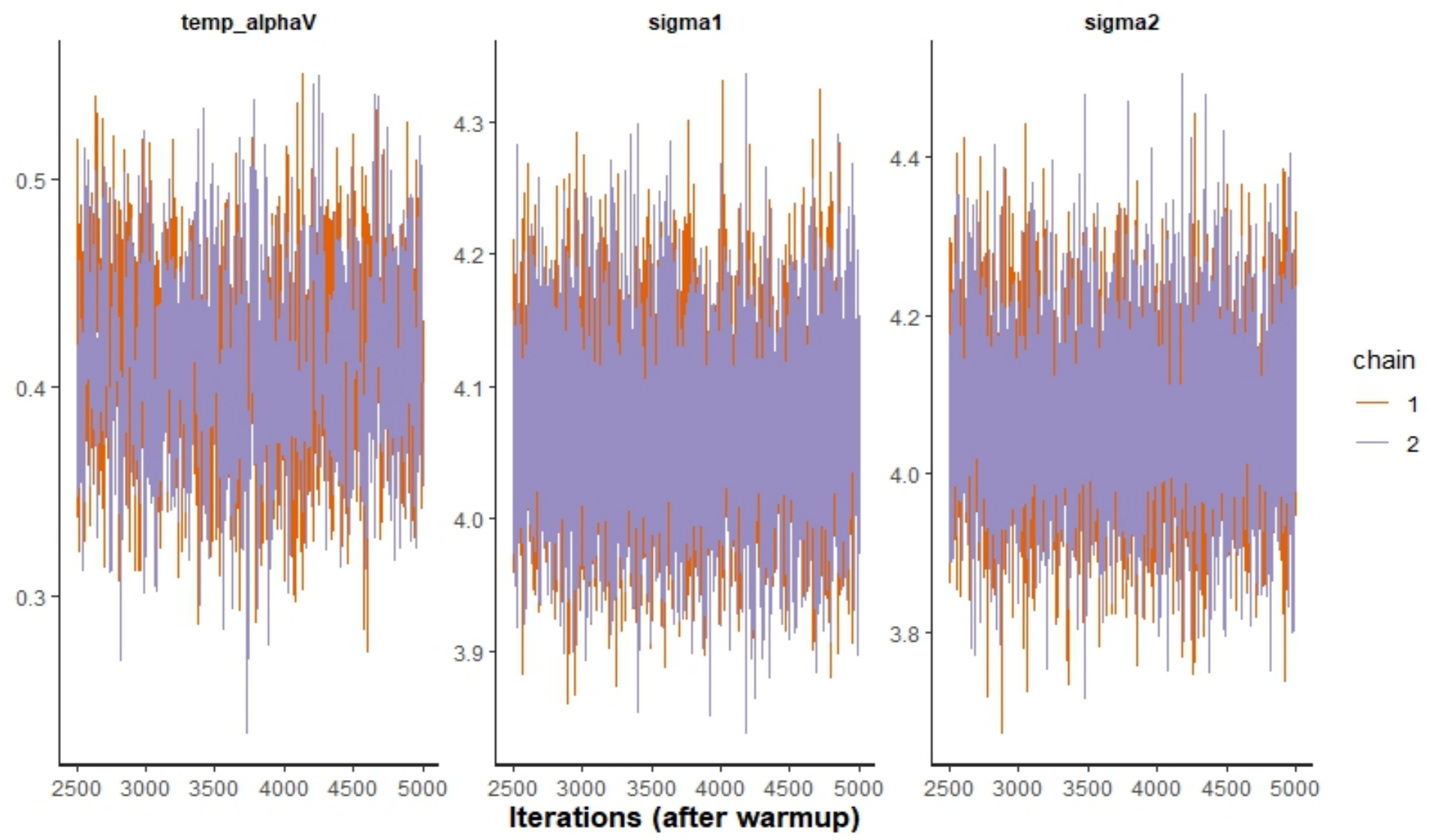

Figure 12: Trace plot of the parameters α_{11}, σ_1^2 and σ_3^2 from the multivariate mixed effects model of $\text{FVC}_{\text{up}}(\%)$ with the Rasch-Built Pompe-Specific Activity (R-PAct) scale. These parameters are denoted as temp_alphaV, sigma1 and sigma2, respectively.

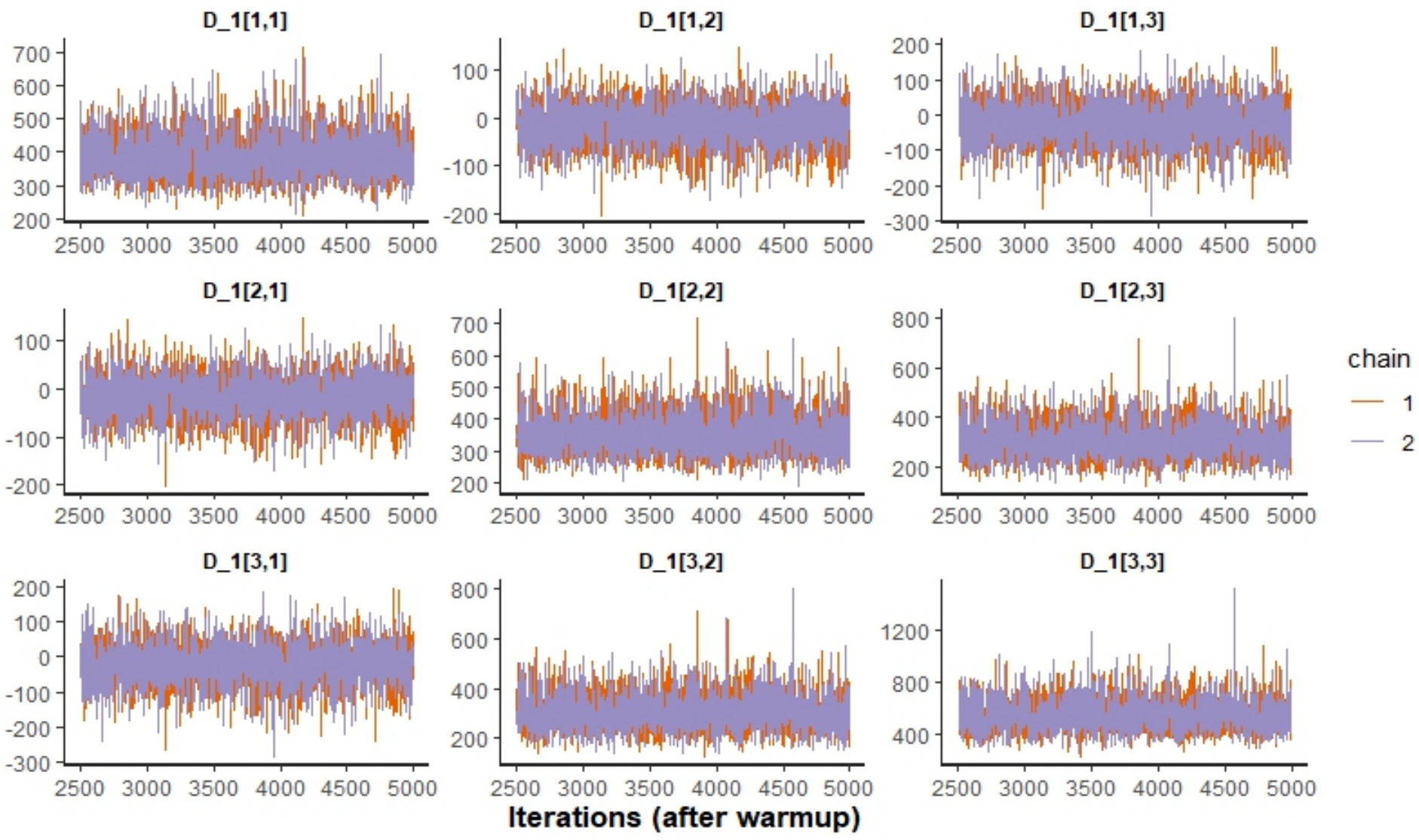

Figure 13: Trace plot of the variance-covariance parameters of the matrix D_1 of the random effects $\boldsymbol{b}_{i,1} = (b_{i0,1}, b_{i1,1}, b_{i2,1})$ from the multivariate mixed effects model of $\mathrm{FVC_{up}}(\%)$ with the Rasch-Built Pompe-Specific Activity (R-PAct) scale. The variances $\sigma^2_{b_{i0,1}}$, $\sigma^2_{b_{i1,1}}$ and $\sigma^2_{b_{i2,1}}$ correspond to D_1[1,1], D_1[2,2] and D_1[3,3], respectively. The remaining elements represent the covariances

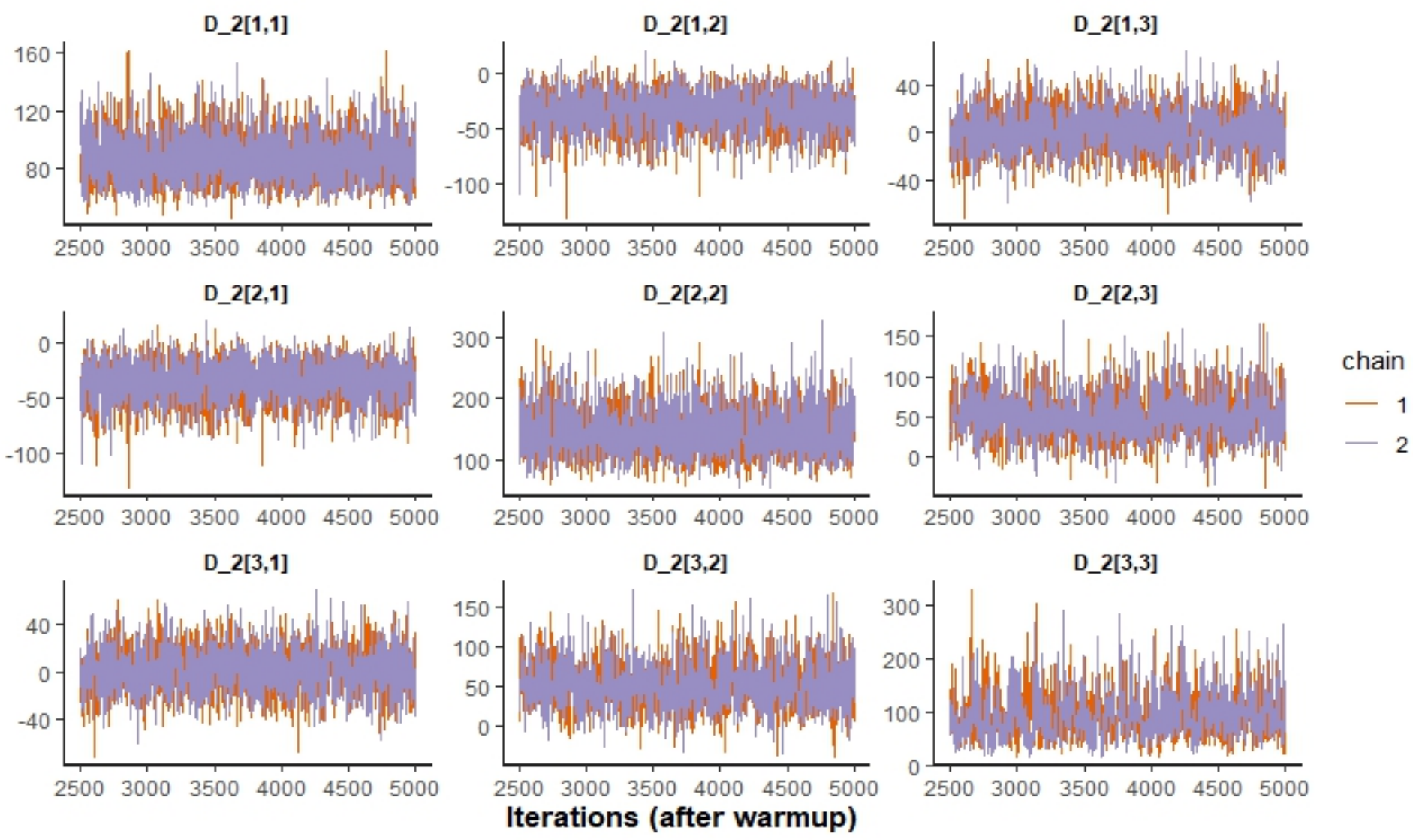

Figure 14: Trace plot of the variance-covariance parameters of the matrix D_3 of the random effects $\boldsymbol{b}_{i,3} = (b_{i0,3}, b_{i1,3}, b_{i2,3})$ from the multivariate mixed effects model of $\mathrm{FVC_{up}}(\%)$ with the Rasch-Built Pompe-Specific Activity (R-PAct) scale. The variances $\sigma^2_{b_{i0,3}}$, $\sigma^2_{b_{i1,3}}$ and $\sigma^2_{b_{i2,3}}$ correspond to D_2[1,1], D_2[2,2] and D_2[3,3], respectively. The remaining elements represent the covariances

Part D

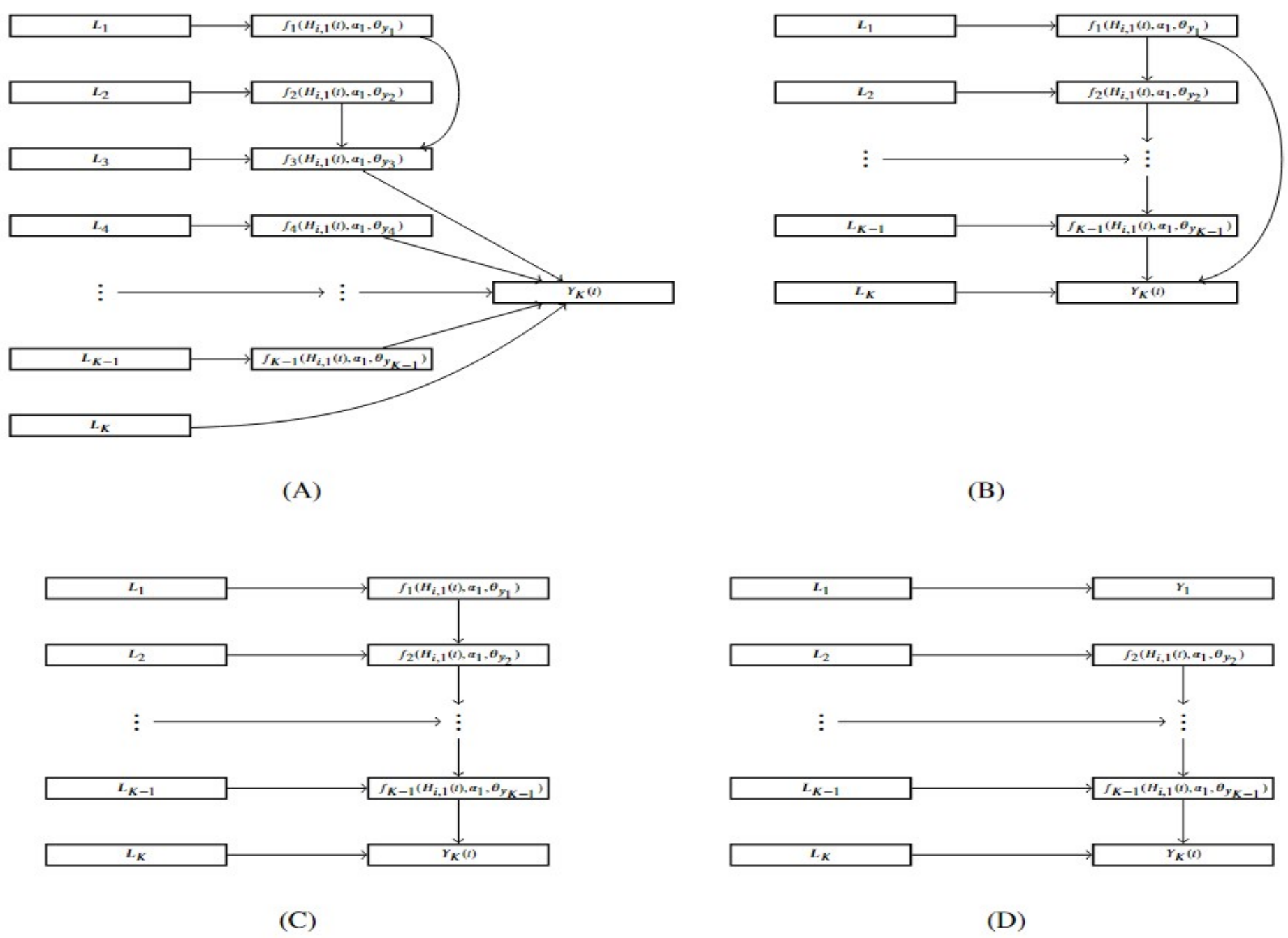

Figure 15: Four different graphs of associations between K longitudinal outcomes and K sets of confounders $(\boldsymbol{L}_1, \ldots, \boldsymbol{L}_K)$. The $f_k(\cdot)$ $(k = 1, \ldots, K-1)$ is the functional form of the Y_k longitudinal outcome that associates it with the outcome of main interest Y_K.

Part E

Simulation results

Table 1: Mean estimates, standard deviation (SD), bias and mean square error (MSE) of the fixed parameters of the fitted multivariate mixed-effects models V+D, V+D1D2 and D. While the simulation model that generated the data is the multivariate mixed-effects model V+D. The explanation of the notations used for the models and the coefficients is given in section 5, subsection 5.2.

Simulation model	Fitted model	Parameter	Real value	Estimate (SD)	Bias	MSE
V+D	V+D	$\beta_{0,1}$	3.01	3.01 (0.19)	0.00	0.04
		$\beta_{1,1}$	0.47	0.48 (0.10)	0.01	0.01
		$\beta_{2,1}$	1.50	1.49 (0.26)	-0.01	0.07
		$\beta_{0,2}$	2.15	1.87(0.54)	−0.28	0.37
		$\beta_{1,2}$	0.91	0.87 (0.12)	-0.04	0.02
		$\beta_{2,2}$	1.30	1.22 (0.43)	-0.08	0.19
		α_1	1.63	1.72(0.15)	0.09	0.03
	V+D1D2	$\beta_{0,1}$	3.01	3.01 (0.19)	0.00	0.04
		$\beta_{1,1}$	0.47	0.47 (0.10)	0.00	0.01
		$\beta_{2,1}$	1.50	1.50 (0.26)	0.00	0.07
		$\beta_{0,2}$	2.15	2.11 (0.38)	-0.05	0.15
		$\beta_{1,2}$	0.91	0.90 (0.10)	-0.01	0.01
		$\beta_{2,2}$	1.30	1.33 (0.40)	0.03	0.16
		α_1	1.63	1.64 (0.09)	0.01	0.01
	D	$\beta_{0,1}$	3.01	3.00 (0.20)	-0.01	0.04
		$\beta_{1,1}$	0.47	0.47 (0.10)	0.00	0.01
		$\beta_{2,1}$	1.50	1.51 (0.26)	0.01	0.07
		$\beta_{0,2}$	2.15	7.03(0.28)	4.88	23.89
		$\beta_{1,2}$	0.91	1.67(0.20)	0.76	0.62
		$\beta_{2,2}$	1.30	3.78(0.39)	2.48	6.31

Table 2: Mean estimates, standard deviation (SD), bias and mean square error (MSE) of the fixed parameters of the fitted multivariate mixed-effects models V+D1D2, V+D and D1D2. While the simulation model that generated the data is the multivariate mixed-effects model V+D1D2. The explanation of the notations used for the models and the coefficients is given in section 5, subsection 5.2.

Simulation model	Fitted model	Parameter	Real value	Estimate (SD)	Bias	MSE
V+D1D2	V+D1D2	$\beta_{0,1}$	3.01	3.03 (0.19)	0.01	0.04
		$\beta_{1,1}$	0.47	0.47 (0.09)	0.00	0.01
		$\beta_{2,1}$	1.50	1.47 (0.25)	-0.03	0.06
		$\beta_{0,2}$	2.15	2.11 (0.38)	-0.03	0.14
		$\beta_{1,2}$	0.91	0.90 (0.10)	-0.01	0.01
		$\beta_{2,2}$	1.30	1.29 (0.38)	-0.01	0.14
		α_1	1.63	1.64 (0.09)	0.01	0.01
	V+D	$\beta_{0,1}$	3.01	3.02 (0.19)	0.01	0.04
		$\beta_{1,1}$	0.47	0.47 (0.10)	0.00	0.01
		$\beta_{2,1}$	1.50	1.48 (0.25)	-0.02	0.06
		$\beta_{0,2}$	2.15	1.86(0.49)	−0.29	0.33
		$\beta_{1,2}$	0.91	0.86 (0.13)	-0.05	0.02
		$\beta_{2,2}$	1.30	1.16(0.42)	−0.14	0.20
		α_1	1.63	1.73(0.14)	0.10	0.03
	D1D2	$\beta_{0,1}$	3.01	3.02 (0.19)	0.01	0.04
		$\beta_{1,1}$	0.47	0.47 (0.10)	0.00	0.01
		$\beta_{2,1}$	1.50	1.47 (0.26)	-0.03	0.07
		$\beta_{0,2}$	2.15	7.06(0.30)	4.91	24.23
		$\beta_{1,2}$	0.91	1.67(0.19)	0.76	0.61
		$\beta_{2,2}$	1.30	3.71(0.41)	2.41	5.98

Table 3: Mean estimates, standard deviation (SD), bias and mean square error (MSE) of the fixed parameters of the fitted multivariate mixed-effects models C+D, C+D1D2 and D. While the simulation model that generated the data is the multivariate mixed-effects model C+D. The explanation of the notations used for the models and the coefficients is given in section 5, subsection 5.2.

Simulation model	Fitted model	Parameter	Real value	Estimate (SD)	Bias	MSE
C+D	C+D	$\beta_{0,1}$	3.01	3.01 (0.17)	0.00	0.03
		$\beta_{1,1}$	0.47	0.47 (0.10)	0.00	0.01
		$\beta_{2,1}$	1.50	1.50 (0.22)	0.00	0.05
		$\beta_{0,2}$	2.15	2.13 (0.19)	-0.02	0.04
		$\beta_{1,2}$	0.91	0.91 (0.12)	0.00	0.01
		$\beta_{2,2}$	1.30	1.33 (0.27)	0.02	0.07
		α_1	0.90	0.90 (0.01)	0.00	0.00
	C+D1D2	$\beta_{0,1}$	3.01	3.01 (0.17)	0.00	0.03
		$\beta_{1,1}$	0.47	0.47 (0.10)	0.00	0.01
		$\beta_{2,1}$	1.50	1.50 (0.22)	0.00	0.05
		$\beta_{0,2}$	2.15	2.13 (0.19)	-0.02	0.04
		$\beta_{1,2}$	0.91	0.91 (0.11)	0.00	0.01
		$\beta_{2,2}$	1.30	1.33 (0.27)	0.03	0.07
		α_1	0.90	0.90 (0.01)	0.00	0.00
	D	$\beta_{0,1}$	3.01	3.23 (0.19)	0.22	0.08
		$\beta_{1,1}$	0.47	0.46 (0.10)	0.00	0.01
		$\beta_{2,1}$	1.5	1.06(0.24)	−0.44	0.26
		$\beta_{0,2}$	2.15	−2.21(0.86)	−4.36	19.74
		$\beta_{1,2}$	0.91	6.85(0.59)	5.94	35.68
		$\beta_{2,2}$	1.3	3.63(0.78)	2.33	6.02

Table 4: Mean estimates, standard deviation (SD), bias and mean square error (MSE) of the fixed parameters of the fitted multivariate mixed-effects models C+D1D2, C+D and D1D2. While the simulation model that generated the data is the multivariate mixed-effects model C+D1D2. The explanation of the notations used for the models and the coefficients is given in section 5,subsection 5.2.

Simulation model	Fitted model	Parameter	Real value	Estimate (SD)	Bias	MSE
C+D1D2	C+D1D2	$\beta_{0,1}$	3.01	3.02 (0.17)	0.01	0.03
		$\beta_{1,1}$	0.47	0.47 (0.09)	0.00	0.01
		$\beta_{2,1}$	1.50	1.47 (0.24)	-0.03	0.06
		$\beta_{0,2}$	2.15	2.14 (0.20)	-0.01	0.04
		$\beta_{1,2}$	0.91	0.91 (0.12)	0.00	0.01
		$\beta_{2,2}$	1.30	1.29 (0.26)	-0.01	0.07
		α_1	0.90	0.90 (0.01)	0.00	0.00
	C+D	$\beta_{0,1}$	3.01	3.02 (0.17)	0.01	0.03
		$\beta_{1,1}$	0.47	0.47 (0.09)	0.00	0.01
		$\beta_{2,1}$	1.50	1.47 (0.24)	-0.03	0.06
		$\beta_{0,2}$	2.15	2.14 (0.20)	-0.01	0.04
		$\beta_{1,2}$	0.91	0.91 (0.13)	0.00	0.02
		$\beta_{2,2}$	1.30	1.29 (0.27)	-0.01	0.07
		α_1	0.90	0.90 (0.01)	0.00	0.00
	D1D2	$\beta_{0,1}$	3.01	3.02 (0.19)	0.01	0.03
		$\beta_{1,1}$	0.47	0.47 (0.09)	0.00	0.01
		$\beta_{2,1}$	1.5	1.47 (0.26)	-0.03	0.07
		$\beta_{0,2}$	2.15	$-1.72(0.81)$	-3.87	15.64
		$\beta_{1,2}$	0.91	6.80(0.53)	5.89	34.95
		$\beta_{2,2}$	1.30	2.91(0.68)	1.61	3.06

Table 5: Mean estimates, standard deviation (SD), bias and mean square error (MSE) of the fixed parameters of the fitted multivariate mixed-effects models V+C+D, V+C+D1D2 and D. While the simulation model that generated the data is the multivariate mixed-effects model V+C+D. The explanation of the notations used for the models and the coefficients is given in section 5, subsection 5.2.

Simulation model	Fitted model	Parameter	Real value	Estimate (SD)	Bias	MSE
V+C+D	V+C+D	$\beta_{0,1}$	3.01	3.02 (0.17)	0.01	0.03
		$\beta_{1,1}$	0.47	0.47 (0.10)	0.00	0.01
		$\beta_{2,1}$	1.50	1.50 (0.22)	0.00	0.05
		$\beta_{0,2}$	2.15	1.95(0.45)	-0.20	0.25
		$\beta_{1,2}$	0.91	0.88 (0.13)	-0.03	0.01
		$\beta_{2,2}$	1.30	1.24 (0.39)	-0.06	0.14
		α_{11}	1.63	1.69 (0.13)	0.06	0.02
		α_{21}	0.90	0.90 (0.01)	0.00	0.00
	V+C+D1D2	$\beta_{0,1}$	3.01	3.01 (0.17)	0.00	0.03
		$\beta_{1,1}$	0.47	0.47 (0.10)	0.00	0.01
		$\beta_{2,1}$	1.50	1.51 (0.23)	0.01	0.05
		$\beta_{0,2}$	2.15	2.10 (0.38)	-0.05	0.15
		$\beta_{1,2}$	0.91	0.91 (0.12)	0.00	0.01
		$\beta_{2,2}$	1.30	1.32 (0.38)	0.01	0.14
		α_{11}	1.63	1.64 (0.10)	0.01	0.01
		α_{21}	0.90	0.90 (0.01)	0.00	0.00
	D	$\beta_{0,1}$	3.01	3.23 (0.19)	0.22	0.08
		$\beta_{1,1}$	0.47	0.47 (0.10)	0.00	0.01
		$\beta_{2,1}$	1.5	1.04(0.23)	-0.46	0.26
		$\beta_{0,2}$	2.15	2.90(0.89)	0.75	1.35
		$\beta_{1,2}$	0.91	7.64(0.74)	6.73	45.88
		$\beta_{2,2}$	1.30	5.57(0.91)	4.27	19.08

Table 6: Mean estimates, standard deviation (SD), bias and mean square error (MSE) of the fixed parameters of the fitted multivariate mixed-effects models V+C+D1D2, V+C+D and D1D2. While the simulation model that generated the data is the multivariate mixed-effects model V+C+D1D2. The explanation of the notations used for the models and the coefficients is given in section 5, subsection 5.2.

Simulation model	Fitted model	Parameter	Real value	Estimate (SD)	Bias	MSE
V+C+D1D2	V+C+D1D2	$\beta_{0,1}$	3.01	3.03 (0.17)	0.02	0.03
		$\beta_{1,1}$	0.47	0.47 (0.10)	0.00	0.01
		$\beta_{2,1}$	1.50	1.47 (0.22)	-0.03	0.05
		$\beta_{0,2}$	2.15	2.11 (0.45)	-0.04	0.14
		$\beta_{1,2}$	0.91	0.90 (0.13)	-0.01	0.02
		$\beta_{2,2}$	1.30	1.30 (0.39)	-0.01	0.13
		α_{11}	1.63	1.64 (0.09)	0.01	0.01
		α_{21}	0.90	0.90 (0.01)	0.00	0.00
	V+C+D	$\beta_{0,1}$	3.01	3.01 (0.17)	0.00	0.03
		$\beta_{1,1}$	0.47	0.47 (0.10)	0.00	0.01
		$\beta_{2,1}$	1.50	1.49 (0.24)	-0.01	0.06
		$\beta_{0,2}$	2.15	1.91(0.46)	−0.24	0.27
		$\beta_{1,2}$	0.91	0.88 (0.13)	-0.03	0.02
		$\beta_{2,2}$	1.30	1.20(0.40)	−0.10	0.17
		α_{11}	1.63	1.70 (0.13)	0.07	0.02
		α_{21}	0.90	0.90 (0.01)	0.00	0.00
	D1D2	$\beta_{0,1}$	3.01	3.02 (0.19)	0.01	0.04
		$\beta_{1,1}$	0.47	0.47 (0.09)	0.00	0.01
		$\beta_{2,1}$	1.50	1.47 (0.26)	-0.04	0.07
		$\beta_{0,2}$	2.15	3.38(0.85)	1.23	2.23
		$\beta_{1,2}$	0.91	7.57(0.68)	6.66	44.81
		$\beta_{2,2}$	1.30	4.93(0.84)	3.63	13.88

Table 7: Mean estimates, standard deviation (SD), bias and mean square error (MSE) of the variance parameters of the error terms $\epsilon_{i,1}$ and $\epsilon_{i,2}$ (denoted as σ_1^2 and σ_2^2, respectively) and of the parameters of the variance-covariance matrix $\boldsymbol{D}$ of the random effects of the fitted multivariate mixed-effects model V+D. While the simulation model that generated the data is the multivariate mixed-effects model V+D. We denote as σ_{11}^2, σ_{22}^2, σ_{33}^2 and σ_{44}^2 the variance of the random effect $b_{i0,1}$, $b_{i1,1}$, $b_{i0,2}$ and $b_{i1,2}$, respectively. As σ_{12}, σ_{13}, σ_{14}, we denote the covariance of the random intercept $b_{i0,1}$ with the random effect $b_{i1,1}$, $b_{i0,2}$ and $b_{i1,2}$, respectively, and as σ_{23}, σ_{24}, we denote the covariance of the random slope $b_{i1,1}$ with the random effect $b_{i0,2}$ and $b_{i1,2}$, respectively. We denote as σ_{34} the covariance of the random intercept $b_{i0,2}$ with the random slope $b_{i1,2}$. The models are explained in section 5, subsection 5.2 and presented in Table 3 of subsection 5.4.

Simulation model	Fitted model	Parameter	Real value	Estimate (SD)	Bias	MSE
V+D	V+D	σ_1^2	1.00	1.01 (0.04)	0.01	0.00
		σ_2^2	1.00	1.01 (0.04)	0.01	0.00
		σ_{11}^2	1.00	1.04 (0.22)	0.04	0.05
		σ_{12}	0.00	0.00 (0.10)	0.00	0.01
		σ_{13}	0.00	-0.01 (0.10)	0.00	0.01
		σ_{14}	0.00	-0.01 (0.12)	-0.01	0.01
		σ_{22}^2	1.00	1.04 (0.15)	0.04	0.02
		σ_{23}	0.00	-0.01 (0.13)	-0.01	0.02
		σ_{24}	0.00	-0.08 (0.14)	-0.08	0.03
		σ_{33}^2	1.00	0.98 (0.49)	-0.02	0.24
		σ_{34}	0.00	0.01 (0.15)	0.01	0.02
		σ_{44}^2	1.00	1.20(0.19)	0.20	0.07

Table 8: Mean estimates, standard deviation (SD), bias and mean square error (MSE) of the variance parameters of the error terms $\epsilon_{i,1}$ and $\epsilon_{i,2}$ (denoted as σ_1^2 and σ_2^2, respectively) and of the parameters of the variance-covariance matrices $\boldsymbol{D_1}$ and $\boldsymbol{D_2}$ of the random effects of the fitted multivariate mixed-effects model V+D1D2. While the simulation model that generated the data is the multivariate mixed-effects model V+D. We denote as $\sigma_{11,1}^2$, $\sigma_{22,1}^2$, $\sigma_{11,2}^2$ and $\sigma_{22,2}^2$ the variance of the random effect $b_{i0,1}$, $b_{i1,1}$, $b_{i0,2}$ and $b_{i1,2}$, respectively. As $\sigma_{12,1}$, we denote the covariance of the random intercept $b_{i0,1}$ with the random slope $b_{i1,1}$ and as $\sigma_{12,2}$ the covariance of the random intercept $b_{i0,2}$ with the random slope $b_{i1,2}$. The models are explained in section 5, subsection 5.2 and presented in Table 3 of subsection 5.4.

Simulation model	Fitted model	Parameter	Real value	Estimate (SD)	Bias	MSE
V+D	V+D1D2	σ_1^2	1.00	1.01 (0.04)	0.01	0.00
		σ_2^2	1.00	1.01 (0.04)	0.01	0.00
		$\sigma_{11,1}^2$	1.00	1.05 (0.19)	0.05	0.04
		$\sigma_{12,1}$	0.00	0.00 (0.10)	0.00	0.01
		$\sigma_{22,1}^2$	1.00	1.03 (0.15)	0.03	0.02
		$\sigma_{11,2}^2$	1.00	1.01 (0.42)	0.01	0.18
		$\sigma_{12,2}$	0.00	-0.01 (0.16)	-0.01	0.03
		$\sigma_{22,2}^2$	1.00	1.05 (0.16)	0.05	0.03

Table 9: Mean estimates, standard deviation (SD), bias and mean square error (MSE) of the variance parameters of the error terms $\epsilon_{i,1}$ and $\epsilon_{i,2}$ (denoted as σ_1^2 and σ_2^2, respectively) and of the parameters of the variance-covariance matrix $\boldsymbol{D}$ of the random effects of the fitted multivariate mixed-effect models D. While the simulation model that generated the data is the multivariate mixed-effects model V+D. We denote as σ_{11}^2, σ_{22}^2, σ_{33}^2 and σ_{44}^2 the variance of the random effect $b_{i0,1}$, $b_{i1,1}$, $b_{i0,2}$ and $b_{i1,2}$, respectively. As σ_{12}, σ_{13}, σ_{14}, we denote the covariance of the random intercept $b_{i0,1}$ with the random effect $b_{i1,1}$, $b_{i0,2}$ and $b_{i1,2}$, respectively, and as σ_{23}, σ_{24}, we denote the covariance of the random slope $b_{i1,1}$ with the random effect $b_{i0,2}$ and $b_{i1,2}$, respectively. We denote as σ_{34} the covariance of the random intercept $b_{i0,2}$ with the random slope $b_{i1,2}$. The models are explained in section 5, subsection 5.2 and presented in Table 3 of subsection 5.4.

Simulation model	Fitted model	Parameter	Real value	Estimate (SD)	Bias	MSE
V+D	D	σ_1^2	1.00	1.00 (0.04)	0.00	0.00
		σ_2^2	1.00	1.00 (0.04)	0.00	0.00
		σ_{11}^2	1.00	1.04 (0.22)	0.04	0.05
		σ_{12}	0.00	-0.01 (0.10)	-0.01	0.01
		σ_{13}	0.00	1.53(0.29)	1.53	2.43
		σ_{14}	0.00	-0.01 (0.19)	-0.01	0.04
		σ_{22}^2	1.00	1.01 (0.14)	0.01	0.02
		σ_{23}	0.00	0.00 (0.17)	0.00	0.03
		σ_{24}	0.00	1.59(0.25)	1.59	2.61
		σ_{33}^2	1.00	3.71(0.60)	2.71	7.70
		σ_{34}	0.00	-0.05 (0.32)	-0.05	0.10
		σ_{44}^2	1.00	3.66(0.55)	2.66	7.36

Table 10: Mean estimates, standard deviation (SD), bias and mean square error (MSE) of the variance parameters of the error terms $\epsilon_{i,1}$ and $\epsilon_{i,2}$ (denoted as σ_1^2 and σ_2^2, respectively) and of the parameters of the variance-covariance matrices $\boldsymbol{D_1}$ and $\boldsymbol{D_2}$ of the random effects of the fitted multivariate mixed-effects model V+D1D2. While the simulation model that generated the data is the multivariate mixed-effects model V+D1D2. We denote as $\sigma_{11,1}^2$, $\sigma_{22,1}^2$, $\sigma_{11,2}^2$ and $\sigma_{22,2}^2$ the variance of the random effect $b_{i0,1}$, $b_{i1,1}$, $b_{i0,2}$ and $b_{i1,2}$, respectively. As $\sigma_{12,1}$, we denote the covariance of the random intercept $b_{i0,1}$ with the random slope $b_{i1,1}$ and as $\sigma_{12,2}$ the covariance of the random intercept $b_{i0,2}$ with the random slope $b_{i1,2}$. The models are explained in section 5, subsection 5.2 and presented in Table 3 of subsection 5.4.

Simulation model	Fitted model	Parameter	Real value	Estimate (SD)	Bias	MSE
V+D1D2	V+D1D2	σ_1^2	1.00	1.01 (0.04)	0.01	0.00
		σ_2^2	1.00	1.01 (0.04)	0.01	0.00
		$\sigma_{11,1}^2$	1.00	1.05 (0.20)	0.05	0.04
		$\sigma_{12,1}$	0.00	0.00 (0.11)	0.00	0.01
		$\sigma_{22,1}^2$	1.00	1.05 (0.16)	0.05	0.02
		$\sigma_{11,2}^2$	1.00	0.99 (0.42)	0.06	0.24
		$\sigma_{12,2}$	0.00	-0.01 (0.15)	0.00	0.02
		$\sigma_{22,2}^2$	1.00	1.19 (0.15)	0.04	0.07

Table 11: Mean estimates, standard deviation (SD), bias and mean square error (MSE) of the variance parameters of the error terms $\epsilon_{i,1}$ and $\epsilon_{i,2}$ (denoted as σ_1^2 and σ_2^2, respectively) and of the parameters of the variance-covariance matrix $\boldsymbol{D}$ of the random effects of the fitted multivariate mixed-effect models V+D. While the simulation model that generated the data is the multivariate mixed-effects model V+D1D2. We denote as σ_{11}^2, σ_{22}^2, σ_{33}^2 and σ_{44}^2 the variance of the random effect $b_{i0,1}$, $b_{i1,1}$, $b_{i0,2}$ and $b_{i1,2}$, respectively. As σ_{12}, σ_{13}, σ_{14}, we denote the covariance of the random intercept $b_{i0,1}$ with the random effect $b_{i1,1}$, $b_{i0,2}$ and $b_{i1,2}$, respectively, and as σ_{23}, σ_{24}, we denote the covariance of the random slope $b_{i1,1}$ with the random effect $b_{i0,2}$ and $b_{i1,2}$, respectively. We denote as σ_{34} the covariance of the random intercept $b_{i0,2}$ with the random slope $b_{i1,2}$. The models are explained in section 5, subsection 5.2 and presented in Table 3 of subsection 5.4.

Simulation model	Fitted model	Parameter	Real value	Estimate (SD)	Bias	MSE
V+D1D2	V+D	σ_1^2	1.00	1.01 (0.04)	0.01	0.00
		σ_2^2	1.00	1.01 (0.04)	0.01	0.00
		σ_{11}^2	1.00	1.03 (0.23)	0.03	0.05
		σ_{12}	0.00	0.00 (0.11)	0.00	0.01
		σ_{13}	0.00	0.00 (0.11)	0.00	0.01
		σ_{14}	0.00	0.00 (0.12)	0.00	0.01
		σ_{22}^2	1.00	1.07 (0.16)	0.07	0.03
		σ_{23}	0.00	-0.01 (0.16)	-0.01	0.02
		σ_{24}	0.00	-0.09 (0.15)	-0.10	0.03
		σ_{33}^2	1.00	1.02 (0.50)	0.02	0.25
		σ_{34}	0.00	0.01 (0.14)	0.01	0.02
		σ_{44}^2	1.00	1.20(0.17)	0.20	0.07

Table 12: Mean estimates, standard deviation (SD), bias and mean square error (MSE) of the variance parameters of the error terms $\epsilon_{i,1}$ and $\epsilon_{i,2}$ (denoted as σ_1^2 and σ_2^2, respectively) and of the parameters of the variance-covariance matrices $\boldsymbol{D_1}$ and $\boldsymbol{D_2}$ of the random effects of the fitted multivariate mixed-effects model D1D2. While the simulation model that generated the data is the multivariate mixed-effects model V+D1D2. We denote as $\sigma^2_{11,1}$, $\sigma^2_{22,1}$, $\sigma^2_{11,2}$ and $\sigma^2_{22,2}$ the variance of the random effect $b_{i0,1}$, $b_{i1,1}$, $b_{i0,2}$ and $b_{i1,2}$, respectively. As $\sigma_{12,1}$, we denote the covariance of the random intercept $b_{i0,1}$ with the random slope $b_{i1,1}$ and as $\sigma_{12,2}$ the covariance of the random intercept $b_{i0,2}$ with the random slope $b_{i1,2}$. The models are explained in section 5, subsection 5.2 and presented in Table 3 of subsection 5.4.

Simulation model	Fitted model	Parameter	Real value	Estimate (SD)	Bias	MSE
V+D1D2	D1D2	σ_1^2	1.00	1.01 (0.04)	0.01	0.00
		σ_2^2	1.00	1.01 (0.04)	0.01	0.00
		$\sigma^2_{11,1}$	1.00	1.03 (0.23)	0.03	0.05
		$\sigma_{12,1}$	0.00	0.00 (0.12)	0.00	0.01
		$\sigma^2_{22,1}$	1.00	1.05 (0.16)	0.05	0.03
		$\sigma^2_{11,2}$	0.00	3.84(0.64)	2.84	8.49
		$\sigma_{12,2}$	0.00	0.00 (0.40)	0.00	0.16
		$\sigma^2_{22,2}$	1.00	3.81(0.60)	2.81	8.24

Table 13: Mean estimates, standard deviation (SD), bias and mean square error (MSE) of the variance parameters of the error terms $\epsilon_{i,1}$ and $\epsilon_{i,2}$ (denoted as σ_1^2 and σ_2^2, respectively) and of the parameters of the variance-covariance matrix $\boldsymbol{D}$ of the random effects of the fitted multivariate mixed-effect models C+D. While the simulation model that generated the data is the multivariate mixed-effects model C+D. We denote as σ^2_{11}, σ^2_{22}, σ^2_{33} and σ^2_{44} the variance of the random effect $b_{i0,1}$, $b_{i1,1}$, $b_{i0,2}$ and $b_{i1,2}$, respectively. As σ_{12}, σ_{13}, σ_{14}, we denote the covariance of the random intercept $b_{i0,1}$ with the random effect $b_{i1,1}$, $b_{i0,2}$ and $b_{i1,2}$, respectively, and as σ_{23}, σ_{24}, we denote the covariance of the random slope $b_{i1,1}$ with the random effect $b_{i0,2}$ and $b_{i1,2}$, respectively. We denote as σ_{34} the covariance of the random intercept $b_{i0,2}$ with the random slope $b_{i1,2}$. The models are explained in section 5, subsection 5.2 and presented in Table 3 of subsection 5.4.

Simulation model	Fitted model	Parameter	Real value	Estimate (SD)	Bias	MSE
C+D	C+D	σ_1^2	1.00	1.01 (0.04)	0.01	0.00
		σ_2^2	1.00	1.01 (0.05)	0.01	0.00
		σ^2_{11}	1.00	1.06 (0.21)	0.06	0.05
		σ_{12}	0.00	0.00 (0.11)	-0.01	0.01
		σ_{13}	0.00	0.00 (0.13)	0.00	0.02
		σ_{14}	0.00	-0.01 (0.12)	-0.01	0.01
		σ^2_{22}	1.00	1.05 (0.15)	0.05	0.02
		σ_{23}	0.00	0.00 (0.12)	0.00	0.01
		σ_{24}	0.00	0.00 (0.12)	0.00	0.01
		σ^2_{33}	1.00	1.02 (0.29)	0.02	0.08
		σ_{34}	0.00	0.00 (0.14)	0.00	0.02
		σ^2_{44}	1.00	1.08 (0.19)	0.08	0.04

Table 14: Mean estimates, standard deviation (SD), bias and mean square error (MSE) of the variance parameters of the error terms $\epsilon_{i,1}$ and $\epsilon_{i,2}$ (denoted as σ_1^2 and σ_2^2, respectively) and of the parameters of the variance-covariance matrices $\boldsymbol{D_1}$ and $\boldsymbol{D_2}$ of the random effects of the fitted multivariate mixed-effects model C+D1D2. While the simulation model that generated the data is the multivariate mixed-effects model C+D. We denote as $\sigma_{11,1}^2$, $\sigma_{22,1}^2$, $\sigma_{11,2}^2$ and $\sigma_{22,2}^2$ the variance of the random effect $b_{i0,1}$, $b_{i1,1}$, $b_{i0,2}$ and $b_{i1,2}$, respectively. As $\sigma_{12,1}$, we denote the covariance of the random intercept $b_{i0,1}$ with the random slope $b_{i1,1}$ and as $\sigma_{12,2}$ the covariance of the random intercept $b_{i0,2}$ with the random slope $b_{i1,2}$. The models are explained in section 5, subsection 5.2 and presented in Table 3 of subsection 5.4.

Simulation model	Fitted model	Parameter	Real value	Estimate (SD)	Bias	MSE
C+D	C+D1D2	σ_1^2	1.00	1.00 (0.04)	0.00	0.00
		σ_2^2	1.00	1.01 (0.05)	0.01	0.00
		$\sigma_{11,1}^2$	1.00	1.05 (0.20)	0.05	0.04
		$\sigma_{12,1}$	0.00	-0.01 (0.11)	-0.01	0.01
		$\sigma_{22,1}^2$	1.00	1.04 (0.14)	0.04	0.02
		$\sigma_{11,2}^2$	1.00	1.01 (0.27)	0.01	0.07
		$\sigma_{12,2}$	0.00	0.00 (0.14)	0.00	0.02
		$\sigma_{22,2}^2$	1.00	1.05 (0.19)	0.05	0.04

Table 15: Mean estimates, standard deviation (SD), bias and mean square error (MSE) of the variance parameters of the error terms $\epsilon_{i,1}$ and $\epsilon_{i,2}$ (denoted as σ_1^2 and σ_2^2, respectively) and of the parameters of the variance-covariance matrix $\boldsymbol{D}$ of the random effects of the fitted multivariate mixed-effect models D. While the simulation model that generated the data is the multivariate mixed-effects model C+D. We denote as σ_{11}^2, σ_{22}^2, σ_{33}^2 and σ_{44}^2 the variance of the random effect $b_{i0,1}$, $b_{i1,1}$, $b_{i0,2}$ and $b_{i1,2}$, respectively. As σ_{12}, σ_{13}, σ_{14}, we denote the covariance of the random intercept $b_{i0,1}$ with the random effect $b_{i1,1}$, $b_{i0,2}$ and $b_{i1,2}$, respectively, and as σ_{23}, σ_{24}, we denote the covariance of the random slope $b_{i1,1}$ with the random effect $b_{i0,2}$ and $b_{i1,2}$, respectively. We denote as σ_{34} the covariance of the random intercept $b_{i0,2}$ with the random slope $b_{i1,2}$. The models are explained in section 5, subsection 5.2 and presented in Table 3 of subsection 5.4.

Simulation model	Fitted model	Parameter	Real value	Estimate (SD)	Bias	MSE
C+D	D	σ_1^2	1.00	1.00 (0.04)	0.00	0.00
		σ_2^2	1.00	8.91(0.76)	7.91	63.15
		σ_{11}^2	1.00	1.10 (0.25)	0.10	0.07
		σ_{12}	0.00	-0.02 (0.10)	-0.01	0.01
		σ_{13}	0.00	−0.35(0.73)	−0.35	0.65
		σ_{14}	0.00	0.98(0.59)	0.98	1.30
		σ_{22}^2	1.00	0.93 (0.13)	-0.08	0.02
		σ_{23}	0.00	−5.83(1.10)	−5.83	35.23
		σ_{24}	0.00	5.00(0.77)	5.00	25.61
		σ_{33}^2	1.00	42.86(10.82)	41.86	1869.04
		σ_{34}	0.00	−34.15(7.03)	−34.15	1215.18
		σ_{44}^2	1.00	30.24(4.90)	29.24	878.89

Table 16: Mean estimates, standard deviation (SD), bias and mean square error (MSE) of the variance parameters of the error terms $\epsilon_{i,1}$ and $\epsilon_{i,2}$ (denoted as σ_1^2 and σ_2^2, respectively) and of the parameters of the variance-covariance matrices $\boldsymbol{D_1}$ and $\boldsymbol{D_2}$ of the random effects of the fitted multivariate mixed-effects model C+D1D2. While the simulation model that generated the data is the multivariate mixed-effects model C+D1D2. We denote as $\sigma^2_{11,1}$, $\sigma^2_{22,1}$, $\sigma^2_{11,2}$ and $\sigma^2_{22,2}$ the variance of the random effect $b_{i0,1}$, $b_{i1,1}$, $b_{i0,2}$ and $b_{i1,2}$, respectively. As $\sigma_{12,1}$, we denote the covariance of the random intercept $b_{i0,1}$ with the random slope $b_{i1,1}$ and as $\sigma_{12,2}$ the covariance of the random intercept $b_{i0,2}$ with the random slope $b_{i1,2}$. The models are explained in section 5, subsection 5.2 and presented in Table 3 of subsection 5.4.

Simulation model	Fitted model	Parameter	Real value	Estimate (SD)	Bias	MSE
C+D1D2	C+D1D2	σ_1^2	1.00	1.00 (0.04)	0.00	0.00
		σ_2^2	1.00	1.01 (0.05)	0.01	0.00
		$\sigma^2_{11,1}$	1.00	1.04 (0.20)	0.04	0.04
		$\sigma_{12,1}$	0.00	0.00 (0.12)	0.00	0.01
		$\sigma^2_{22,1}$	1.00	1.05 (0.16)	0.05	0.03
		$\sigma^2_{11,2}$	1.00	1.05 (0.28)	0.05	0.08
		$\sigma_{12,2}$	0.00	0.01 (0.14)	0.01	0.02
		$\sigma^2_{22,2}$	1.00	1.05 (0.17)	0.05	0.03

Table 17: Mean estimates, standard deviation (SD), bias and mean square error (MSE) of the variance parameters of the error terms $\epsilon_{i,1}$ and $\epsilon_{i,2}$ (denoted as σ_1^2 and σ_2^2, respectively) and of the parameters of the variance-covariance matrix $\boldsymbol{D}$ of the random effects of the fitted multivariate mixed-effect models C+D. While the simulation model that generated the data is the multivariate mixed-effects model C+D1D2. We denote as σ^2_{11}, σ^2_{22}, σ^2_{33} and σ^2_{44} the variance of the random effect $b_{i0,1}$, $b_{i1,1}$, $b_{i0,2}$ and $b_{i1,2}$, respectively. As σ_{12}, σ_{13}, σ_{14}, we denote the covariance of the random intercept $b_{i0,1}$ with the random effect $b_{i1,1}$, $b_{i0,2}$ and $b_{i1,2}$, respectively, and as σ_{23}, σ_{24}, we denote the covariance of the random slope $b_{i1,1}$ with the random effect $b_{i0,2}$ and $b_{i1,2}$, respectively. We denote as σ_{34} the covariance of the random intercept $b_{i0,2}$ with the random slope $b_{i1,2}$. The models are explained in section 5, subsection 5.2 and presented in Table 3 of subsection 5.4.

Simulation model	Fitted model	Parameter	Real value	Estimate (SD)	Bias	MSE
C+D1D2	C+D	σ_1^2	1.00	1.01 (0.04)	0.01	0.00
		σ_2^2	1.00	1.01 (0.05)	0.01	0.00
		σ^2_{11}	1.01	1.05 (0.21)	0.05	0.05
		σ_{12}	0.00	0.00 (0.12)	0.00	0.01
		σ_{13}	0.00	0.01 (0.13)	0.01	0.02
		σ_{14}	0.00	0.00 (0.13)	0.00	0.02
		σ^2_{22}	1.00	1.06 (0.17)	0.06	0.03
		σ_{23}	0.00	0.00 (0.13)	0.00	0.02
		σ_{24}	0.00	0.00 (0.12)	0.00	0.01
		σ^2_{33}	1.00	1.05 (0.29)	0.05	0.09
		σ_{34}	0.00	0.00 (0.13)	0.00	0.02
		σ^2_{44}	1.00	1.07 (0.18)	0.07	0.04

Table 18: Mean estimates, standard deviation (SD), bias and mean square error (MSE) of the variance parameters of the error terms $\epsilon_{i,1}$ and $\epsilon_{i,2}$ (denoted as σ_1^2 and σ_2^2, respectively) and of the parameters of the variance-covariance matrices $\boldsymbol{D_1}$ and $\boldsymbol{D_2}$ of the random effects of the fitted multivariate mixed-effects model D1D2. While the simulation model that generated the data is the multivariate mixed-effects model C+D1D2. We denote as $\sigma^2_{11,1}$, $\sigma^2_{22,1}$, $\sigma^2_{11,2}$ and $\sigma^2_{22,2}$ the variance of the random effect $b_{i0,1}$, $b_{i1,1}$, $b_{i0,2}$ and $b_{i1,2}$, respectively. As $\sigma_{12,1}$, we denote the covariance of the random intercept $b_{i0,1}$ with the random slope $b_{i1,1}$ and as $\sigma_{12,2}$ the covariance of the random intercept $b_{i0,2}$ with the random slope $b_{i1,2}$. The models are explained in section 5, subsection 5.2 and presented in Table 3 of subsection 5.4.

Simulation model	Fitted model	Parameter	Real value	Estimate (SD)	Bias	MSE
C+D1D2	D1D2	σ_1^2	1.00	1.01 (0.04)	0.01	0.00
		σ_2^2	1.00	8.94(0.76)	7.94	63.52
		$\sigma^2_{11,1}$	1.00	1.03 (0.23)	0.03	0.05
		$\sigma_{12,1}$	0.00	0.00 (0.12)	0.00	0.01
		$\sigma^2_{22,1}$	1.00	1.05 (0.16)	0.05	0.03
		$\sigma^2_{11,2}$	1.00	41.61(10.88)	40.61	1767.15
		$\sigma_{12,2}$	0.00	−34.95(7.17)	−34.95	1272.50
		$\sigma^2_{22,2}$	1.00	31.39(5.09)	30.39	949.60

Table 19: Mean estimates, standard deviation (SD), bias and mean square error (MSE) of the variance parameters of the error terms $\epsilon_{i,1}$ and $\epsilon_{i,2}$ (denoted as σ_1^2 and σ_2^2, respectively) and of the parameters of the variance-covariance matrix $\boldsymbol{D}$ of the random effects of the fitted multivariate mixed-effect models V+C+D. While the simulation model that generated the data is the multivariate mixed-effects model V+C+D. WWe denote as σ^2_{11}, σ^2_{22}, σ^2_{33} and σ^2_{44} the variance of the random effect $b_{i0,1}$, $b_{i1,1}$, $b_{i0,2}$ and $b_{i1,2}$, respectively. As σ_{12}, σ_{13}, σ_{14}, we denote the covariance of the random intercept $b_{i0,1}$ with the random effect $b_{i1,1}$, $b_{i0,2}$ and $b_{i1,2}$, respectively, and as σ_{23}, σ_{24}, we denote the covariance of the random slope $b_{i1,1}$ with the random effect $b_{i0,2}$ and $b_{i1,2}$, respectively. We denote as σ_{34} the covariance of the random intercept $b_{i0,2}$ with the random slope $b_{i1,2}$. The models are explained in section 5, subsection 5.2 and presented in Table 3 of subsection 5.4.

Simulation model	Fitted model	Parameter	Real value	Estimate (SD)	Bias	MSE
V+C+D	V+C+D	σ_1^2	1.00	1.01 (0.04)	0.01	0.00
		σ_2^2	1.00	1.01 (0.05)	0.01	0.00
		σ^2_{11}	1.00	1.05 (0.20)	0.05	0.04
		σ_{12}	0.00	0.00 (0.10)	0.00	0.01
		σ_{13}	0.00	0.00 (0.09)	-0.01	0.01
		σ_{14}	0.00	-0.01 (0.12)	0.01	0.01
		σ^2_{22}	1.00	1.05 (0.15)	0.05	0.02
		σ_{23}	0.00	0.00 (0.13)	0.01	0.02
		σ_{24}	0.00	-0.05 (0.14)	-0.06	0.02
		σ^2_{33}	1.00	0.99 (0.49)	-0.01	0.24
		σ_{34}	0.00	-0.01 (0.16)	-0.01	0.03
		σ^2_{44}	1.00	1.19 (0.21)	0.19	0.08

Table 20: Mean estimates, standard deviation (SD), bias and mean square error (MSE) of the variance parameters of the error terms $\epsilon_{i,1}$ and $\epsilon_{i,2}$ (denoted as σ_1^2 and σ_2^2, respectively) and of the parameters of the variance-covariance matrices $\boldsymbol{D_1}$ and $\boldsymbol{D_2}$ of the random effects of the fitted multivariate mixed-effects model V+C+D1D2. While the simulation model that generated the data is the multivariate mixed-effects model V+C+D. We denote as $\sigma^2_{11,1}$, $\sigma^2_{22,1}$, $\sigma^2_{11,2}$ and $\sigma^2_{22,2}$ the variance of the random effect $b_{i0,1}$, $b_{i1,1}$, $b_{i0,2}$ and $b_{i1,2}$, respectively. As $\sigma_{12,1}$, we denote the covariance of the random intercept $b_{i0,1}$ with the random slope $b_{i1,1}$ and as $\sigma_{12,2}$ the covariance of the random intercept $b_{i0,2}$ with the random slope $b_{i1,2}$. The models are explained in section 5, subsection 5.2 and presented in Table 3 of subsection 5.4.

Simulation model	Fitted model	Parameter	Real value	Estimate (SD)	Bias	MSE
V+C+D	V+C+D1D2	σ_1^2	1.00	1.01 (0.04)	0.01	0.00
		σ_2^2	1.00	1.01 (0.05)	0.01	0.00
		$\sigma^2_{11,1}$	1.00	1.05 (0.18)	0.05	0.03
		$\sigma_{12,1}$	0.00	-0.01 (0.10)	-0.01	0.01
		$\sigma^2_{22,1}$	1.00	1.04 (0.15)	0.04	0.02
		$\sigma^2_{11,2}$	1.00	0.99 (0.45)	-0.01	0.20
		$\sigma_{12,2}$	0.00	0.00 (0.17)	0.00	0.03
		$\sigma^2_{22,2}$	1.00	1.05 (0.19)	0.05	0.04

Table 21: Mean estimates, standard deviation (SD), bias and mean square error (MSE) of the variance parameters of the error terms $\epsilon_{i,1}$ and $\epsilon_{i,2}$ (denoted as σ_1^2 and σ_2^2, respectively) and of the parameters of the variance-covariance matrix $\boldsymbol{D}$ of the random effects of the fitted multivariate mixed-effect models D. While the simulation model that generated the data is the multivariate mixed-effects model V+C+D. We denote as σ^2_{11}, σ^2_{22}, σ^2_{33} and σ^2_{44} the variance of the random effect $b_{i0,1}$, $b_{i1,1}$, $b_{i0,2}$ and $b_{i1,2}$, respectively. As σ_{12}, σ_{13}, σ_{14}, we denote the covariance of the random intercept $b_{i0,1}$ with the random effect $b_{i1,1}$, $b_{i0,2}$ and $b_{i1,2}$, respectively, and as σ_{23}, σ_{24}, we denote the covariance of the random slope $b_{i1,1}$ with the random effect $b_{i0,2}$ and $b_{i1,2}$, respectively. We denote as σ_{34} the covariance of the random intercept $b_{i0,2}$ with the random slope $b_{i1,2}$. The models are explained in section 5, subsection 5.2 and presented in Table 3 of subsection 5.4.

Simulation model	Fitted model	Parameter	Real value	Estimate (SD)	Bias	MSE
V+C+D	D	σ_1^2	1.00	1.00 (0.04)	0.00	0.00
		σ_2^2	1.00	8.98(0.76)	7.98	64.28
		σ^2_{11}	1.00	1.13 (0.25)	0.13	0.08
		σ_{12}	0.00	-0.01 (0.10)	-0.01	0.01
		σ_{13}	0.00	0.81(0.76)	0.81	1.24
		σ_{14}	0.00	1.10(0.74)	1.10	1.76
		σ^2_{22}	1.00	0.92 (0.13)	-0.08	0.02
		σ_{23}	0.00	−5.79(1.10)	5.79	34.74
		σ_{24}	0.00	6.50(0.97)	6.50	43.17
		σ^2_{33}	1.00	42.91(10.77)	41.91	1872.02
		σ_{34}	0.00	−42.05(8.73)	42.05	1844.16
		σ^2_{44}	1.00	49.14(7.66)	48.14	2375.37

Table 22: Mean estimates, standard deviation (SD), bias and mean square error (MSE) of the variance parameters of the error terms $\epsilon_{i,1}$ and $\epsilon_{i,2}$ (denoted as σ_1^2 and σ_2^2, respectively) and of the parameters of the variance-covariance matrices $\boldsymbol{D_1}$ and $\boldsymbol{D_2}$ of the random effects of the fitted multivariate mixed-effects model V+C+D1D2. While the simulation model that generated the data is the multivariate mixed-effects model V+C+D1D2. We denote as $\sigma^2_{11,1}$, $\sigma^2_{22,1}$, $\sigma^2_{11,2}$ and $\sigma^2_{22,2}$ the variance of the random effect $b_{i0,1}$, $b_{i1,1}$, $b_{i0,2}$ and $b_{i1,2}$, respectively. As $\sigma_{12,1}$, we denote the covariance of the random intercept $b_{i0,1}$ with the random slope $b_{i1,1}$ and as $\sigma_{12,2}$ the covariance of the random intercept $b_{i0,2}$ with the random slope $b_{i1,2}$. The models are explained in section 5, subsection 5.2 and presented in Table 3 of subsection 5.4.

Simulation model	Fitted model	Parameter	Real value	Estimate (SD)	Bias	MSE
V+C+D1D2	V+C+D1D2	σ_1^2	1.00	1.01 (0.04)	0.01	0.00
		σ_2^2	1.00	1.01 (0.05)	0.01	0.00
		$\sigma^2_{11,1}$	1.00	1.04 (0.18)	0.04	0.03
		$\sigma_{12,1}$	0.00	0.00 (0.10)	0.00	0.01
		$\sigma^2_{22,1}$	1.00	1.04 (0.16)	0.04	0.03
		$\sigma^2_{11,2}$	1.00	1.05 (0.44)	0.05	0.19
		$\sigma_{12,2}$	0.00	0.02 (0.16)	0.02	0.03
		$\sigma^2_{22,2}$	1.00	1.05 (0.17)	0.05	0.03

Table 23: Mean estimates, standard deviation (SD), bias and mean square error (MSE) of the variance parameters of the error terms $\epsilon_{i,1}$ and $\epsilon_{i,2}$ (denoted as σ_1^2 and σ_2^2, respectively) and of the parameters of the variance-covariance matrix $\boldsymbol{D}$ of the random effects of the fitted multivariate mixed-effect models V+C+D. While the simulation model that generated the data is the multivariate mixed-effects model V+C+D1D2. We denote as σ^2_{11}, σ^2_{22}, σ^2_{33} and σ^2_{44} the variance of the random effect $b_{i0,1}$, $b_{i1,1}$, $b_{i0,2}$ and $b_{i1,2}$, respectively. As σ_{12}, σ_{13}, σ_{14}, we denote the covariance of the random intercept $b_{i0,1}$ with the random effect $b_{i1,1}$, $b_{i0,2}$ and $b_{i1,2}$, respectively, and as σ_{23}, σ_{24}, we denote the covariance of the random slope $b_{i1,1}$ with the random effect $b_{i0,2}$ and $b_{i1,2}$, respectively. We denote as σ_{34} the covariance of the random intercept $b_{i0,2}$ with the random slope $b_{i1,2}$. The models are explained in section 5, subsection 5.2 and presented in Table 3 of subsection 5.4.

Simulation model	Fitted model	Parameter	Real value	Estimate (SD)	Bias	MSE
V+C+D1D2	V+C+D	σ_1^2	1.00	1.01 (0.04)	0.01	0.00
		σ_2^2	1.00	1.01 (0.05)	0.01	0.00
		σ^2_{11}	1.00	1.04 (0.20)	0.04	0.04
		σ_{12}	0.00	-0.01 (0.11)	-0.01	0.01
		σ_{13}	0.00	-0.01 (0.10)	0.00	0.01
		σ_{14}	0.00	0.01 (0.12)	0.01	0.02
		σ^2_{22}	1.00	1.04 (0.16)	0.04	0.03
		σ_{23}	0.00	0.01 (0.16)	0.01	0.03
		σ_{24}	0.00	-0.06 (0.13)	-0.06	0.02
		σ^2_{33}	1.00	1.04 (0.50)	0.04	0.25
		σ_{34}	0.00	0.00 (0.15)	0.00	0.02
		σ^2_{44}	1.00	1.18(0.19)	0.18	0.07

Table 24: Mean estimates, standard deviation (SD), bias and mean square error (MSE) of the variance parameters of the error terms $\epsilon_{i,1}$ and $\epsilon_{i,2}$ (denoted as σ_1^2 and σ_2^2, respectively) and of the parameters of the variance-covariance matrices $\boldsymbol{D_1}$ and $\boldsymbol{D_2}$ of the random effects of the fitted multivariate mixed-effects model D1D2. While the simulation model that generated the data is the multivariate mixed-effects model V+C+D1D2. We denote as $\sigma_{11,1}^2$, $\sigma_{22,1}^2$, $\sigma_{11,2}^2$ and $\sigma_{22,2}^2$ the variance of the random effect $b_{i0,1}$, $b_{i1,1}$, $b_{i0,2}$ and $b_{i1,2}$, respectively. As $\sigma_{12,1}$, we denote the covariance of the random intercept $b_{i0,1}$ with the random slope $b_{i1,1}$ and as $\sigma_{12,2}$ the covariance of the random intercept $b_{i0,2}$ with the random slope $b_{i1,2}$. The models are explained in section 5, subsection 5.2 and presented in Table 3 of subsection 5.4.

Simulation model	Fitted model	Parameter	Real value	Estimate (SD)	Bias	MSE
V+C+D1D2	D1D2	σ_1^2	1.00	1.01 (0.04)	0.01	0.00
		σ_2^2	1.00	9.07(0.74) 8.07	8.07	65.69
		$\sigma_{11,1}^2$	1.00	1.03 (0.23)	0.03	0.05
		$\sigma_{12,1}$	0.00	0.00 (0.12)	0.00	0.01
		$\sigma_{22,1}^2$	1.00	1.05 (0.16)	0.05	0.03
		$\sigma_{11,2}^2$	1.00	38.93(10.78)	37.93	1554.54
		$\sigma_{12,2}$	0.00	−42.59(9.03)	−42.59	1894.60
		$\sigma_{22,2}^2$	1.00	51.11(8.09)	50.11	2576.33

Part F

Results of extra simulation

For this simulation study, the same parameter values and priors are assumed as for the simulation study presented in the manuscript with the only exception that for the simulation models V+D, C+D and V+C+D, it is assumed that the variance-covariance matrix is

$$\boldsymbol{D} = \begin{bmatrix} 1 & 0 & 0.3 & 0.4 \\ 0 & 1 & 0.1 & 0.2 \\ 0.3 & 0.1 & 1 & 0 \\ 0.4 & 0.2 & 0 & 1 \end{bmatrix}$$

In the Tables below, we present the results of these models for each scenario. For the models V+D1D2, C+D1D2 and V+C+D1D2 the results did not change and are similar to the results presented in the tables above.

Table 25: Mean estimates, standard deviation (SD), bias and mean square error (MSE) of the fixed parameters of the fitted multivariate mixed-effects models V+D, V+D1D2 and D. While the simulation model that generated the data is the multivariate mixed-effects model V+D. The explanation of the notations used for the models and the coefficients is given in section 5, subsection 5.2.

Simulation model	Fitted model	Parameter	Real value	Estimate (SD)	Bias	MSE
V+D	V+D	$\beta_{0,1}$	3.01	3.01 (0.18)	-0.00	0.03
		$\beta_{1,1}$	0.47	0.47 (0.09)	0.00	0.01
		$\beta_{2,1}$	1.50	1.49 (0.24)	-0.01	0.06
		$\beta_{0,2}$	2.15	1.17(0.49)	−0.98	1.21
		$\beta_{1,2}$	0.91	0.75 (0.12)	-0.16	0.04
		$\beta_{2,2}$	1.30	0.84 (0.45)	-0.46	0.41
		α_1	1.63	1.95(0.14)	0.32	0.12
	V+D1D2	$\beta_{0,1}$	3.01	3.01 (0.19)	-0.00	0.04
		$\beta_{1,1}$	0.47	0.47 (0.10)	0.00	0.01
		$\beta_{2,1}$	1.50	1.49 (0.26)	-0.01	0.07
		$\beta_{0,2}$	2.15	1.39 (0.38)	-0.76	0.74
		$\beta_{1,2}$	0.91	0.79 (0.10)	-0.12	0.02
		$\beta_{2,2}$	1.30	0.96 (0.40)	-0.34	0.24
		α_1	1.63	1.88 (0.09)	0.25	0.07
	D	$\beta_{0,1}$	3.01	3.01 (0.18)	-0.00	0.03
		$\beta_{1,1}$	0.47	0.47 (0.10)	-0.00	0.01
		$\beta_{2,1}$	1.50	1.50 (0.25)	-0.00	0.06
		$\beta_{0,2}$	2.15	7.04(0.30)	4.89	24.04
		$\beta_{1,2}$	0.91	1.67(0.21)	0.76	0.62
		$\beta_{2,2}$	1.30	3.77(0.43)	2.47	6.28

Table 26: Mean estimates, standard deviation (SD), bias and mean square error (MSE) of the fixed parameters of the fitted multivariate mixed-effects models C+D, C+D1D2 and D. While the simulation model that generated the data is the multivariate mixed-effects model C+D. The explanation of the notations used for the models and the coefficients is given in section 5, subsection 5.2.

Simulation model	Fitted model	Parameter	Real value	Estimate (SD)	Bias	MSE
C+D	C+D	$\beta_{0,1}$	3.01	3.00 (0.16)	-0.01	0.03
		$\beta_{1,1}$	0.47	0.47 (0.10)	0.00	0.01
		$\beta_{2,1}$	1.50	1.51 (0.23)	0.01	0.05
		$\beta_{0,2}$	2.15	2.13 (0.18)	-0.02	0.03
		$\beta_{1,2}$	0.91	0.92 (0.12)	0.01	0.02
		$\beta_{2,2}$	1.30	1.32 (0.25)	0.02	0.06
		α_1	0.90	0.90 (0.01)	0.00	0.00
	C+D1D2	$\beta_{0,1}$	3.01	3.02 (0.17)	0.01	0.03
		$\beta_{1,1}$	0.47	0.47 (0.10)	-0.00	0.01
		$\beta_{2,1}$	1.50	1.51 (0.25)	0.01	0.06
		$\beta_{0,2}$	2.15	2.14 (0.18)	-0.01	0.03
		$\beta_{1,2}$	0.91	0.87 (0.12)	-0.04	0.02
		$\beta_{2,2}$	1.30	1.32 (0.26)	0.02	0.07
		α_1	0.90	0.91 (0.01)	0.01	0.00
	D	$\beta_{0,1}$	3.01	3.25 (0.16)	0.24	0.09
		$\beta_{1,1}$	0.47	0.47 (0.10)	-0.00	0.01
		$\beta_{2,1}$	1.5	0.99(0.20)	−0.51	0.30
		$\beta_{0,2}$	2.15	−2.03(0.90)	−4.18	18.25
		$\beta_{1,2}$	0.91	6.87(0.56)	5.96	35.84
		$\beta_{2,2}$	1.3	3.23(0.83)	1.93	4.42

Table 27: Mean estimates, standard deviation (SD), bias and mean square error (MSE) of the fixed parameters of the fitted multivariate mixed-effects models V+C+D, V+C+D1D2 and D. While the simulation model that generated the data is the multivariate mixed-effects model V+C+D. The explanation of the notations used for the models and the coefficients is given in section 5, subsection 5.2.

Simulation model	Fitted model	Parameter	Real value	Estimate (SD)	Bias	MSE
V+C+D	V+C+D	$\beta_{0,1}$	3.01	3.00 (0.16)	-0.01	0.02
		$\beta_{1,1}$	0.47	0.47 (0.10)	0.00	0.01
		$\beta_{2,1}$	1.50	1.51 (0.23)	0.01	0.05
		$\beta_{0,2}$	2.15	1.29(0.49)	−0.86	0.99
		$\beta_{1,2}$	0.91	0.78 (0.13)	-0.13	0.03
		$\beta_{2,2}$	1.30	0.89 (0.38)	-0.41	0.31
		α_{11}	1.63	1.91 (0.14)	0.28	0.10
		α_{21}	0.90	0.90 (0.01)	-0.00	0.00
	V+C+D1D2	$\beta_{0,1}$	3.01	3.01 (0.17)	0.00	0.03
		$\beta_{1,1}$	0.47	0.47 (0.09)	0.00	0.01
		$\beta_{2,1}$	1.50	1.51 (0.25)	0.01	0.06
		$\beta_{0,2}$	2.15	1.51 (0.42)	-0.64	0.59
		$\beta_{1,2}$	0.91	0.81 (0.12)	-0.10	0.03
		$\beta_{2,2}$	1.30	1.03 (0.37)	-0.27	0.21
		α_{11}	1.63	1.83 (0.12)	0.20	0.05
		α_{21}	0.90	0.90 (0.01)	0.00	0.00
	D	$\beta_{0,1}$	3.01	3.26 (0.16)	0.25	0.09
		$\beta_{1,1}$	0.47	0.47 (0.09)	-0.00	0.01
		$\beta_{2,1}$	1.5	0.99(0.19)	−0.51	0.30
		$\beta_{0,2}$	2.15	3.13(0.93)	0.98	1.81
		$\beta_{1,2}$	0.91	7.65(0.70)	6.74	45.88
		$\beta_{2,2}$	1.30	5.11(0.97)	3.81	15.47

Table 28: Mean estimates, standard deviation (SD), bias and mean square error (MSE) of the variance parameters of the error terms $\epsilon_{i,1}$ and $\epsilon_{i,2}$ (denoted as σ_1^2 and σ_2^2, respectively) and of the parameters of the variance-covariance matrix $\boldsymbol{D}$ of the random effects of the fitted multivariate mixed-effects model V+D. While the simulation model that generated the data is the multivariate mixed-effects model V+D. We denote as σ_{11}^2, σ_{22}^2, σ_{33}^2 and σ_{44}^2 the variance of the random effect $b_{i0,1}$, $b_{i1,1}$, $b_{i0,2}$ and $b_{i1,2}$, respectively. As σ_{12}, σ_{13}, σ_{14}, we denote the covariance of the random intercept $b_{i0,1}$ with the random effect $b_{i1,1}$, $b_{i0,2}$ and $b_{i1,2}$, respectively, and as σ_{23}, σ_{24}, we denote the covariance of the random slope $b_{i1,1}$ with the random effect $b_{i0,2}$ and $b_{i1,2}$, respectively. We denote as σ_{34} the covariance of the random intercept $b_{i0,2}$ with the random slope $b_{i1,2}$. The models are explained in section 5, subsection 5.2 and presented in Table 3 of subsection 5.4.

Simulation model	Fitted model	Parameter	Real value	Estimate (SD)	Bias	MSE
V+D	V+D	σ_1^2	1.00	1.01 (0.04)	0.01	0.03
		σ_2^2	1.00	1.00 (0.04)	0.00	0.01
		σ_{11}^2	1.00	1.03 (0.21)	0.03	0.04
		σ_{12}	0.00	0.02 (0.11)	0.02	0.01
		σ_{13}	0.00	0.03 (0.09)	-0.27	0.08
		σ_{14}	0.00	0.37 (0.13)	-0.03	0.02
		σ_{22}^2	1.00	1.06 (0.16)	0.06	0.02
		σ_{23}	0.00	0.07 (0.17)	-0.03	0.02
		σ_{24}	0.00	-0.12 (0.14)	-0.32	0.12
		σ_{33}^2	1.00	0.97 (0.47)	-0.03	0.22
		σ_{34}	0.00	-0.01 (0.14)	-0.10	0.03
		σ_{44}^2	1.00	1.13(0.16)	0.13	0.04

Table 29: Mean estimates, standard deviation (SD), bias and mean square error (MSE) of the variance parameters of the error terms $\epsilon_{i,1}$ and $\epsilon_{i,2}$ (denoted as σ_1^2 and σ_2^2, respectively) and of the parameters of the variance-covariance matrices $\boldsymbol{D_1}$ and $\boldsymbol{D_2}$ of the random effects of the fitted multivariate mixed-effects model V+D1D2. While the simulation model that generated the data is the multivariate mixed-effects model V+D. We denote as $\sigma_{11,1}^2$, $\sigma_{22,1}^2$, $\sigma_{11,2}^2$ and $\sigma_{22,2}^2$ the variance of the random effect $b_{i0,1}$, $b_{i1,1}$, $b_{i0,2}$ and $b_{i1,2}$, respectively. As $\sigma_{12,1}$, we denote the covariance of the random intercept $b_{i0,1}$ with the random slope $b_{i1,1}$ and as $\sigma_{12,2}$ the covariance of the random intercept $b_{i0,2}$ with the random slope $b_{i1,2}$. The models are explained in section 5, subsection 5.2 and presented in Table 3 of subsection 5.4.

Simulation model	Fitted model	Parameter	Real value	Estimate (SD)	Bias	MSE
V+D	V+D1D2	σ_1^2	1.00	1.01 (0.04)	0.01	0.00
		σ_2^2	1.00	1.00 (0.04)	0.00	0.00
		$\sigma_{11,1}^2$	1.00	1.03 (0.19)	0.03	0.04
		$\sigma_{12,1}$	0.00	0.04 (0.11)	0.04	0.01
		$\sigma_{22,1}^2$	1.00	1.04 (0.15)	0.04	0.02
		$\sigma_{11,2}^2$	1.00	1.10 (0.44)	0.10	0.21
		$\sigma_{12,2}$	0.00	-0.14 (0.15)	0.14	0.04
		$\sigma_{22,2}^2$	1.00	0.94 (0.14)	-0.06	0.02

Table 30: Mean estimates, standard deviation (SD), bias and mean square error (MSE) of the variance parameters of the error terms $\epsilon_{i,1}$ and $\epsilon_{i,2}$ (denoted as σ_1^2 and σ_2^2, respectively) and of the parameters of the variance-covariance matrix $\boldsymbol{D}$ of the random effects of the fitted multivariate mixed-effect models D. While the simulation model that generated the data is the multivariate mixed-effects model V+D. We denote as σ_{11}^2, σ_{22}^2, σ_{33}^2 and σ_{44}^2 the variance of the random effect $b_{i0,1}$, $b_{i1,1}$, $b_{i0,2}$ and $b_{i1,2}$, respectively. As σ_{12}, σ_{13}, σ_{14}, we denote the covariance of the random intercept $b_{i0,1}$ with the random effect $b_{i1,1}$, $b_{i0,2}$ and $b_{i1,2}$, respectively, and as σ_{23}, σ_{24}, we denote the covariance of the random slope $b_{i1,1}$ with the random effect $b_{i0,2}$ and $b_{i1,2}$, respectively. We denote as σ_{34} the covariance of the random intercept $b_{i0,2}$ with the random slope $b_{i1,2}$. The models are explained in section 5, subsection 5.2 and presented in Table 3 of subsection 5.4.

Simulation model	Fitted model	Parameter	Real value	Estimate (SD)	Bias	MSE
V+D	D	σ_1^2	1.00	1.00 (0.04)	-0.00	0.00
		σ_2^2	1.00	1.00 (0.04)	0.00	0.00
		σ_{11}^2	1.00	1.04 (0.21)	0.04	0.05
		σ_{12}	0.00	-0.00 (0.11)	-0.00	0.01
		σ_{13}	0.00	1.82(0.34)	1.52	2.43
		σ_{14}	0.00	0.40 (0.23)	-0.00	0.05
		σ_{22}^2	1.00	1.00 (0.15)	-0.00	0.02
		σ_{23}	0.00	0.09 (0.22)	-0.01	0.05
		σ_{24}	0.00	1.79(0.28)	1.59	2.62
		σ_{33}^2	1.00	4.73(0.75)	3.73	14.50
		σ_{34}	0.00	0.74 (0.46)	0.74	0.75
		σ_{44}^2	1.00	4.29(0.59)	3.29	11.16

Table 31: Mean estimates, standard deviation (SD), bias and mean square error (MSE) of the variance parameters of the error terms $\epsilon_{i,1}$ and $\epsilon_{i,2}$ (denoted as σ_1^2 and σ_2^2, respectively) and of the parameters of the variance-covariance matrix $\boldsymbol{D}$ of the random effects of the fitted multivariate mixed-effect models C+D. While the simulation model that generated the data is the multivariate mixed-effects model C+D. We denote as σ_{11}^2, σ_{22}^2, σ_{33}^2 and σ_{44}^2 the variance of the random effect $b_{i0,1}$, $b_{i1,1}$, $b_{i0,2}$ and $b_{i1,2}$, respectively. As σ_{12}, σ_{13}, σ_{14}, we denote the covariance of the random intercept $b_{i0,1}$ with the random effect $b_{i1,1}$, $b_{i0,2}$ and $b_{i1,2}$, respectively, and as σ_{23}, σ_{24}, we denote the covariance of the random slope $b_{i1,1}$ with the random effect $b_{i0,2}$ and $b_{i1,2}$, respectively. We denote as σ_{34} the covariance of the random intercept $b_{i0,2}$ with the random slope $b_{i1,2}$. The models are explained in section 5, subsection 5.2 and presented in Table 3 of subsection 5.4.

Simulation model	Fitted model	Parameter	Real value	Estimate (SD)	Bias	MSE
C+D	C+D	σ_1^2	1.00	1.00 (0.04)	0.00	0.00
		σ_2^2	1.00	1.01 (0.05)	0.01	0.00
		σ_{11}^2	1.00	1.09 (0.19)	0.09	0.04
		σ_{12}	0.00	-0.01 (0.11)	-0.01	0.01
		σ_{13}	0.00	0.29 (0.15)	-0.01	0.02
		σ_{14}	0.00	0.39 (0.13)	-0.01	0.01
		σ_{22}^2	1.00	1.06 (0.15)	0.06	0.03
		σ_{23}	0.00	0.10 (0.13)	-0.00	0.02
		σ_{24}	0.00	0.21 (0.13)	0.01	0.02
		σ_{33}^2	1.00	1.03 (0.26)	0.03	0.07
		σ_{34}	0.00	0.02 (0.13)	0.02	0.02
		σ_{44}^2	1.00	1.06 (0.19)	0.06	0.04

Table 32: Mean estimates, standard deviation (SD), bias and mean square error (MSE) of the variance parameters of the error terms $\epsilon_{i,1}$ and $\epsilon_{i,2}$ (denoted as σ_1^2 and σ_2^2, respectively) and of the parameters of the variance-covariance matrices $\boldsymbol{D_1}$ and $\boldsymbol{D_2}$ of the random effects of the fitted multivariate mixed-effects model C+D1D2. While the simulation model that generated the data is the multivariate mixed-effects model C+D. We denote as $\sigma_{11,1}^2$, $\sigma_{22,1}^2$, $\sigma_{11,2}^2$ and $\sigma_{22,2}^2$ the variance of the random effect $b_{i0,1}$, $b_{i1,1}$, $b_{i0,2}$ and $b_{i1,2}$, respectively. As $\sigma_{12,1}$, we denote the covariance of the random intercept $b_{i0,1}$ with the random slope $b_{i1,1}$ and as $\sigma_{12,2}$ the covariance of the random intercept $b_{i0,2}$ with the random slope $b_{i1,2}$. The models are explained in section 5, subsection 5.1, subsection 5.2 and presented in Table 3 of subsection 5.4.

Simulation model	Fitted model	Parameter	Real value	Estimate (SD)	Bias	MSE
C+D	C+D1D2	σ_1^2	0.99	1.00 (0.04)	-0.01	0.00
		σ_2^2	1.00	1.01 (0.05)	0.01	0.00
		$\sigma_{11,1}^2$	1.00	1.24 (0.21)	0.24	0.10
		$\sigma_{12,1}$	0.00	0.05 (0.12)	0.05	0.02
		$\sigma_{22,1}^2$	1.00	1.03 (0.15)	0.03	0.02
		$\sigma_{11,2}^2$	1.00	1.08 (0.26)	0.08	0.08
		$\sigma_{12,2}$	0.00	0.08 (0.14)	0.08	0.03
		$\sigma_{22,2}^2$	1.00	1.12 (0.20)	0.12	0.05

Table 33: Mean estimates, standard deviation (SD), bias and mean square error (MSE) of the variance parameters of the error terms $\epsilon_{i,1}$ and $\epsilon_{i,2}$ (denoted as σ_1^2 and σ_2^2, respectively) and of the parameters of the variance-covariance matrix $\boldsymbol{D}$ of the random effects of the fitted multivariate mixed-effect models D. While the simulation model that generated the data is the multivariate mixed-effects model C+D. We denote as σ_{11}^2, σ_{22}^2, σ_{33}^2 and σ_{44}^2 the variance of the random effect $b_{i0,1}$, $b_{i1,1}$, $b_{i0,2}$ and $b_{i1,2}$, respectively. As σ_{12}, σ_{13}, σ_{14}, we denote the covariance of the random intercept $b_{i0,1}$ with the random effect $b_{i1,1}$, $b_{i0,2}$ and $b_{i1,2}$, respectively, and as σ_{23}, σ_{24}, we denote the covariance of the random slope $b_{i1,1}$ with the random effect $b_{i0,2}$ and $b_{i1,2}$, respectively. We denote as σ_{34} the covariance of the random intercept $b_{i0,2}$ with the random slope $b_{i1,2}$. The models are explained in section 5, subsection 5.2 and presented in Table 3 of subsection 5.4.

Simulation model	Fitted model	Parameter	Real value	Estimate (SD)	Bias	MSE
C+D	D	σ_1^2	1.00	1.00 (0.04)	0.00	0.00
		σ_2^2	1.00	8.91(0.76)	7.91	63.07
		σ_{11}^2	1.00	1.16 (0.25)	0.16	0.09
		σ_{12}	0.00	-0.02 (0.10)	-0.02	0.01
		σ_{13}	0.00	$-0.21(0.80)$	-0.51	0.90
		σ_{14}	0.00	1.44(0.68)	1.04	1.54
		σ_{22}^2	1.00	0.93 (0.13)	-0.07	0.02
		σ_{23}	0.00	$-5.77(1.04)$	-5.87	35.54
		σ_{24}	0.00	5.23(0.79)	5.03	25.90
		σ_{33}^2	1.00	41.50(9.84)	40.50	1736.60
		σ_{34}	0.00	$-34.73(6.87)$	-34.73	1253.08
		σ_{44}^2	1.00	33.28(5.17)	32.28	1068.72

Table 34: Mean estimates, standard deviation (SD), bias and mean square error (MSE) of the variance parameters of the error terms $\epsilon_{i,1}$ and $\epsilon_{i,2}$ (denoted as σ_1^2 and σ_2^2, respectively) and of the parameters of the variance-covariance matrix $\boldsymbol{D}$ of the random effects of the fitted multivariate mixed-effect models V+C+D. While the simulation model that generated the data is the multivariate mixed-effects model V+C+D. WWe denote as σ_{11}^2, σ_{22}^2, σ_{33}^2 and σ_{44}^2 the variance of the random effect $b_{i0,1}$, $b_{i1,1}$, $b_{i0,2}$ and $b_{i1,2}$, respectively. As σ_{12}, σ_{13}, σ_{14}, we denote the covariance of the random intercept $b_{i0,1}$ with the random effect $b_{i1,1}$, $b_{i0,2}$ and $b_{i1,2}$, respectively, and as σ_{23}, σ_{24}, we denote the covariance of the random slope $b_{i1,1}$ with the random effect $b_{i0,2}$ and $b_{i1,2}$, respectively. We denote as σ_{34} the covariance of the random intercept $b_{i0,2}$ with the random slope $b_{i1,2}$. The models are explained in section 5, subsection 5.2 and presented in Table 3 of subsection 5.4.

Simulation model	Fitted model	Parameter	Real value	Estimate (SD)	Bias	MSE
V+C+D	V+C+D	σ_1^2	1.00	1.00 (0.04)	0.00	0.00
		σ_2^2	1.00	1.01 (0.05)	0.02	0.00
		σ_{11}^2	1.00	1.08 (0.19)	0.08	0.04
		σ_{12}	0.00	0.00 (0.11)	0.00	0.01
		σ_{13}	0.00	0.01 (0.10)	-0.29	0.09
		σ_{14}	0.00	0.37 (0.13)	-0.03	0.02
		σ_{22}^2	1.00	1.05 (0.15)	0.05	0.03
		σ_{23}	0.00	0.09 (0.12)	-0.01	0.02
		σ_{24}	0.00	-0.07 (0.14)	-0.27	0.09
		σ_{33}^2	1.00	0.95 (0.46)	-0.05	0.21
		σ_{34}	0.00	-0.09 (0.14)	-0.09	0.03
		σ_{44}^2	1.00	1.11 (0.19)	0.11	0.05

Table 35: Mean estimates, standard deviation (SD), bias and mean square error (MSE) of the variance parameters of the error terms $\epsilon_{i,1}$ and $\epsilon_{i,2}$ (denoted as σ_1^2 and σ_2^2, respectively) and of the parameters of the variance-covariance matrices $\boldsymbol{D_1}$ and $\boldsymbol{D_2}$ of the random effects of the fitted multivariate mixed-effects model V+C+D1D2. While the simulation model that generated the data is the multivariate mixed-effects model V+C+D. We denote as $\sigma^2_{11,1}$, $\sigma^2_{22,1}$, $\sigma^2_{11,2}$ and $\sigma^2_{22,2}$ the variance of the random effect $b_{i0,1}$, $b_{i1,1}$, $b_{i0,2}$ and $b_{i1,2}$, respectively. As $\sigma_{12,1}$, we denote the covariance of the random intercept $b_{i0,1}$ with the random slope $b_{i1,1}$ and as $\sigma_{12,2}$ the covariance of the random intercept $b_{i0,2}$ with the random slope $b_{i1,2}$. The models are explained in section 5, subsection 5.2 and presented in Table 3 of subsection 5.4.

Simulation model	Fitted model	Parameter	Real value	Estimate (SD)	Bias	MSE
V+C+D	V+C+D1D2	σ_1^2	1.00	1.00 (0.04)	-0.00	0.00
		σ_2^2	1.00	1.01 (0.05)	0.01	0.00
		$\sigma^2_{11,1}$	1.00	1.18 (0.19)	0.18	0.07
		$\sigma_{12,1}$	0.00	0.03 (0.11)	0.03	0.01
		$\sigma^2_{22,1}$	1.00	1.04 (0.15)	0.04	0.02
		$\sigma^2_{11,2}$	1.00	0.97 (0.45)	-0.03	0.20
		$\sigma_{12,2}$	0.00	0.10 (0.18)	0.10	0.04
		$\sigma^2_{22,2}$	1.00	1.07 (0.19)	0.07	0.04

Table 36: Mean estimates, standard deviation (SD), bias and mean square error (MSE) of the variance parameters of the error terms $\epsilon_{i,1}$ and $\epsilon_{i,2}$ (denoted as σ_1^2 and σ_2^2, respectively) and of the parameters of the variance-covariance matrix $\boldsymbol{D}$ of the random effects of the fitted multivariate mixed-effect models D. While the simulation model that generated the data is the multivariate mixed-effects model V+C+D. We denote as σ^2_{11}, σ^2_{22}, σ^2_{33} and σ^2_{44} the variance of the random effect $b_{i0,1}$, $b_{i1,1}$, $b_{i0,2}$ and $b_{i1,2}$, respectively. As σ_{12}, σ_{13}, σ_{14}, we denote the covariance of the random intercept $b_{i0,1}$ with the random effect $b_{i1,1}$, $b_{i0,2}$ and $b_{i1,2}$, respectively, and as σ_{23}, σ_{24}, we denote the covariance of the random slope $b_{i1,1}$ with the random effect $b_{i0,2}$ and $b_{i1,2}$, respectively. We denote as σ_{34} the covariance of the random intercept $b_{i0,2}$ with the random slope $b_{i1,2}$. The models are explained in section 5, subsection 5.2 and presented in Table 3 of subsection 5.4.

Simulation model	Fitted model	Parameter	Real value	Estimate (SD)	Bias	MSE
V+C+D	D	σ_1^2	1.00	1.00 (0.04)	-0.00	0.00
		σ_2^2	1.00	8.99(0.76)	7.99	64.40
		σ^2_{11}	1.00	1.18 (0.25)	0.18	0.09
		σ_{12}	0.00	-0.01 (0.11)	-0.01	0.01
		σ_{13}	0.00	1.04(0.84)	0.74	1.25
		σ_{14}	0.00	1.56(0.84)	1.16	2.05
		σ^2_{22}	1.00	0.93 (0.13)	-0.07	0.02
		σ_{23}	0.00	−5.75(1.04)	−5.85	35.33
		σ_{24}	0.00	6.78(1.00)	6.58	44.24
		σ^2_{33}	1.00	42.02(9.66)	41.02	1775.26
		σ_{34}	0.00	−42.03(8.37)	−42.03	1835.83
		σ^2_{44}	1.00	53.28(8.03)	52.28	2797.75
